# Interpretable Modelling of a Streptococcal Exposure–Associated Neuropsychiatric Profile in a Pediatric Cohort

**DOI:** 10.3390/children13101443

**Published:** 2026-10-21

**Authors:** Cristina Gabriela Schiopu, Cristina Elena Dobre, Dumitru Adrian Cantemir, Dan Catalin Oprea, Alexandra Boloș, Oriana-Maria Onicescu, Carmen Gabriela Lupusoru, Bogdan Victor Stefanescu, Ilinca Untu, Cristinel Stefanescu

**Affiliations:** 1 Department of Psychiatry, Grigore T. Popa University of Medicine and Pharmacy Iasi, 16 Universității Str., 700115 Iași, Romania; cristina-gabriela.schiopu@umfiasi.ro (C.G.S.); dumitru.cantemir@umfiasi.ro (D.A.C.); dan.oprea@umfiasi.ro (D.C.O.); carmen-gabriela.lupusoru@umfiasi.ro (C.G.L.); bogdan-victor.stefanescu@d.umfiasi.ro (B.V.S.); ilinca.untu@umfiasi.ro (I.U.); cri.stefanescu@umfiasi.ro (C.S.); 2 “Socola” Institute of Psychiatry, 36 Bucium St., 700282 Iași, Romania; cristina.dobre@socola.eu; 3 Faculty of Computer Science, Alexandru Ioan Cuza University of Iași, 16 General Berthelot St., 700483 Iași, Romania; oriana.oniciuc@info.uaic.ro

**Keywords:** PANS, PANDAS, antistreptolysin O, streptococcal exposure, PANS-31, pediatric neuropsychiatry, complete-information modelling, restricted-feature sensitivity analysis, SHAP, interpretable modelling

## Abstract

**Highlights:**

**What are the main findings?**

- Three interpretable AI models successfully reconstructed a predefined, multidimensional clinical–serological profile in a naturalistic cohort of 96 children and adolescents, integrating neuropsychiatric symptoms, ASO seroreactivity, abrupt clinical change, and recent reported streptococcal infection.
- A revealing sensitivity analysis showed that removing just three endpoint-related predictors reduced held-out ROC-AUC values from 0.909–0.961 to 0.630–0.792, highlighting the substantial dependence of model performance on clinically defining information.

**What are the implications of the main findings?**

- Beyond achieving high classification performance, this study illustrates how explainable AI can critically examine the structure of complex pediatric neuropsychiatric profiles and distinguish internal pattern reconstruction from independent diagnostic validation.
- The residual symptom-related and contextual signals offer promising directions for hypothesis-driven research, providing a methodological foundation for future investigations involving independent cohorts and clinically relevant comparison groups.

**Abstract:**

Background/Objectives: Pediatric neuropsychiatric presentations in the context of suspected previous streptococcal exposure remain clinically heterogeneous. This study examined whether a predefined clinical–serological profile could be reconstructed within a naturalistic psychiatric cohort using interpretable supervised models and whether discrimination persisted after removing endpoint-proximal predictors. Methods: This secondary exploratory analysis included 96 children and adolescents evaluated in Romanian psychiatric inpatient and outpatient settings. The operational endpoint required PANS-31 > 36, antistreptolysin O (ASO) > 200 IU/mL, abrupt symptom onset or worsening, and a reported streptococcal episode within six months; 24 participants met all four conditions. Infection history was derived from caregiver reports and available medical documentation. Logistic Regression, Random Forest, and XGBoost were evaluated using stratified five-fold cross-validation and a 70/30 training–test split. Full-feature models included 27 predictors, whereas restricted-feature models were refitted after excluding continuous ASO, infection recency, and abrupt onset. SHAP was used to examine feature attribution. Results: Full-feature models achieved held-out ROC-AUC values of 0.909–0.961, compared with 0.630–0.792 after feature restriction. The held-out sample comprised 29 participants, including seven endpoint-positive cases. Full-feature attribution emphasized serological, temporal, and onset-related variables, whereas restricted-feature attribution shifted toward symptom-domain, age-related, and contextual variables. Conclusions: High full-feature discrimination primarily reflected internal reconstruction of the rule-defined profile rather than independent diagnostic prediction. The attenuation following feature restriction indicates substantial dependence on information related to endpoint construction. These exploratory findings do not establish streptococcal causality, disease specificity, or generalizable diagnostic performance.

## 1. Introduction

Pediatric acute-onset neuropsychiatric presentations occurring after, or in temporal proximity to, infectious episodes remain clinically challenging and diagnostically heterogeneous, despite increasing clinical and research interest. Children may present with abrupt behavioral change, obsessive compulsive symptoms, tics, eating disturbance, anxiety, emotional lability, cognitive or school decline, sleep disruption, somatic complaints, urinary symptoms, or broader functional deterioration across psychiatric, neurological, somatic, and infectious-disease settings. Pediatric Autoimmune Neuropsychiatric Disorders Associated with Streptococcal Infections (PANDAS) was initially proposed as a narrower streptococcal-associated clinical construct characterized by abrupt onset or exacerbation of obsessive compulsive symptoms and/or tics in temporal association with group A streptococcal infection [1]. Pediatric Acute-onset Neuropsychiatric Syndrome (PANS) subsequently provided a broader clinical framework, defined by abrupt onset of obsessive compulsive symptoms and/or severe restrictive eating together with additional acute neuropsychiatric manifestations, without requiring group A streptococcal infection as the precipitating factor [2,3]. Thus, although the two constructs overlap clinically, they are not interchangeable: streptococcal association is integral to the PANDAS concept, whereas PANS encompasses a broader etiological spectrum. Despite their clinical relevance and the rising scientific interest in this area, both frameworks remain characterized by diagnostic uncertainty, phenotypic heterogeneity, and the absence of disease-specific biomarkers [4,5,6,7].

Markers of previous streptococcal exposure, particularly antistreptolysin O (ASO) and anti-DNase B antibodies, require cautious interpretation. Elevated titers may support previous exposure but cannot independently establish infection timing, a causal relationship with neuropsychiatric symptoms, or a diagnosis of PANS/PANDAS [8,9]. Dimensional symptom assessment is also valuable in this context, particularly because these presentations may extend beyond obsessive compulsive symptoms and tics [10]. The Pediatric Acute-onset Neuropsychiatric Syndrome 31-Item Symptom Rating Scale (PANS-31) provides a multidimensional measure of neuropsychiatric symptom burden with published psychometric support [11].

Interpretable supervised modelling can examine whether a clinically defined symptom–serological pattern is internally recoverable from baseline information. In small, clinically selected cohorts, however, model discrimination may partly reflect overlap between predictors and components of a rule-defined endpoint rather than independent diagnostic information [12,13]. SHAP-based interpretation can help characterize how fitted models use the available variables, without establishing biological causality or disease specificity [14]. Transparent reporting of model development, evaluation, and interpretation is therefore particularly important in exploratory healthcare applications [15].

The present study is a secondary exploratory modelling analysis of a naturalistic Romanian pediatric psychiatric cohort previously characterized in an observational study [16]. We investigated whether an operational profile integrating higher multidimensional symptom burden, elevated ASO, abrupt symptom onset or worsening, and recent reported streptococcal infection could be internally reconstructed using interpretable supervised models. To assess the dependence of discrimination on endpoint-proximal information, we compared full-feature models with restricted-feature models refitted after excluding continuous ASO value, infection recency, and abrupt onset. Our objective was to characterize internal profile recoverability and residual clinical signal, rather than to classify PANS/PANDAS diagnoses, predict microbiologically confirmed GAS infection, or infer streptococcal causality.

## 2. Materials and Methods

### 2.1. Study Design and Analytical Framework

This study used baseline data from an observational Romanian pediatric psychiatric cohort assembled to characterize clinical, serological, psychometric, developmental, neurophysiological, somatic, and infection-related features in children and adolescents with neuropsychiatric symptoms and caregiver- or record-supported history suggestive of previous streptococcal exposure.

The present secondary analysis used the same retained baseline dataset as the previously published naturalistic observational study, without additional participant recruitment or clinical reassessment. Whereas the original study focused on describing clinical, serological, and psychometric associations, the current investigation applied interpretable supervised modelling to examine a predefined composite clinical–serological profile derived from the existing data. This composite endpoint served exclusively as an exploratory analytical target for investigating within-cohort profile recoverability and was not intended to redefine PANS or PANDAS or establish a validated diagnostic category.

Two analytical settings were evaluated. The first used a complete-information feature set including serological, temporal, onset, symptom-domain, demographic, developmental, systemic, and infection-history variables. The second used a restricted-feature setting in which the most endpoint-proximal variables were removed before model refitting. This two-step approach was intended to distinguish internal reconstruction of the predefined profile from residual clinical signal after removal of its most direct components.

No formal sample-size calculation was performed because the analysis was secondary and exploratory. The available sample was determined by the number of participants retained with analyzable baseline data.

### 2.2. Source Cohort and Baseline Assessment

The source cohort was a naturalistic, clinically referred observational cohort comprising children and adolescents evaluated for neuropsychiatric symptoms in the context of caregiver- or record-supported history suggestive of previous streptococcal exposure. Participants were recruited over a 12-month period (2024–2025) through routine inpatient and outpatient child and adolescent psychiatric care pathways in Romania. The cohort was not population-based, and recruitment did not require a confirmed PANS/PANDAS diagnosis or elevated ASO status.

Previous infectious episodes were documented as part of the routine clinical anamnesis using caregiver-provided history together with available medical information, including prior medical consultations, recorded diagnoses, laboratory investigations, and treatment history when available. When original medical documentation was unavailable, the reported episode was interpreted in the context of a clinically compatible previous infectious presentation rather than on the basis of an isolated retrospective statement. For pharyngotonsillar episodes, clinically compatible features included sudden-onset sore throat or odynophagia, fever, anterior cervical lymphadenopathy, palatal petechiae, pharyngeal or tonsillar erythema, tonsillar hypertrophy with or without exudate, or a scarlatiniform rash, particularly in the absence of prominent viral features such as cough, rhinorrhea, hoarseness, oral ulcers, or conjunctivitis [9]. These features were used to support the clinical plausibility of previous streptococcal exposure and were not considered equivalent to microbiological confirmation of acute group A streptococcal infection. Microbiological confirmation was not systematically available for all historical episodes, and the available analytical dataset did not include a standardized classification of infection-history evidence according to microbiological confirmation, medical documentation, or clinical suspicion. The resulting infectious-history variable therefore reflected clinically supported historical exposure documented within the naturalistic psychiatric-care setting of the source cohort.

Participants were considered for inclusion if they presented with neuropsychiatric symptoms and if caregiver report or available medical records indicated a history suggestive of previous streptococcal exposure. This eligibility approach was chosen to reflect routine child and adolescent psychiatric practice rather than population-based screening, and to characterize the breadth of psychiatric, behavioral, developmental, somatic, and infection-related features observed in children evaluated for neuropsychiatric symptoms in the context of suspected previous streptococcal exposure. Baseline assessment included demographic characteristics, clinical history, infection-related variables, ASO values, PANS-31 item-level and total scores, selected developmental and systemic variables, treatment-related information, and EEG descriptors.

A total of 154 children and adolescents were screened through the source clinical recruitment pathway. Of these, 58 were not retained in the final analytical cohort: 24 had major neurological, developmental, organic, or other medical conditions considered likely to substantially confound interpretation of the neuropsychiatric presentation; 18 had incomplete core assessment data; and 16 withdrew or did not complete the assessment process. The remaining 96 participants constituted the analytical cohort used in the previously published observational study [16] and retained for the present secondary modelling analysis. The original recruitment and exclusion process, including a participant flow diagram, was reported in the source-cohort publication [16], while the corresponding numerical breakdown is also provided in Supplementary Table S1.

Following clinical recruitment and eligibility assessment, the retained cohort was stratified analytically according to ASO status (ASO > 200 IU/mL, *n* = 50; ASO ≤ 200 IU/mL, n = 46), consistent with the previously published observational study [16]. The two serological strata were stored in separate source spreadsheets, which were subsequently harmonized and merged into a single analytical dataset for the present modelling analysis.

No experimental intervention was assigned as part of the present modelling study. Information on previous or ongoing treatments, including penicillin exposure and psychotropic or psychotherapeutic treatment response, was recorded as baseline clinical information when available and was handled as observational data.

### 2.3. Variables Collected in the Source Cohort

Demographic and contextual variables included age (years), sex (male/female), residential environment (urban/rural), family placement (living with family/foster care), and assessment setting (hospital-based/ambulatory care). These categorical characteristics were recorded using predefined binary codes rather than derived through statistical dimensionality-reduction procedures. Their original coding and analytical representation are detailed in Supplementary Table S8. Infection-related and serological variables included time since the last reported streptococcal infection, recurrent infections, continuous ASO value, ASO category, previous ASO-related testing when available, penicillin treatment, non-streptococcal bacterial infections, viral infections, and history compatible with rheumatic fever, chorea, or endocarditis. Abrupt onset or abrupt clinical worsening was recorded from caregiver report and available clinical documentation. More detailed etiological information was retained where available and is summarized by ASO stratum in Supplementary Table S3A. Because individual infectious categories were sparsely represented, non-streptococcal bacterial and viral infection histories were encoded as binary presence/absence variables for supervised modelling.

Clinical-history variables included psychological trauma history, addiction-related history, previous psychiatric record, and the main psychiatric diagnosis recorded at assessment. Age-appropriate Child Symptom Inventory and Adolescent Symptom Inventory domains were available for broader psychometric characterization, including obsessive compulsive, tic-related, anxiety, affective, eating-related, conduct-related, psychotic, pervasive, enuresis, and addiction-related symptom domains.

PANS-related symptom burden was recorded using the PANS-31 total score and individual PANS-31 item scores. Item-level variables covered obsessive compulsive symptoms, restrictive eating or fluid refusal, binge eating, anxiety and mood symptoms, behavioral disruption, irritability or aggression, attention and school-related decline, cognitive or memory symptoms, somatic complaints, urinary symptoms, sensory hypersensitivity, psychotic symptoms, and motor or vocal tics.

Neurodevelopmental, systemic, and vulnerability-related variables included IQ category, allergic or atopic background, prematurity, in vitro fertilization history, functional digestive symptoms, autoimmune pathology, and response to psychotropic treatment or psychotherapy when relevant. Neurophysiological variables included global EEG status and specific identified EEG descriptors, such as posterior alpha rhythm characteristics, theta activity, spike-wave complexes, and nonspecific EEG changes.

Before analysis, equivalent fields from the two source sheets were aligned through targeted column-name standardization. Harmonization included clinical diagnosis fields, eating-related variables, sensory symptoms, motor tics, school performance decline, cognitive or memory deficit, and non-streptococcal bacterial infection variables.

### 2.4. Derived Variables and Operational Endpoint

ASO was represented in two ways. First, the continuous ASO value was retained as ASO_value, allowing serological intensity to be used without thresholding. Second, elevated ASO status was derived as ASO > 200 IU/mL and encoded as ASO_binary, reflecting the serological stratification used in the source dataset. The >200 IU/mL threshold was retained from the original observational cohort, reflecting the laboratory reference boundary used for latex-based ASO testing in the source Romanian clinical settings, as previously documented. This threshold was applied exclusively for operational serological stratification and should not be interpreted as a universally applicable, age-adjusted pediatric upper limit of normal or as an independent diagnostic criterion for PANDAS. In this manuscript, the standard English abbreviation ASO is used for antistreptolysin O, whereas computational variable names retain the source-dataset convention, where ASLO was the original local label.

PANS-31 total score was retained as the principal dimensional measure of neuropsychiatric symptom burden. The scale comprises 31 items rated from 0 (none) to 4 (extreme), yielding a theoretical total-score range of 0–124; higher scores reflect a greater number and/or severity of symptoms. The available psychometric validation did not establish a diagnostic or severity cutoff for the total score [11]. For construction of the present operational endpoint, a PANS-31 total score > 36 was used as a pragmatic higher-burden separator. The >36 threshold was clinically informed by preliminary interviews and pilot administrations of the PANS-31 conducted before formal recruitment into the source cohort, as previously documented [16]. These preliminary assessments contributed to the selection of an operational separator intended to distinguish more clearly expressed multidimensional neuropsychiatric symptom burden from lower-range, potentially nonspecific psychiatric presentations. The threshold was retained before formal statistical analysis and was not retrospectively optimized using the analytical cohort. The choice was empirical and methodological rather than diagnostic. In the naturalistic psychiatric setting of the source cohort, preliminary clinical assessments suggested that lower-range PANS-31 scores could overlap with nonspecific psychiatric, behavioral, anxiety-related, somatic, or developmental manifestations. The >36 threshold was therefore adopted to identify a subgroup with more clearly expressed multidimensional neuropsychiatric symptom burden while retaining an informative analytical subgroup, given that very high PANS-31 scores were not broadly represented in the cohort. Post hoc descriptive sensitivity analyses showed that the observed ASO–symptom-burden pattern was not specific to the exact >36 cut point, but remained broadly stable around this prespecified score. This finding may inform future studies aimed at defining clinically interpretable and externally reproducible PANS-31 score strata. Because these analyses were intended to assess robustness rather than identify an optimal threshold, the prespecified >36 separator was retained unchanged for the present modelling analysis. It should therefore be interpreted as an internal operational threshold and not as a validated diagnostic or severity cutoff.

The operational endpoint required the simultaneous presence of four baseline conditions:

1. PANS-31 total score > 36;
2. ASO > 200 IU/mL;
3. Abrupt symptom onset or abrupt clinical worsening;
4. Less than six months since the last reported streptococcal infection.

The four components were combined because they represent complementary dimensions of the clinical pattern examined in the source cohort rather than interchangeable manifestations of the same construct. PANS-31 captured multidimensional neuropsychiatric symptom burden; elevated ASO represented the predefined serological stratum compatible with previous streptococcal exposure; abrupt onset or abrupt worsening captured the clinically relevant temporal pattern of neuropsychiatric change; and infection recency represented temporal proximity between the reported infectious episode and the index neuropsychiatric assessment. In the previously reported descriptive phase of this cohort, higher ASO values, shorter reported infection intervals, abrupt onset or worsening, and greater PANS-31 symptom burden showed a coherent pattern of association [16]. In particular, participants with ASO > 200 IU/mL had a median interval of 3 months [IQR: 2–5] since the last reported streptococcal infection, compared with 12 months [IQR: 12–12] among participants with ASO ≤ 200 IU/mL (with intervals exceeding 12 months recoded as 12 for analytical purposes) [16]. The <6-month criterion was therefore used as a pragmatic operational representation of the recent-exposure pattern identified in the source cohort rather than as a validated diagnostic cutoff.

In the computational dataset, the temporal criterion corresponded to last_strep_infection_episode_months < 6. The infection-recency variable was derived from caregiver-reported history, supplemented by available medical documentation and clinical context, as described in Section 2.2. Because historical infectious episodes were not uniformly microbiologically confirmed, this variable represented the reported interval since an episode clinically suggestive of streptococcal infection rather than the verified timing of a microbiologically confirmed group A streptococcal infection. Abrupt symptom onset or clinical worsening was defined as the sudden emergence of new neuropsychiatric manifestations or a clear exacerbation of pre-existing symptoms occurring approximately within the week preceding the index psychiatric assessment, as established through caregiver-reported history and available clinical documentation. This retrospective interval referred to the timing of the clinical change relative to assessment, rather than to a measured duration of symptom development. The variable was recorded independently of infection timing and was not a universal eligibility criterion for the source cohort; its coding and role in the operational endpoint are detailed in Supplementary Table S8. Participants meeting all four criteria were coded as endpoint-positive; all remaining participants were coded as endpoint-negative.

The endpoint was intended to represent a rule-defined streptococcal-exposure-associated clinical–serological profile within the cohort. It was not treated as physician-confirmed PANDAS and was not used as evidence of streptococcal causality. Endpoint positivity was likewise not considered equivalent to a clinical diagnosis of PANS. PANS-31 was used as a dimensional measure of multidomain neuropsychiatric symptom burden, while the composite endpoint served only as an operational target for the present secondary modelling analysis. All endpoint components were derived from baseline clinical, psychometric, serological, and caregiver- or record-informed data.

Therefore, the modelling task was cross-sectional, and no prospective prognostic time horizon was defined. Blinded outcome adjudication was not performed because the endpoint was algorithmically derived from recorded baseline variables.

### 2.5. Exploratory Statistical Characterization and Predictor Rationale

Before supervised modelling, descriptive statistical analyses were used to characterize the cohort and support the clinical rationale for the modelling framework. These analyses were intended to describe the structure of the dataset and they further helped guide the interpretation of the modelling results.

Endpoint-positive and endpoint-negative participants were characterized descriptively using median [interquartile range] for continuous variables and n (%) for categorical variables. No inferential hypothesis testing was performed for this subgroup description because several variables were structurally related to endpoint construction and the purpose was descriptive clinical characterization rather than independent association testing.

Associations involving numerical variables, including PANS-31 total score, continuous ASO value, and infection-recency measures, were examined using Spearman’s rank correlation coefficients with two-sided *p*-values. Continuous variables were compared across binary categorical strata using Mann–Whitney U tests. For categorical variables with three ordered levels, omnibus comparisons were performed using Kruskal–Wallis tests, followed, where appropriate, by pairwise Mann–Whitney U tests with Bonferroni adjustment for multiple comparisons. One-way ANOVA results generated during the initial exploratory analysis were regarded only as supplementary parametric consistency checks and were not used as the primary inferential basis.

Age-appropriate Child Symptom Inventory and Adolescent Symptom Inventory domains were examined during broader cohort characterization to assess convergence between PANS-31 symptom burden and related psychiatric symptom domains.

These descriptive analyses were not used as an automatic variable selection procedure. Instead, candidate predictors were selected from the initial descriptive cohort findings, by availability in the harmonized dataset, interpretability, and consistency with domains emphasized in the available PANS/PANDAS clinical literature and guidance. Particular consideration was given to variables with clinical and statistical plausibility in relation to infection timing, abrupt onset or abrupt worsening, developmental vulnerability, systemic context, and multidimensional neuropsychiatric symptom burden.

### 2.6. Candidate Predictors

The initial candidate predictor pool was selected from variables available in the harmonized dataset. Candidate variables represented demographic and contextual information, infection history, ASO intensity, onset pattern, selected PANS-31 item-level symptom indicators, developmental vulnerability, systemic features, and immune-related clinical background.

Selected PANS-31 item-level variables were transformed into binary high-intensity indicators using a score > 2 on the original 0–4 item scale. These indicators represented symptom domains emphasized in PANS/PANDAS-oriented clinical descriptions, including obsessive compulsive symptoms, eating or intake disturbance, tic symptoms, and urinary symptoms.

PANS-31 total score, PANS-31 > 36, binary ASO status, and ASO category were not entered as ordinary predictors. These variables were either used directly for endpoint construction or were redundant with endpoint components. Continuous ASO value, infection recency, and abrupt onset were retained only in the full-feature setting and excluded from the restricted-feature sensitivity setting.

No automated feature selection algorithms, recursive feature-elimination procedures, or high-dimensional feature-engineering strategies were applied. Full variable handling is summarized in Table 1 and Table S8.

### 2.7. Final Modelling Feature Sets and Variables

Two final modelling matrices were constructed after harmonization and clinical pre-selection.

The full-feature matrix included 96 participants and 27 retained predictors: age, sex, residential environment, family context, care setting, time since the last reported streptococcal infection, recurrent infections, continuous ASO value, binary obsessions, binary compulsions, binary hoarding, binary food refusal, binary binge eating, binary fluid refusal, binary motor tics, binary vocal tics, binary enuresis, penicillin exposure, abrupt symptom onset, allergic or atopic background, prematurity, in vitro fertilization history, functional digestive symptoms, non-streptococcal bacterial infections, viral infections, autoimmune pathology, and history compatible with rheumatic fever, chorea, or endocarditis.

The restricted-feature matrix included the same participants and 24 predictors. It excluded three variables from the full-feature set: continuous ASO value, time since the last reported streptococcal infection, and abrupt onset. This restricted matrix was used to evaluate whether symptom-domain, developmental, systemic, infection-history, and contextual variables retained discriminative information after removal of the most direct serological, temporal, and onset-related predictors. Because selected PANS-31 item-level indicators were retained, the restricted-feature analysis was not fully independent of the symptom-burden component of the operational endpoint and should therefore be interpreted as a sensitivity analysis rather than as an endpoint-independent model.

Variables not retained in the final modelling matrices included ASI/CSI domains, EEG descriptors, IQ category, diagnosis codes, psychiatric record, trauma history, addiction-related history, and treatment-response variables. These variables were used for source-cohort characterization or exploratory analyses but were not entered into the supervised models, in order to reduce dimensionality and preserve interpretability in a small cohort.

Table 1 summarizes variable handling and the two analytical feature settings.

### 2.8. Data Harmonization and Preprocessing

Data were extracted from the two predefined ASO-stratified source sheets and concatenated after harmonization. Non-participant rows, empty rows, and coding-legend rows were removed before analysis. Participant records were retained when the variables required for endpoint construction and modelling were available.

The variable representing time since the last reported streptococcal infection was standardized before analysis. Entries coded as “>12” months were recoded as 12 and represented as integer months. Continuous ASO values were retained in IU/mL.

For the supervised modelling workflow, categorical text labels were numerically encoded, while numerical variables retained their original representation except where specific transformations were required. In particular, diagnostic identifiers recorded for allergic/atopic and autoimmune conditions were recoded into binary presence/absence indicators (0 = absent; 1 = present), as these identifiers represented diagnostic categories rather than ordinal measurements or severity scores. The definitions, coding conventions, and modelling status of the collected variables are detailed in Supplementary Table S8. During data harmonization, two initially blank binary entries in a single participant record were verified against the original source worksheet and confirmed to represent absence of the corresponding features; both were therefore coded as 0. No unresolved missing predictor values remained in the final analysis-ready dataset, and no statistical imputation was required.

Logistic Regression was trained on standardized features using StandardScaler, whereas Random Forest and XGBoost were trained on unscaled encoded features. Standardization parameters for Logistic Regression were fitted on the training partition and then applied to the held-out test partition. The same train–test split was used for the full-feature and restricted-feature analyses to make internal comparisons more direct. Preprocessing was not tailored by sex, age, residential environment, care setting, or other sociodemographic subgroups.

### 2.9. Supervised Modelling and Internal Evaluation

The supervised endpoint was the operational clinical–serological profile defined in Section 2.4. Three model families were evaluated: Logistic Regression, Random Forest, and XGBoost. Each model was trained in the full-feature and restricted-feature settings described in Section 2.7.

Hyperparameters were tuned using GridSearchCV, with ROC-AUC as the optimization metric and stratified 5-fold cross-validation. Logistic Regression and Random Forest used class-weight balancing, whereas XGBoost used scale_pos_weight to account for class imbalance. No oversampling, SMOTE, or synthetic minority-sample generation was applied. Given the limited cohort size and the 50:17 negative-to-positive class ratio in the training set, weighting-based approaches were preferred to avoid introducing synthetic observations that could increase the risk of optimistic or unstable internal performance estimates. The retained hyperparameters for each model family and feature setting are reported in Table 2.

Following cohort harmonization and variable preparation, the 96 participants were divided into a training set (70%, *n* = 67; 17 endpoint-positive and 50 endpoint-negative) and a held-out test set (30%, *n* = 29; 7 endpoint-positive and 22 endpoint-negative), using stratified random sampling (random_state = 42) to preserve the endpoint class distribution.

Model fitting and hyperparameter optimization were performed exclusively within the training set, using stratified five-fold cross-validation for model selection. The held-out test set was excluded from model fitting and hyperparameter tuning and was used for final internal performance evaluation. For Logistic Regression, StandardScaler parameters were estimated from the training set and subsequently applied to the test set without refitting.

The same training–test partition was maintained across all three model families and both feature settings. Restricted-feature models were independently refitted using the corresponding training data after exclusion of the three endpoint-proximal predictors. Importantly, the held-out test set represented a separate participant partition from the same clinical cohort, rather than an external validation dataset. Accordingly, the reported performance measures were interpreted as internal estimates. Nested cross-validation was not performed; therefore, cross-validated ROC-AUC values were regarded as internal model selection estimates rather than unbiased external performance estimates.

The main manuscript reports ROC-AUC, sensitivity, and specificity. Complete metric output, including positive predictive value, negative predictive value, confusion-matrix counts, and descriptive 95% confidence intervals for held-out ROC-AUC, sensitivity, and specificity, is provided in Supplementary Table S12. Sensitivity and specificity confidence intervals were calculated as exact binomial intervals. Held-out ROC-AUC confidence intervals were reported as approximate descriptive intervals because the held-out test set was small.

All performance estimates were interpreted as internal discrimination and profile reconstruction within the available cohort.

### 2.10. SHAP-Based Interpretation Methods

Model attribution was examined using SHAP values. LinearExplainer was used for Logistic Regression, whereas TreeExplainer was used for Random Forest and XGBoost. For each fitted model, mean absolute SHAP values were computed to rank predictors by global contribution to model output.

SHAP-based interpretation was performed separately for full-feature and restricted-feature models. In the full-feature setting, SHAP analysis was used to inspect how serological, temporal, onset-related, symptom-domain, and contextual variables contributed to internal reconstruction of the operational profile. In the restricted-feature setting, SHAP analysis was used to examine how residual symptom-domain, developmental, systemic, infection-history, and contextual variables behaved after removal of continuous ASO value, infection recency, and abrupt onset.

SHAP values were used as a model-behaviour inspection measure. They were not interpreted as evidence of biological mechanism, disease specificity, causality, or patient-level diagnostic explanation. Convergence between SHAP-ranked predictors and clinically expected features was interpreted as support for internal profile coherence within the cohort, not as proof of PANS/PANDAS.

### 2.11. Software Environment and Computational Workflow

The computational analyses were performed in Python 3.8, using pandas (version 1.5), NumPy (1.24), SciPy (1.10), scikit-learn (1.2), XGBoost (2.1), SHAP (0.44), and Matplotlib (3.7), as reported for the original analytical environment.

The computational workflow comprised data harmonization and variable preparation, construction of the operational composite endpoint, and numerical encoding of the retained predictors, followed by stratified training–test partitioning (test_size = 0.30, random_state = 42). Logistic Regression used StandardScaler fitted on the training partition, whereas Random Forest and XGBoost were trained without feature scaling. Model hyperparameters were optimized using GridSearchCV with stratified five-fold cross-validation and ROC-AUC as the selection metric, followed by refitting on the training partition and evaluation on the held-out test set.

Model interpretation was performed using SHAP, with LinearExplainer for Logistic Regression and TreeExplainer for the tree-based models. The training partition served as the background dataset for SHAP attribution. The corresponding model configurations and retained hyperparameters are reported in Table 2, while the analytical procedures and extended results are documented in the Supplementary Materials. The original Python analytical workflow, including the exploratory analyses, is provided as an additional Supplementary HTML File.

## 3. Results

### 3.1. Cohort Structure and Operational Endpoint

The final analytical dataset included 96 children and adolescents with analyzable baseline data. The cohort was derived from two predefined ASO-stratified source sheets: 50 participants with ASO > 200 IU/mL and 46 participants with ASO ≤ 200 IU/mL. Thus, 52.1% of the cohort belonged to the elevated-ASO stratum and 47.9% to the non-elevated-ASO stratum.

A PANS-31 total score > 36 was present in 34 participants. All participants with PANS-31 > 36 belonged to the elevated-ASO stratum. This strong alignment reflects the enriched and ASO-stratified structure of the source cohort and is important for interpreting subsequent model discrimination. This enrichment likely increased the separability of the operational profile within the present dataset and limits direct extrapolation of the observed discrimination to unselected pediatric psychiatric or community populations.

The supervised endpoint was the operational streptococcal-exposure-associated clinical–serological profile defined in Section 2.4. This stricter composite profile was present in 24 participants and absent in 72 participants. After stratified 70/30 splitting, the training set included 67 observations and the held-out test set included 29 observations. Endpoint distribution was preserved across partitions, with 17 endpoint-positive and 50 endpoint-negative observations in the training set and 7 endpoint-positive and 22 endpoint-negative observations in the held-out test set. Cohort structure, endpoint distribution, and train–test partitioning are summarized in Table 3.

This distribution shows that the endpoint-positive subgroup represented a clinically enriched minority of the analytical cohort rather than a separate diagnostic group. The preserved endpoint distribution across training and held-out test partitions provided the basis for the internal modelling comparisons reported below.

### 3.2. Clinical–Serological Context of the Modelling Endpoint

The 24 endpoint-positive participants constituted a clinically enriched subgroup within the analytical cohort. Their median age was 13 years [IQR: 12–14], and 17/24 (70.8%) were male. As required by endpoint construction, all had ASO > 200 IU/mL, PANS-31 total score > 36, abrupt symptom onset or abrupt clinical worsening, and a reported streptococcal infection interval of less than six months. Within this subgroup, median ASO was 674 IU/mL [IQR: 586–784], median time since the last reported streptococcal infection was 3 months [IQR: 2–3], and median PANS-31 total score was 44 [IQR: 40–50]. Beyond the defining endpoint components, 8/24 (33.3%) had a history of recurrent infections, 8/24 (33.3%) had a history of prematurity, 6/24 (25.0%) had an in vitro fertilization history, and 17/24 (70.8%) had one or more recorded functional digestive symptoms. Among the prespecified high-intensity PANS-31 item indicators (item score > 2), motor tics were present in 14/24 (58.3%), food refusal in 11/24 (45.8%), enuresis in 10/24 (41.7%), obsessions in 7/24 (29.2%), compulsions in 7/24 (29.2%), binge eating in 7/24 (29.2%), and vocal tics in 5/24 (20.8%). A more complete descriptive characterization of endpoint-positive and endpoint-negative participants is provided in Supplementary Table S9, Panel B.

The ASO-stratified descriptive profile showed separation across serological, temporal, and symptom-burden variables. Participants in the elevated-ASO stratum had higher ASO values, shorter time since the last reported streptococcal infection, and higher PANS-31 total scores. Median ASO value was 648 IU/mL [IQR: 527–764] in the ASO > 200 IU/mL group and 116 IU/mL [IQR: 74–160] in the ASO ≤ 200 IU/mL group. The median interval since the last reported streptococcal infection was 3 months [IQR: 2–5] in the elevated-ASO group and 12 months [IQR: 12–12] in the non-elevated-ASO group. Median PANS-31 total score was 40 [IQR: 34–50] in the elevated-ASO group and 22 [IQR: 18–25] in the non-elevated-ASO group.

A similar pattern was seen when the cohort was stratified by PANS-31 burden. Participants with PANS-31 > 36 had higher ASO values, shorter time since the last reported streptococcal infection, and higher PANS-31 total scores than participants with PANS-31 ≤ 36. Median ASO value was 674 IU/mL [IQR: 549–788] in the PANS-31 > 36 subgroup and 150 IU/mL [IQR: 90–283] in the PANS-31 ≤ 36 subgroup. Median time since the last reported streptococcal infection was 3 months [IQR: 2–4] in the PANS-31 > 36 subgroup and 12 months [IQR: 6–12] in the PANS-31 ≤ 36 subgroup. These strata were overlapping but not identical: all participants with PANS-31 > 36 were in the elevated-ASO stratum, whereas 16 participants with ASO > 200 IU/mL had PANS-31 scores ≤ 36.

Several categorical variables also differed across PANS-31 strata. Abrupt onset was present in 27/34 participants (79.4%) with PANS-31 >36 and in 21/62 participants (33.9%) with PANS-31 ≤ 36. Prematurity was recorded in 14/34 participants (41.2%) with PANS-31 > 36 and 7/62 participants (11.3%) with PANS-31 ≤ 36. Functional digestive symptoms were also more frequent in the PANS-31 > 36 subgroup: 18/34 participants (52.9%) had one functional digestive symptom and 7/34 (20.6%) had two or more, compared with 14/62 (22.6%) and 4/62 (6.5%), respectively, in the PANS-31 ≤ 36 subgroup.

Table 4 summarizes the pre-modelling signals from the current ASO/PANS statistical workflow that directly informed the supervised modelling rationale. The PANS-31 convergence with age-appropriate ASI/CSI domains is included only as contextual support for its use as a dimensional symptom-burden measure in the present modelling analysis.

Taken together, these descriptive findings supported the use of a composite operational endpoint rather than a single serological or symptom-based marker. The subsequent supervised analyses therefore examined whether this combined ASO-infection timing–abrupt onset–PANS-31 pattern could be reconstructed internally, and how much of that reconstruction persisted after removal of the most endpoint-proximal predictors.

### 3.3. Full-Feature and Restricted-Feature Internal Modelling of the Operational Profile

In the full-feature complete-information setting, the operational profile was internally recoverable across all three model families. Held-out ROC-AUC values were 0.909 for Logistic Regression, 0.916 for Random Forest, and 0.961 for XGBoost. Cross-validated ROC-AUC values were similarly high, ranging from 0.917 to 0.960. At the default 0.50 threshold, sensitivity/specificity was 0.857/0.864 for Logistic Regression, 0.714/0.818 for Random Forest, and 1.000/0.773 for XGBoost. Because the held-out test set included only 29 participants, including seven endpoint-positive cases, threshold-based metrics were treated as descriptive internal estimates. In addition, 95% confidence intervals for held-out ROC-AUC, sensitivity, and specificity are reported in Supplementary Table S12 and were wide, reinforcing the exploratory nature of the analysis.

After removal of the continuous ASO value, infection recency, and abrupt onset, discrimination decreased across model families. Held-out ROC-AUC values were 0.792 for Logistic Regression, 0.630 for Random Forest, and 0.714 for XGBoost. At the default 0.50 threshold, all three restricted-feature models had sensitivity of 0.429, with specificities of 0.864, 0.909, and 0.818, respectively. This attenuation indicates that the removed endpoint-proximal variables carried a large share of the recoverable signal, while symptom-domain and contextual variables retained weaker, model-dependent information.

Therefore, the full-feature and restricted-feature results answer complementary questions. The full-feature models evaluate whether the predefined ASO–infection timing–abrupt onset–PANS-31 pattern is internally coherent when clinically central information is available. The restricted-feature refit evaluates how much residual structure remains after removing the most direct serological, temporal, and onset-related information. The restricted-feature analysis should therefore be interpreted as a sensitivity analysis of residual signal, not as a separate diagnostic model. Table 5 summarizes internal performance of the full-feature and restricted-feature models.

**Figure 1:**
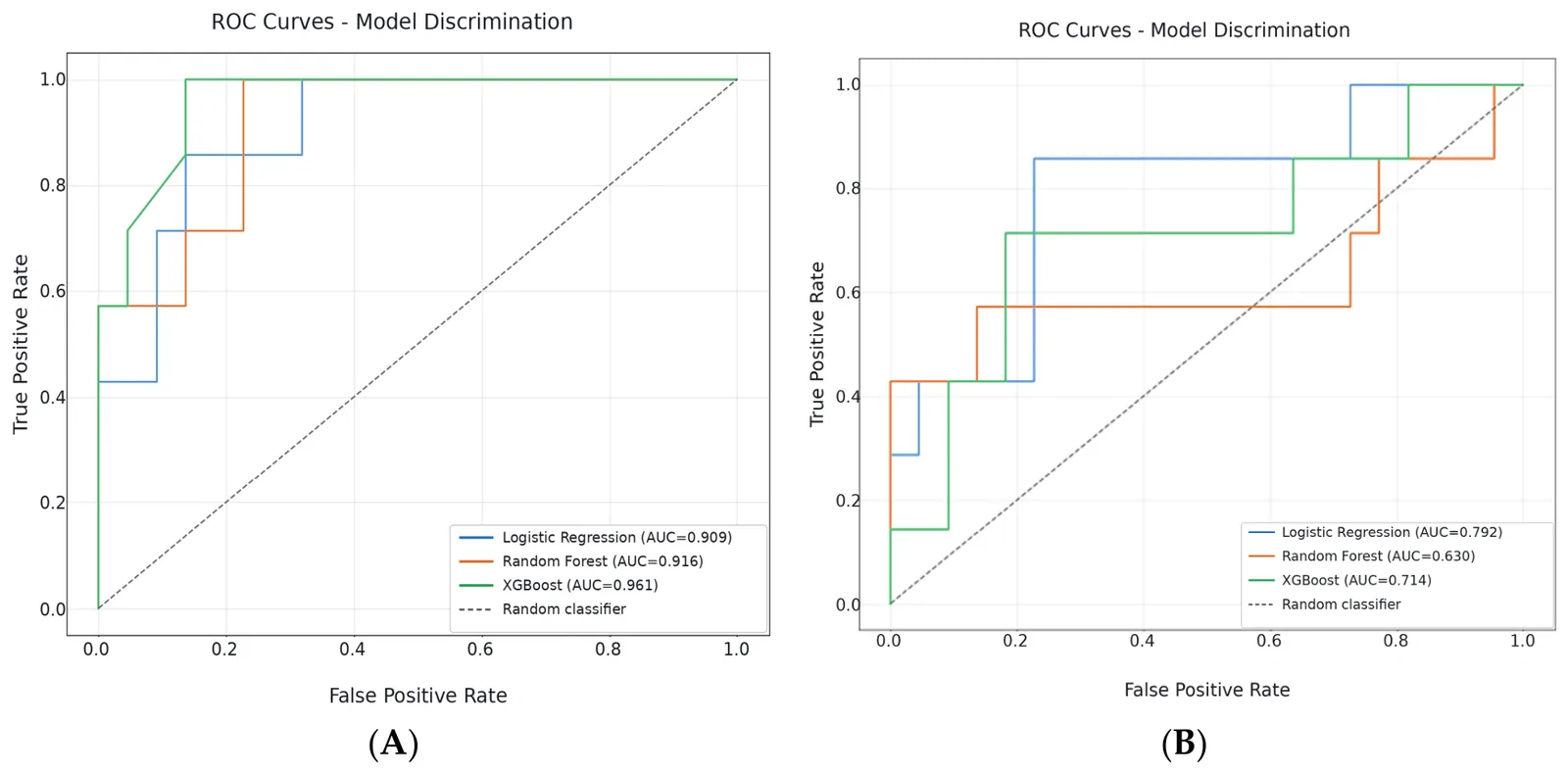
Held-out internal ROC curves for full-feature and restricted-feature models. (**A**) Full-feature models using all 27 retained predictors. (**B**) Restricted-feature models refitted after excluding continuous ASO value, time since the last reported streptococcal infection, and abrupt onset. Curves summarize held-out internal discrimination.

The ROC curves illustrate the same attenuation shown in Table 5: discrimination remained high in the complete-information setting but became more variable after endpoint-proximal variables were removed (Figure 1). Because ROC-AUC summarizes discrimination across possible thresholds but does not show how observations are classified at a specific threshold, confusion matrices were also inspected descriptively at the default 0.50 threshold.

**Figure 2:**
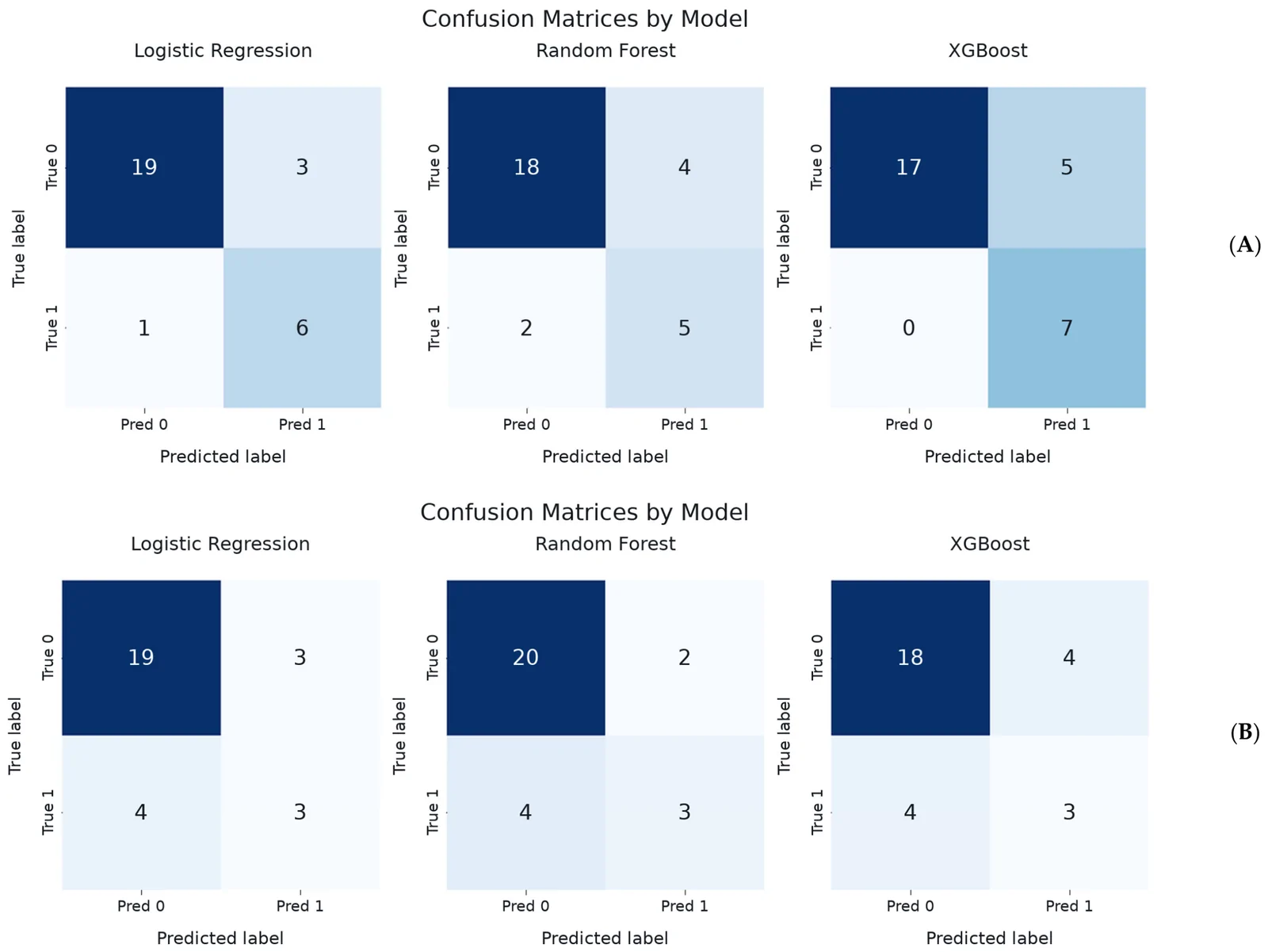
Held-out test-set confusion matrices for full-feature and restricted-feature models. (**A**) Full-feature models. (**B**) Restricted-feature models refitted after excluding continuous ASO value, time since the last reported streptococcal infection, and abrupt onset. Matrices were computed using the default 0.50 classification threshold and are presented as descriptive complements to ROC-AUC. Cell color intensity represents observation counts, with darker blue indicating higher counts and lighter blue indicating lower counts. Numerical values within the cells indicate the corresponding absolute counts.

Confusion matrices at the default 0.50 classification threshold are shown in Figure 2.

To compare cross-validated model selection performance with held-out test performance, ROC-AUC values were then plotted across model families and feature settings.

**Figure 3:**
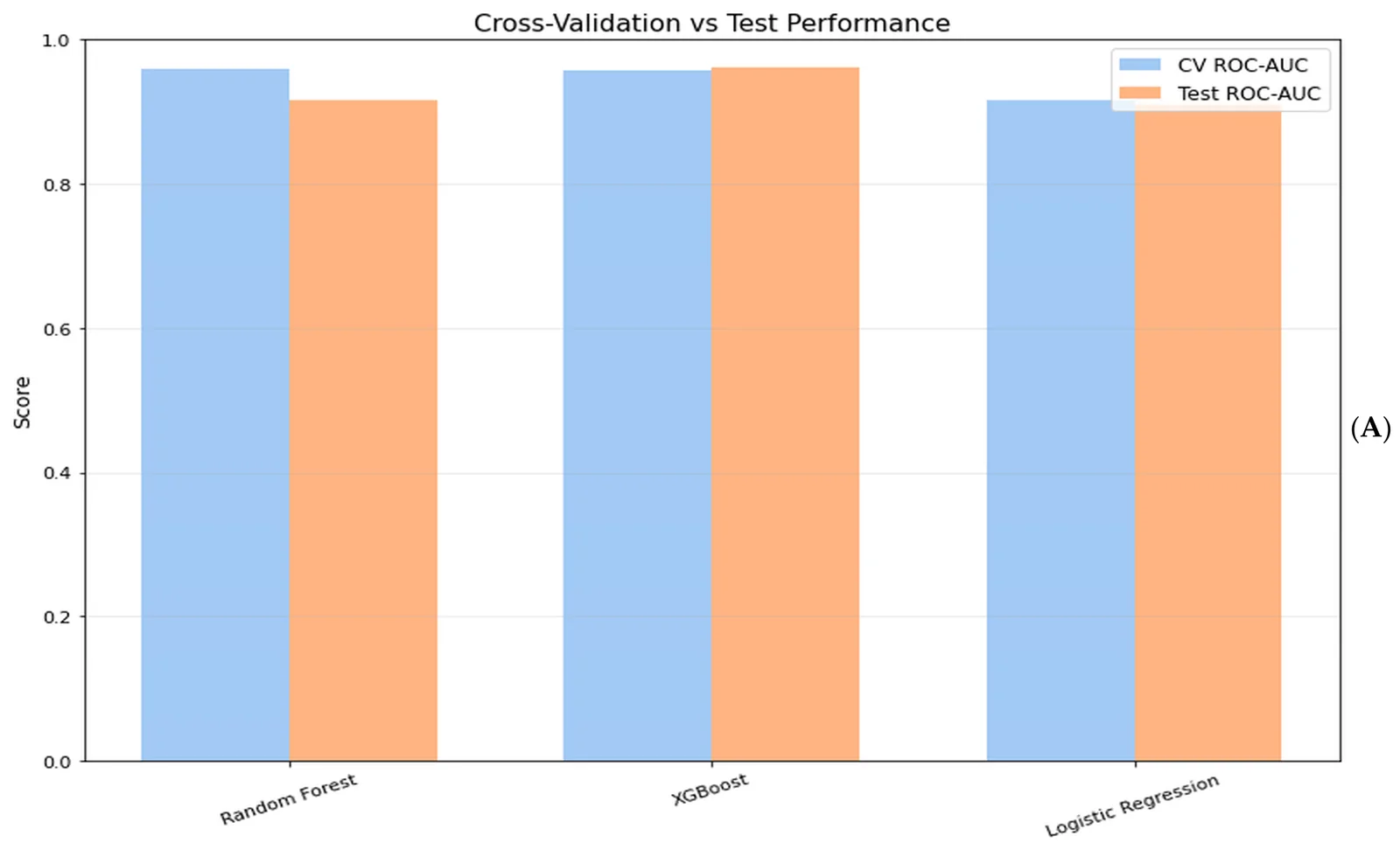

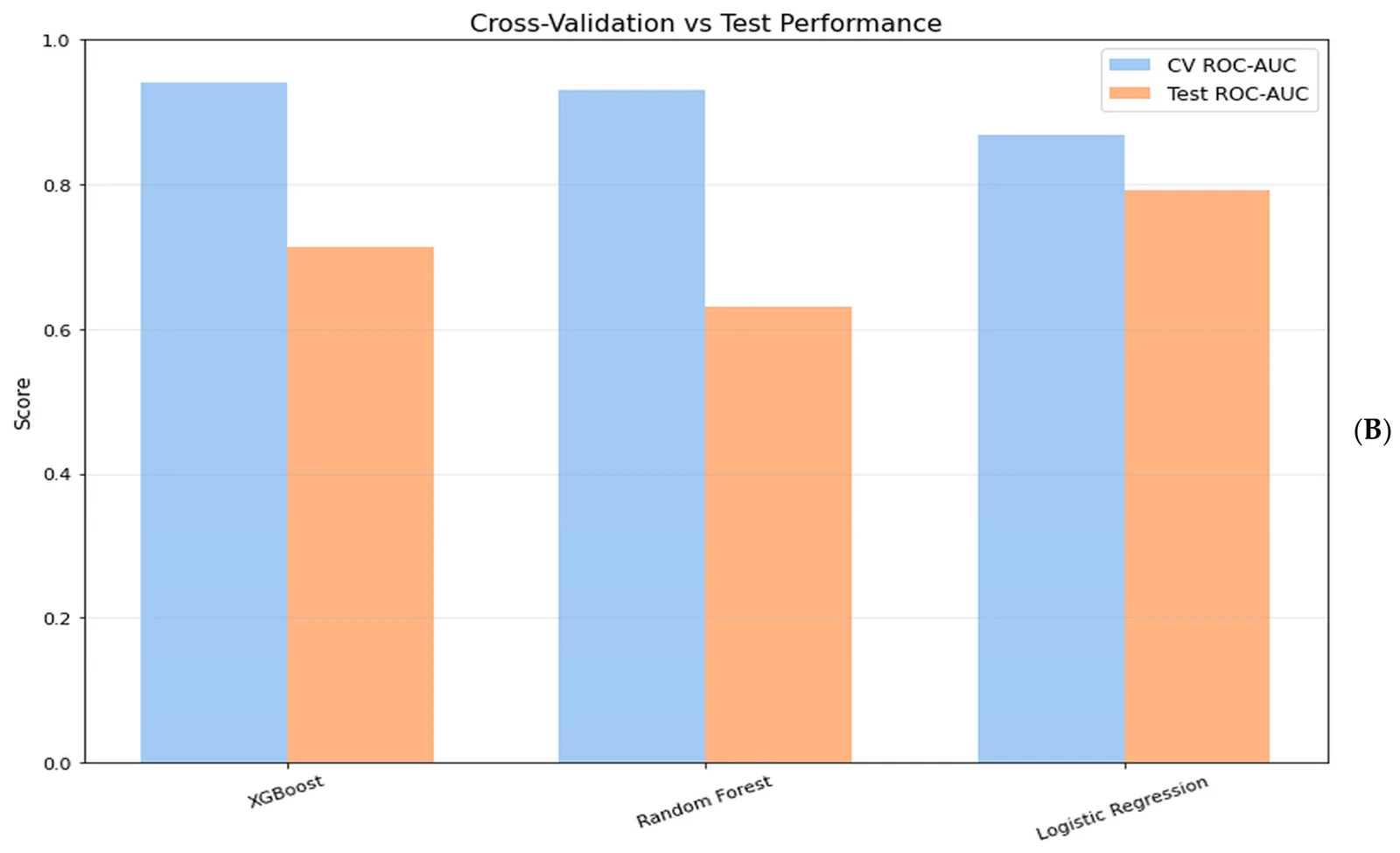
Cross-validated and held-out ROC-AUC values across model families and feature settings. (**A**) Full-feature models. (**B**) Restricted-feature models. The plot compares Logistic Regression, Random Forest, and XGBoost for internal reconstruction of the operational streptococcal-exposure-associated clinical–serological profile.

Cross-validated and held-out ROC-AUC values are compared across model families and feature settings in Figure 3. In the full-feature setting, all three model families showed high internal discrimination. Although XGBoost had the numerically highest held-out ROC-AUC in this setting, this difference should not be interpreted as evidence of algorithmic superiority because the held-out test set was small and contained only seven endpoint-positive cases. In the restricted-feature setting, discrimination decreased across all model families, with Logistic Regression showing the numerically highest held-out ROC-AUC; therefore, the comparison across models is best interpreted as methodological triangulation rather than as a performance ranking.

Overall, the discrimination analyses supported two complementary interpretations. First, the operational profile was strongly recoverable when serological, temporal, onset-related, and symptom-domain variables were jointly available. Second, the restricted-feature attenuation indicated that the removed variables carried much of the recoverable signal, while residual clinical information was weaker and more model-dependent.

### 3.4. SHAP-Based Model Interpretation

SHAP-based interpretation was used to examine how the fitted models reconstructed the operational profile in the two feature settings. In the full-feature models, attribution was dominated by clinically central and endpoint-proximal variables. Across model families, the highest-ranked predictors included time since the last reported streptococcal infection, abrupt onset, continuous ASO value, age, and selected PANS-31 item-level indicators, particularly motor tics, food refusal, binge eating, obsessions, and compulsions.

After removal of continuous ASO value, infection recency, and abrupt onset, SHAP attribution shifted toward residual symptom-domain, age-related, systemic, and contextual variables. In the restricted-feature models, the most informative variables included age and selected PANS-31 item-level indicators such as food refusal, binge eating, motor tics, obsessions, and compulsions, together with selected contextual or systemic features. This shift was consistent with the performance attenuation observed in the restricted-feature models and supports the interpretation that the restricted analysis estimated residual clinical signal rather than reconstructing the serological–temporal–onset components of the endpoint.

**Figure 4:**
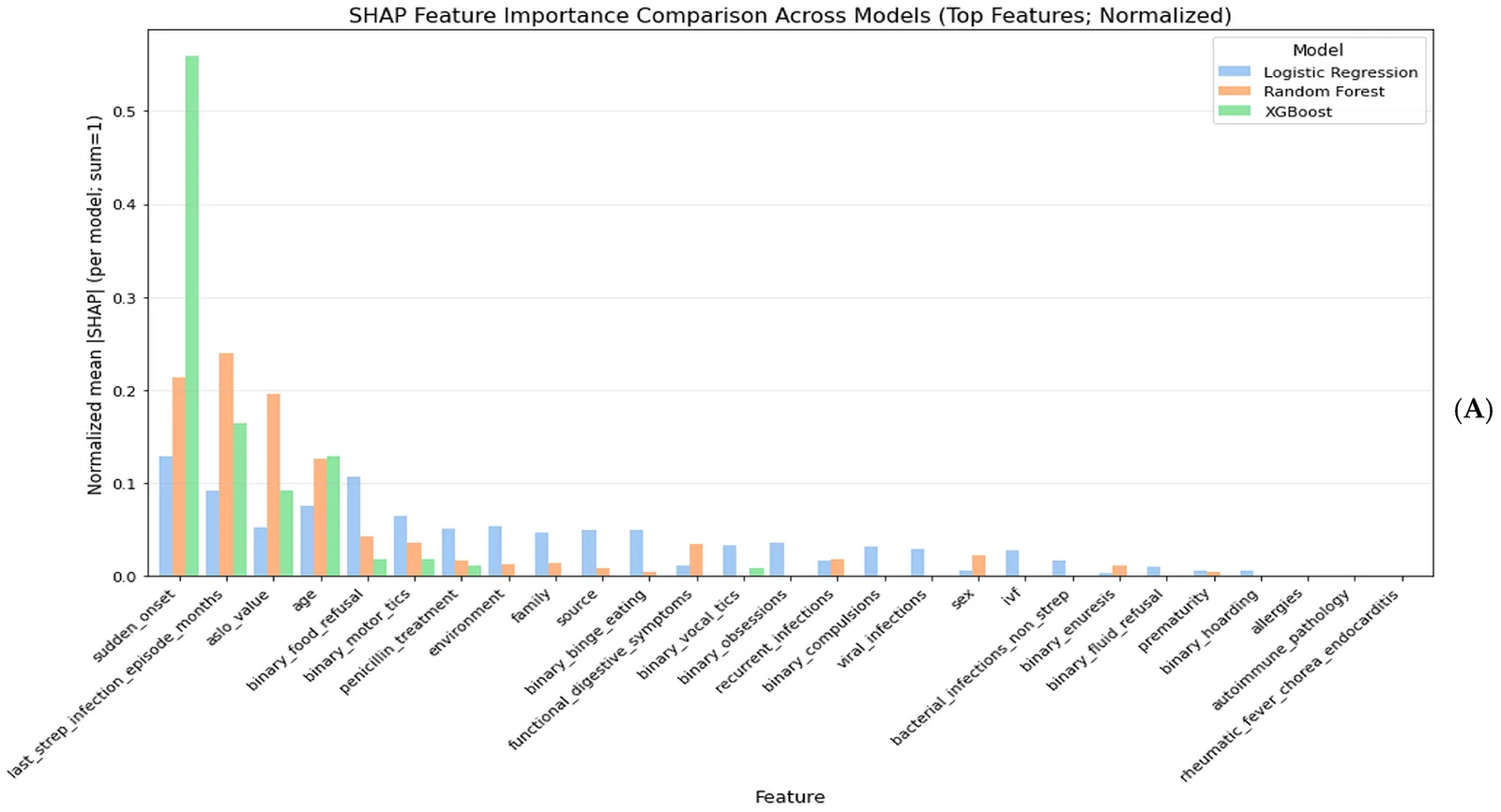

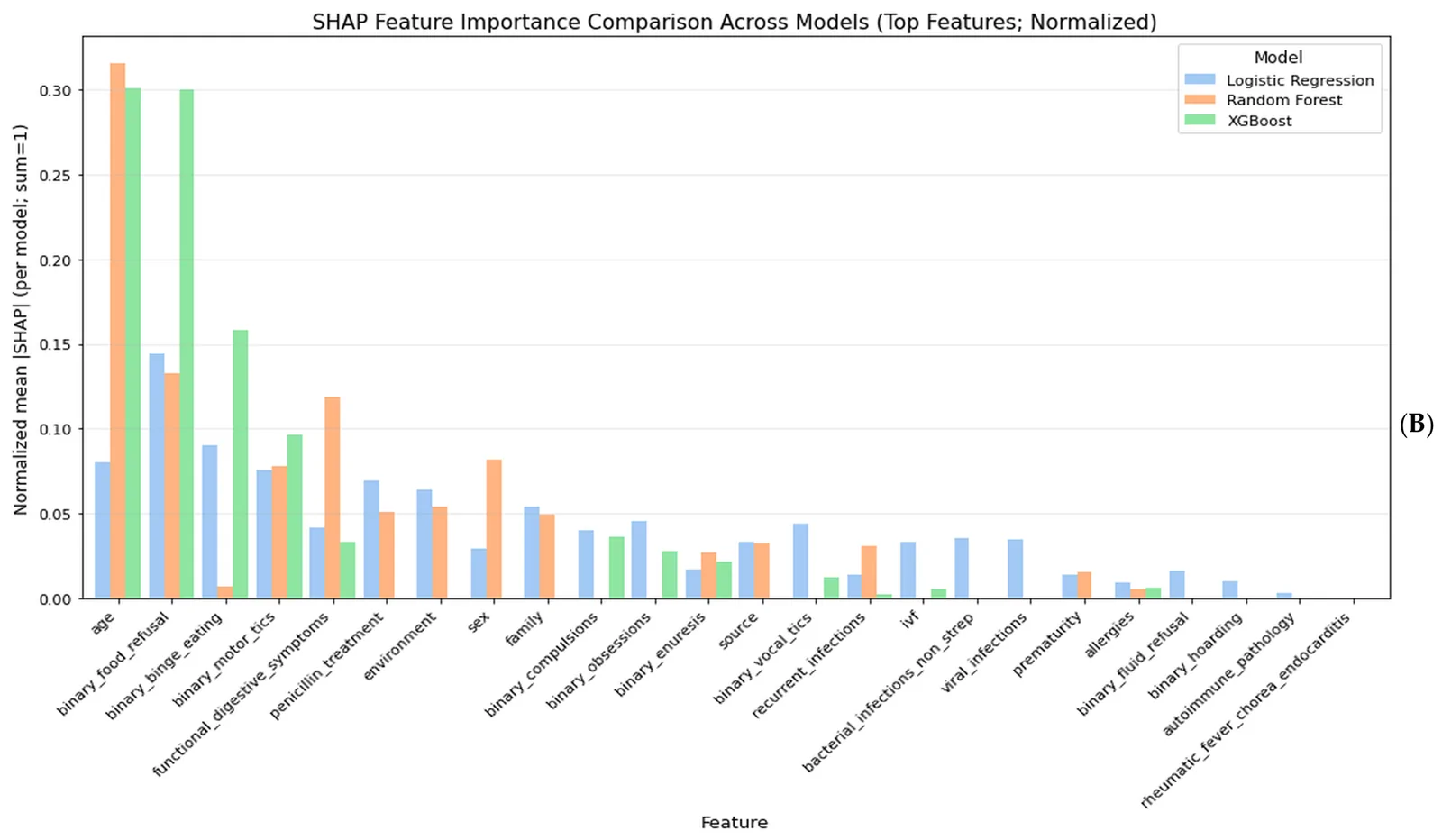
SHAP-based global feature importance in full-feature and restricted-feature models. (**A**) Full-feature models. (**B**) Restricted-feature models. Normalized mean absolute SHAP values are shown for the highest-ranked predictors, with SHAP magnitudes scaled within each model to sum to 1 to facilitate comparison across model families. In the full-feature setting, attribution was dominated by serological, temporal, onset-related, and symptom-domain variables. In the restricted-feature setting, attribution shifted toward residual symptom-domain, age-related, systemic, and contextual variables.

SHAP values were used to characterize model-attribution patterns across the full-feature and restricted-feature settings. The highest-ranked SHAP predictors in each model are summarized in Table 6. To complement this compact summary, global SHAP importance plots were used to compare attribution patterns between full-feature and restricted-feature settings (Figure 4).

**Figure 5:**
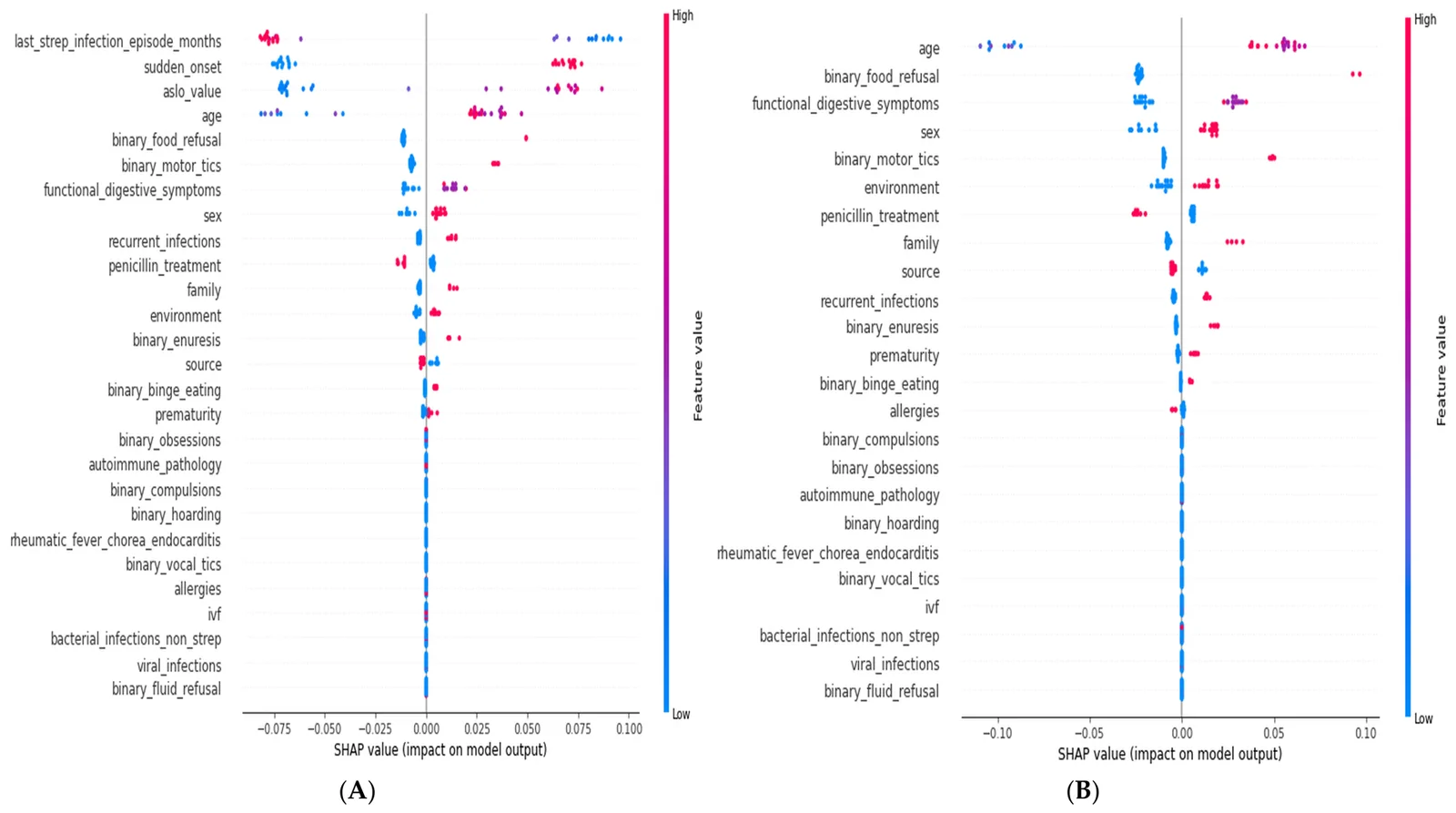
SHAP beeswarm plots for the Random Forest models. (**A**) Full-feature Random Forest model. (**B**) Restricted-feature Random Forest model. The plots show the distribution and direction of SHAP values across observations.

**Figure 6:**
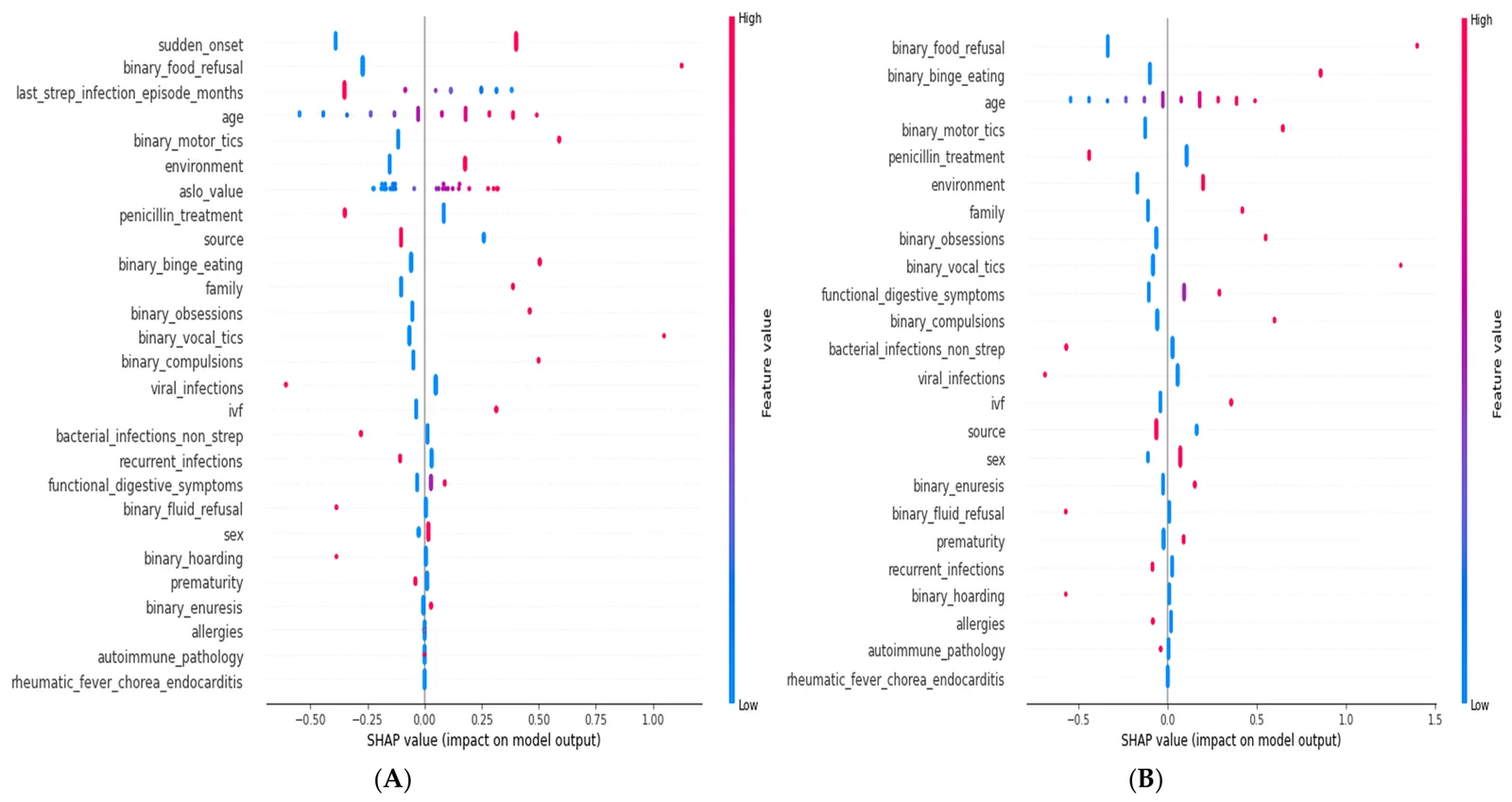
SHAP beeswarm plots for the Logistic Regression models. (**A**) Full-feature Logistic Regression model. (**B**) Restricted-feature Logistic Regression model. Positive and negative SHAP values indicate the direction of contribution to model output.

**Figure 7:**
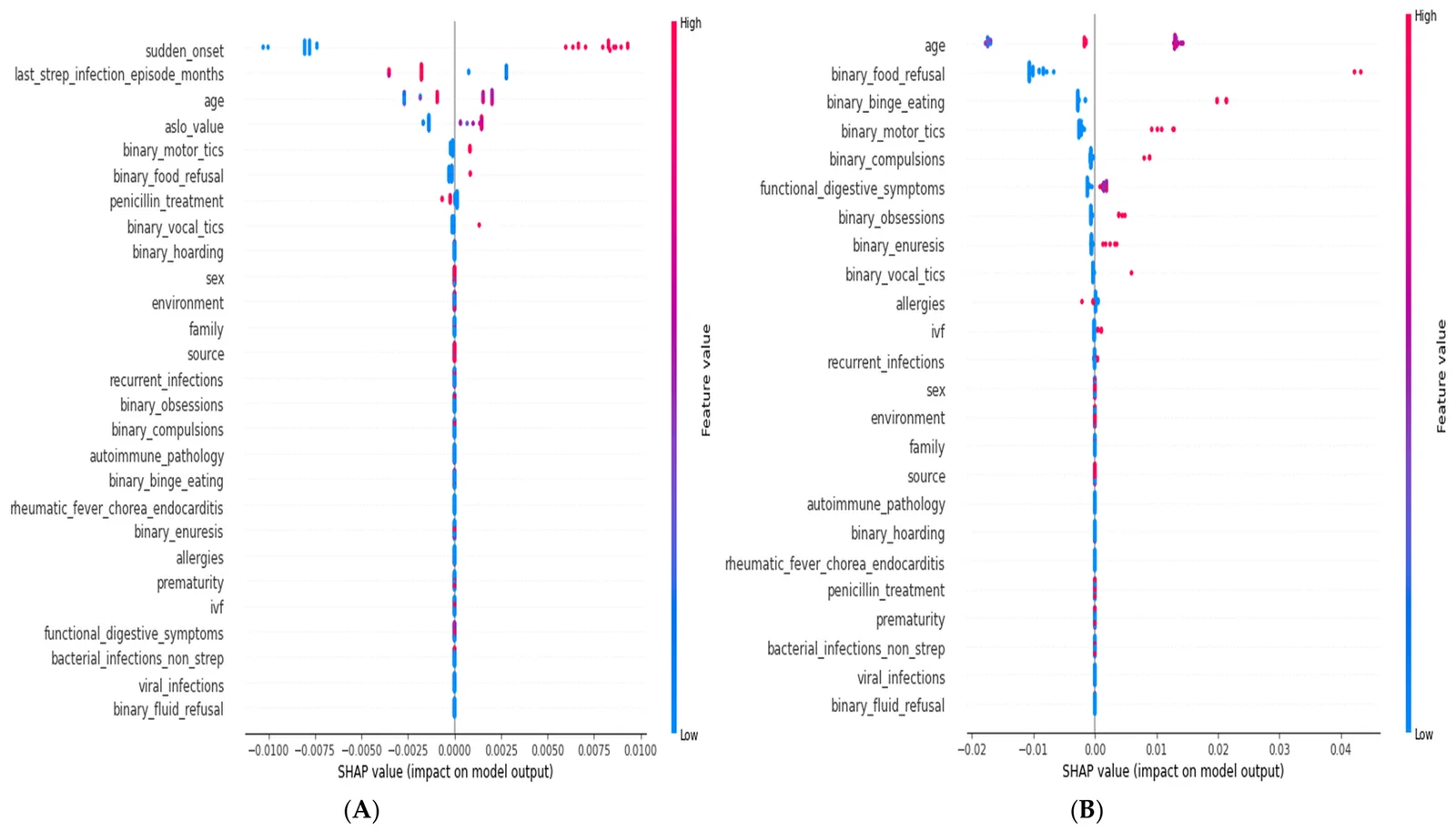
SHAP beeswarm plots for the XGBoost models. (**A**) Full-feature XGBoost model. (**B**) Restricted-feature XGBoost model. The plots illustrate predictor attribution within the fitted XGBoost models.

Model-specific SHAP beeswarm plots are shown in Figure 5, Figure 6 and Figure 7 as descriptive complements to the global SHAP summaries. By displaying the distribution and direction of feature-attribution values across individual observations, these plots help illustrate how each model used the leading predictors in the full-feature and restricted-feature settings. They were interpreted as model-behaviour visualizations only.

The variables highlighted in the beeswarm plots were clinically meaningful but should not be overinterpreted. In the full-feature setting, infection recency, abrupt onset, and ASO value reflected the core serological–temporal–onset structure of the operational profile, whereas selected PANS-31 item-level indicators such as motor tics, food refusal, binge eating, obsessions, and compulsions represented symptom-domain expression. Age also appeared among relevant attribution variables, but this finding should be interpreted cautiously because age may reflect developmental stage, referral patterns, symptom recognition, or age-related differences in exposure and serological response rather than a specific biological effect. Sex showed descriptive imbalance in the cohort, but it was not treated as evidence of sex-specific disease biology. In the restricted-feature setting, the shift toward age, residual symptom indicators, functional digestive symptoms, and contextual variables suggested weaker but clinically plausible residual structure after removal of the most direct endpoint-related predictors.

## 4. Discussion

### 4.1. Principal Findings

This study examined whether clinical elements commonly considered together in suspected streptococcal-associated pediatric neuropsychiatric presentations also formed a coherent analytical pattern within the cohort. Its main contribution is the transparent reconstruction of a predefined ASO–infection timing–abrupt onset–PANS-31 profile using interpretable supervised learning, supporting structured phenotyping and hypothesis generation within a clinically enriched pediatric psychiatric cohort.

The restricted-feature refit is central to this interpretation. When continuous ASO value, infection recency, and abrupt onset were removed, discrimination decreased substantially. This shows that the strongest full-feature performance was driven by clinically expected information close to the endpoint, rather than by an independent high-dimensional signature. The persistence of modest restricted-feature discrimination suggests that symptom-domain and contextual variables still carried some residual structure within the cohort. Accordingly, the restricted-feature analysis helps contextualize the conceptual overlap between the operational endpoint and several predictors retained in the full-feature models by showing how much discriminative structure remains once the most endpoint-proximal variables are removed.

SHAP-based interpretation supported this reading. In the full-feature setting, attribution converged on infection recency, abrupt onset, continuous ASO value, age, and selected PANS-31 item-level indicators, including motor tics, food refusal, binge eating, obsessions, and compulsions. In the restricted-feature setting, attribution shifted toward age, residual PANS-31 item-level indicators, functional digestive symptoms, and broader contextual or systemic variables. This shift helps explain why restricted-feature models estimated a weaker residual signal rather than reproducing the full clinical–serological pattern.

The principal contribution of this modelling framework is methodological and hypothesis-generating rather than diagnostic. By comparing full-feature and restricted-feature models, the analysis quantifies the extent to which discrimination of a rule-defined clinical–serological profile depends on variables closely related to its construction, while exploring the weaker residual patterns associated with symptom-domain and contextual information. These findings may help prioritize variables and methodological considerations for future investigations using independently characterized clinical outcomes and appropriate comparator populations, but they do not establish a disease-specific signature or demonstrate clinical diagnostic utility. In a field where diagnostic labels remain contested and where isolated serological, symptomatic, or temporal markers are insufficient on their own, the present approach shows how a clinically predefined profile can be tested for internal coherence, dependence on core clinical variables, and residual signal after removal of endpoint-proximal information. This gives the analysis methodological relevance beyond the specific cohort by illustrating how interpretable modelling can support structured phenotyping in diagnostically uncertain clinical settings.

### 4.2. Interpretation Within the PANS/PANDAS Clinical Field

The findings should be interpreted within a field in which diagnostic boundaries remain clinically important but unsettled. This distinction is particularly relevant to the present cohort. The descriptive analysis of the same source cohort revealed that its clinical characteristics placed it in an intermediate position between highly selective classical descriptions of PANDAS and broader, more heterogeneous PANS-like acute neuropsychiatric presentations. The present secondary modelling analysis does not alter that clinical positioning. Rather, it examines the internal structure of a streptococcal-exposure-associated profile within this clinically enriched cohort without assigning either a PANS or PANDAS diagnosis. Current diagnostic reviews emphasize that PANDAS-oriented assessment requires integration of acute neuropsychiatric presentation, evidence of recent or previous streptococcal exposure, temporal plausibility, and exclusion of alternative explanations; antistreptococcal titers may support evidence of exposure but are not disease-specific diagnostic tests [17]. Retrospective clinical series similarly describe heterogeneous presentations, with variable combinations of obsessive-compulsive symptoms, tics, eating disturbance, affective symptoms, behavioral dysregulation, somatic complaints, and infection-related information [18].

The serological component of the present findings warrants particularly cautious clinical interpretation. ASO is best understood as a marker of previous humoral immune response to group A streptococcal exposure, not as proof of active infection, neuropsychiatric causality, or disease specificity.

Although a post-streptococcal immune response is biologically compatible with proposed autoimmune mechanisms in PANDAS, including molecular mimicry and antineuronal reactivity, ASO itself does not demonstrate neuroimmune involvement. In the present study, ASO therefore contributed to the reconstruction of an exposure-associated clinical–serological profile rather than serving as a stand-alone immunological signature of PANDAS [8,9,17]. Standardized throat culture or rapid antigen documentation at onset, serial ASO and anti-DNase B testing, and carrier-state assessment were not systematically available, limiting the temporal and etiological interpretation of the serological findings.

The profile reconstructed by the full-feature models was not reducible to ASO alone, symptom burden alone, or abrupt onset alone. It represented the convergence of serology, infection timing, onset pattern, and multidomain symptom burden. This is clinically plausible, because physicians evaluating suspected PANS/PANDAS presentations usually interpret these categories of information together. At the same time, the complete-information design suggests that the model reconstructed a rule-based profile rather than predicted an independently adjudicated diagnosis.

This distinction matters clinically. Diagnostic labels in the PANS/PANDAS field can influence family expectations, health care utilization, referral pathways, and treatment decisions. A recent Canadian surveillance study highlighted both the impact of PANS/PANDAS diagnostic labels and the diagnostic uncertainty surrounding their use in practice [19]. Consensus-oriented work has also emphasized the need for standardization of definitions, diagnostic criteria, treatment decisions, and follow-up strategies [20]. The present analysis therefore supports the internal coherence of this clinical–serological pattern within the cohort and provides a basis for prospective evaluation in more rigorously phenotyped populations.

### 4.3. Clinical Meaning of the Full-Feature and Restricted-Feature Strategy

The two-level modelling strategy is central to the interpretation of the findings. The full-feature setting reflects a complete-information clinical scenario in which ASO value, reported infection timing, onset pattern, symptom-domain indicators, and contextual variables are available at baseline. High internal discrimination in this setting indicates that the predefined ASO–infection timing–abrupt onset–PANS-31 pattern was coherent and recoverable within the dataset. Because several full-feature variables were clinically close to the rule-defined endpoint, this result should be read as recovery of a predefined profile under complete-information conditions, not as discovery of an independent diagnostic signature.

The restricted-feature setting addressed a different question. By removing continuous ASO value, infection recency, and abrupt onset before refitting the models, the analysis tested whether the remaining symptom-domain, developmental, systemic, infection-history, and contextual variables still carried discriminative information. Performance decreased in all three model families, and sensitivity fell to 0.429 across restricted-feature models, while specificity remained comparatively higher. This pattern indicates that the restricted models were less able to identify endpoint-positive cases once the most clinically central serological, temporal, and onset-related information was removed.

This attenuation is analytically informative because it localizes the dominant discriminative signal to the clinically central combination of serology, timing, onset pattern, and symptom burden. Symptom-domain and contextual variables alone did not reconstruct the profile with the same reliability. Clinically, this is intuitive: PANS/PANDAS-oriented reasoning depends heavily on timing, onset pattern, and evidence of streptococcal exposure, not only on symptom phenomenology. Therefore, the restricted-feature refit functions as the key sensitivity analysis, while the full-feature setting functions as complete-information reconstruction of the predefined profile.

### 4.4. Relation to Previous Computational Work

Computational approaches to PANS/PANDAS-like presentations remain limited. Gagliano and colleagues used data mining to examine whether PANS could be represented as a specific constellation of clinical variables [21]. Although their design, cohort, and analytic strategy differ from the present study, the conceptual problem is similar: heterogeneous pediatric neuropsychiatric presentations may contain recognizable multidomain structure that is difficult to summarize with single variables.

The present study extends this line of work by focusing specifically on a streptococcal-exposure-associated clinical–serological pattern and by explicitly testing the dependence of model discrimination on endpoint-proximal information. This is important because high model performance in clinically defined syndromes may otherwise be misread as discovery of a diagnostic signature. Here, the modelling strategy was used more deliberately: the full-feature analysis tested whether the predefined profile was internally recoverable, whereas the restricted-feature refit tested how much signal remained after removal of the most clinically central serological, temporal, and onset-related variables. This makes the analysis a phenotype-structure study rather than a conventional diagnostic AI study.

### 4.5. Logistic Regression, Tree-Based Models, and Small-Cohort Modelling

An important methodological observation is that more complex models did not uniformly dominate Logistic Regression. In the full-feature setting, XGBoost showed the highest held-out ROC-AUC. In the restricted-feature setting, Logistic Regression showed the most stable held-out discrimination. This is not surprising. A systematic review of clinical prediction modelling found no consistent performance advantage of machine-learning methods over logistic regression across clinical prediction studies [22]. In small or structured datasets, simpler models may capture the dominant signal more stably, particularly when the number of events is limited.

This is directly relevant here. The full analytical dataset included 96 participants, but only 24 were endpoint-positive. The held-out test partition included 29 participants, with only seven endpoint-positive cases. Under these conditions, apparent differences between classifiers should be interpreted cautiously. Model stability depends on event count, sample size, modelling strategy, and validation design, and small datasets can produce optimistic or unstable performance estimates even when internal cross-validation and held-out testing are used [23]. This limitation also implies a non-negligible risk of overfitting, particularly in the full-feature setting, where 27 predictors were evaluated in a cohort containing only 24 endpoint-positive participants. Although stratified cross-validation and held-out testing provided internal safeguards, they cannot eliminate overfitting in a dataset of this size; consequently, the reported discrimination may remain optimistic and requires confirmation in independent cohorts. More broadly, transparent clinical prediction modelling requires that the dataset, endpoint, predictors, modelling strategy, and validation design are matched to the intended clinical question rather than evaluated only through apparent discrimination [13,24].

Therefore, the comparison between Logistic Regression, Random Forest, and XGBoost should not be read as evidence of algorithmic superiority. The more important conclusion is that the full-feature signal was sufficiently structured to be captured by different model families, while restricted-feature performance became weaker and more model-dependent after removal of endpoint-proximal variables.

### 4.6. SHAP-Based Interpretation

SHAP analysis added interpretive value by showing how fitted models used the available variables. In the full-feature setting, the leading features were clinically coherent: infection recency, abrupt onset, continuous ASO value, age, and selected PANS-31 symptom indicators. In the restricted-feature setting, attribution shifted toward residual symptom-domain, age-related, systemic, and contextual variables. This shift paralleled the performance attenuation and supports the interpretation that the restricted models estimated residual clinical signal in comparison with the full clinical–serological pattern.

SHAP values were interpreted descriptively as measures of model behaviour within the available dataset [25,26]. Their value in the present analysis lies in showing which clinical dimensions contributed most strongly to model attribution and how these attribution patterns changed after endpoint-proximal variables were removed.

### 4.7. Broader Phenotypic and Systemic Context

The PANS–ASI/CSI convergence analysis in the initial phase of this study supports the use of PANS-31 as a dimensional symptom-burden measure in this cohort. The strongest theoretical correspondences were observed between matched domains such as anxiety, eating disturbance, behavioral/conduct symptoms, obsessive compulsive symptoms, motor symptoms, urinary/enuresis symptoms, psychotic-like symptoms, and vocal symptoms. These findings support domain-level clinical coherence and reinforce the value of dimensional PANS-31 assessment, while formal score strata remain a question for future psychometric validation.

The broader phenotypic findings provide descriptive clinical context for the residual information retained after removal of the most endpoint-proximal predictors. Functional digestive symptoms and prematurity were more frequent in the higher PANS-31 symptom-burden subgroup, while functional digestive symptoms also appeared among the variables contributing to model attribution in some restricted-feature analyses.

These observations should be interpreted as cohort-specific clinical associations rather than evidence of an underlying gastrointestinal, immunological, or other biological mechanism. Although microbiome-related and oxidative-stress pathways have been investigated in previous PANS/PANDAS research [27,28], the present study did not directly assess these processes. Neither the observed subgroup differences nor SHAP-based feature attribution can establish biological mechanisms, causal relationships, or disease-specific associations.

Nevertheless, these descriptive observations may inform future multidisciplinary investigations examining gastrointestinal symptoms, developmental history, infection-related variables, and neuropsychiatric symptom burden in larger, prospectively characterized pediatric cohorts.

Similarly, autoimmune pathology, recurrent infections, prematurity, and IVF history were retained as clinical-history and contextual variables reflecting the heterogeneity of the source cohort. Their inclusion should not be interpreted as evidence of causal or disease-specific vulnerability.

### 4.8. Strengths

This study has several strengths. First, it uses a transparent operational endpoint derived from clinically interpretable dimensions: symptom burden, serological status, infection timing, and abrupt onset. The endpoint was not discovered by an unconstrained algorithmic search, but was defined from variables that reflect real-world PANS/PANDAS-oriented clinical reasoning.

Second, the analysis explicitly distinguishes between complete-information reconstruction and restricted-feature sensitivity testing. This is a central methodological strength because it directly addresses the concern that models may simply recover variables close to the endpoint. Rather than ignoring this issue, this study uses it analytically: the full-feature model evaluates profile recoverability when clinically central information is available, while the restricted-feature model estimates residual signal after removal of the most endpoint-proximal predictors.

Third, this study combines discrimination metrics with interpretable model inspection. SHAP results showed that the models relied on clinically meaningful variables and that attribution changed in the expected direction after endpoint-proximal variables were removed. This supports the internal coherence of the modelling framework and provides reassurance that performance was not obviously driven by arbitrary or uninterpretable signals.

Fourth, this study provides a practical methodological template for future AI-assisted phenotyping studies in diagnostically uncertain pediatric neuropsychiatric conditions. By comparing complete-information models with restricted-feature refits, the analysis demonstrates how machine-learning performance can be used not only to classify a predefined endpoint, but also to interrogate the dependence of that classification on clinically central versus residual contextual information.

### 4.9. Limitations

Several limitations define the interpretive boundary of this study. First, the cohort was small, clinically enriched, and ASO-stratified, and therefore not representative of the general pediatric population. The modest sample size (*n* = 96) may also have affected model stability and increased uncertainty around internal performance estimates, reinforcing the need for confirmation in larger independent cohorts. No healthy control group, independent disease-control group, or external validation cohort was available. Accordingly, the current findings are based exclusively on internal validation and require confirmation through external validation in independent cohorts before their generalizability can be established.

Several baseline variables, including reported infection timing, abrupt onset or abrupt worsening, and symptom-scale information, relied partly on caregiver or patient report and available clinical documentation. This may have introduced recall, reporting, or documentation bias. Although ASO values provided an objective serological marker of previous streptococcal exposure, they could not independently establish infection timing, active infection, carrier status, or causal attribution to the neuropsychiatric presentation. In addition, because participants were recruited through specialist psychiatric inpatient and outpatient pathways rather than population-based screening or prospective flare surveillance, the cohort may not capture the full distribution of PANS-31 total scores in the general pediatric population or across other clinical pathways. In particular, very high PANS-31 total scores were not broadly represented in the present sample, and some children may have been assessed outside the peak of symptom exacerbation. Because validated total-score severity categories have not been established for the PANS-31, this observation should not be interpreted as indicating the presence or absence of a formally defined severe subgroup. These characteristics may limit inference about the full spectrum of symptom burden during acute flare states.

Second, the endpoint was operational and rule-based. It was derived from baseline clinical, serological, psychometric, and caregiver-informed variables; it was not assigned through blinded multidisciplinary adjudication and should not be considered equivalent to physician-confirmed PANDAS.

Third, several full-feature predictors were conceptually close to endpoint components. The endpoint included PANS-31 > 36, ASO > 200 IU/mL, abrupt onset or abrupt worsening, and recent reported streptococcal infection. Because related variables were available in the full-feature setting, full-feature performance represents internal reconstruction rather than independent diagnostic prediction.

Fourth, the held-out test set included only 29 participants, of whom seven were endpoint-positive. Sensitivity, specificity, positive predictive value, and negative predictive value are therefore statistically unstable and should not be interpreted as clinically transportable performance estimates.

Fifth, streptococcal exposure was reconstructed from caregiver report, available clinical documentation, and baseline ASO measurement. Consistent with the source observational cohort, ASO was interpreted as an accessible serological marker compatible with previous streptococcal exposure, with temporal context derived separately from the reported infection history; it was not treated as evidence of active infection or disease-specific causality. At baseline, participants did not present clinical signs suggestive of active streptococcal infection or systemic post-streptococcal complications; therefore, throat swab or streptococcal culture was not systematically obtained as part of routine clinical care. Serial ASO and anti-DNase B titers were not systematically available, streptococcal carrier status was not assessed, and prior antibiotic exposure may have influenced infection documentation or antibody dynamics. These limitations restrict interpretation of the infectious axis to reported exposure and serological context rather than prospectively confirmed streptococcal triggering.

Sixth, calibration, Brier score estimation, decision-curve analysis, clinically meaningful probability thresholds, subgroup fairness analyses, and external validation were not performed. ROC-AUC evaluates discrimination but does not establish whether predicted probabilities are reliable, calibrated, or clinically useful without formal calibration and clinical-utility assessment [29,30]. In the present study, because the models were not intended to provide patient-level risk probabilities, support individualized clinical decision-making, or guide a predefined intervention threshold, calibration assessment and decision-curve analysis were considered premature and outside the scope of this internal profile-reconstruction study. Accordingly, predicted probabilities should not be interpreted as calibrated clinical risks, and the reported ROC-AUC values should be read only as measures of internal discrimination and profile recoverability.

### 4.10. Future Directions

Future studies should move beyond internal profile reconstruction toward externally validated clinical modelling. A logical next step would be prospective evaluation in independent multicenter cohorts using harmonized recruitment procedures, prespecified endpoint definitions, and standardized predictor acquisition. External performance should be assessed across participating centers, including discrimination, calibration, model stability, and between-site heterogeneity, before any consideration of clinical translation. Larger, independent, and less serologically enriched cohorts will be necessary to determine whether the patterns observed here persist across broader clinical populations. Prospective designs should incorporate standardized documentation of infection timing, throat culture or rapid antigen testing when clinically indicated, serial ASO and anti-DNase B titers, assessment of streptococcal carrier status, longitudinal symptom trajectories, and blinded multidisciplinary endpoint adjudication.

Establishing clinical specificity would require comparisons with appropriately selected pediatric populations that share individual components of the operational profile but differ in their clinical presentation or infectious history. Children with acute-onset neuropsychiatric symptoms without evidence of recent streptococcal exposure, including presentations associated with other infectious or inflammatory conditions, would help determine whether abrupt onset and multidimensional symptom burden are specific to the investigated clinical–serological context. Children with chronic obsessive-compulsive disorders, tic disorders, or other psychiatric presentations without abrupt deterioration would provide clinically relevant comparisons for distinguishing symptom-domain overlap from onset-related characteristics. An additional important comparator would consist of children with microbiologically documented group A streptococcal infection but without corresponding neuropsychiatric deterioration, allowing for a more precise evaluation of the relationship between streptococcal exposure, serological findings, and psychiatric manifestations. Unselected pediatric psychiatric populations would also help assess the influence of referral patterns and clinical enrichment on the observed associations. Healthy or community-based controls could provide complementary reference distributions but would not replace these clinically relevant comparator groups.

Such investigations would require standardized clinical and psychometric assessments, improved microbiological documentation, longitudinal characterization of symptom trajectories, and independent multidisciplinary assessment of the clinical phenotypes under investigation. Importantly, future models intended to evaluate clinical specificity should be assessed against independently characterized clinical outcomes rather than exclusively against a composite endpoint constructed from the same serological, temporal, and symptom-related variables. The present study does not establish whether the reconstructed profile distinguishes PANDAS from other pediatric neuropsychiatric or post-infectious presentations. Instead, it provides an exploratory framework that may inform the design of future comparative studies addressing clinical specificity and generalizability.

In parallel, further psychometric work is needed to refine the interpretation of dimensional symptom burden. Future studies should examine whether clinically interpretable PANS-31 total-score strata or multidimensional symptom-burden categories can be established and externally validated across different clinical states and populations. Such work may help distinguish lower-range accumulation of relatively nonspecific psychiatric symptoms from more clearly expressed multidomain presentations relevant to the PANS clinical spectrum. Longitudinal assessment would be particularly valuable for determining whether changes in PANS-31 burden track clinically meaningful fluctuations over time and whether dimensional profiles are more informative than fixed dichotomous thresholds.

Beyond clinical and psychometric refinement, the present framework may inform the design of future AI-based diagnostic studies by emphasizing the distinction between internal reconstruction of a rule-defined clinical–serological profile and independent diagnostic prediction. Models incorporating ASO values, infection timing, abrupt onset, and dimensional symptom burden may be useful for testing whether a predefined phenotype is internally coherent. More broadly, interpretable machine-learning approaches of this type may provide a useful framework for hypothesis generation and exploratory patient stratification in clinical research by identifying clinically meaningful combinations of features that can subsequently be tested and validated in independent cohorts. However, restricted-feature refits, leakage checks, sensitivity analyses, and ultimately external validation remain necessary to determine how much discrimination persists once variables conceptually close to the endpoint are removed and whether any residual signal generalizes to independent populations.

Future AI studies should also clearly separate distinct clinical tasks. Diagnostic support, prognosis, relapse prediction, treatment-response prediction, and patient stratification require different study designs, endpoints, follow-up strategies, calibration assessment, clinical-utility analysis, external validation, subgroup evaluation, and model updating before decision-support use can be considered [31]. Explainability methods should support transparency and model inspection, but should not be used to infer biological causality or disease mechanisms without independent clinical and experimental evidence.

Taken together, future progress will require prospective clinical phenotyping, stronger psychometric calibration, appropriately selected comparator populations, and independent model validation before computational approaches can move from exploratory phenotype reconstruction toward clinically meaningful prediction.

## 5. Conclusions

In this clinically enriched and ASO-stratified pediatric psychiatric cohort, interpretable supervised models internally reconstructed a predefined streptococcal-exposure-associated clinical–serological profile. High full-feature discrimination supported the coherence of an ASO–infection timing–abrupt onset–PANS-31 pattern when clinically central information was available, whereas attenuated restricted-feature performance showed that this signal depended substantially on those core clinical–serological dimensions.

These findings support internal phenotype reconstruction rather than causal or patient-level diagnostic inference. They show that reported streptococcal exposure history, serological context, infection timing, abrupt onset, and dimensional psychiatric assessment can be integrated within a transparent and interpretable analytical framework. Prospective evaluation and external validation in independent cohorts are required before clinical translation. For now, the framework is best viewed as a tool for exploratory phenotyping and hypothesis generation in a clinically complex pediatric neuropsychiatric setting.

## Figures and Tables

**Table 1:** Variable handling and analytical feature-set structure.

Panel 1a. Variables collected, considered, and retained in the modelling workflow.				
Variable group		Collected in source cohort	Considered as candidate predictor	Retained in final modelling matrix
Age, sex, environment, family, care setting		Yes	Yes	Yes
Time since last streptococcal infection		Yes	Yes	Yes
Recurrent infections		Yes	Yes	Yes
Continuous ASO value		Yes	Yes	Yes
ASO category/binary ASO		Yes	Yes	No, redundant with endpoint threshold and continuous ASO
Abrupt onset		Yes	Yes	Yes
Penicillin treatment		Yes	Yes	Yes
Obsessions, compulsions, hoarding		Yes	Yes	Yes, binarized as >2
Food refusal, binge eating, fluid refusal		Yes	Yes	Yes, binarized as >2
Motor tics, vocal tics		Yes	Yes	Yes, binarized as >2
Enuresis		Yes	Yes	Yes, binarized as >2
Urinary frequency		Yes	Yes	No, not retained in final model
Hallucinations, delirium		Yes	Yes	No, not retained in final model
Allergies/atopic background		Yes	Yes	Yes
Prematurity		Yes	Yes	Yes
IVF history		Yes	Yes	Yes
Functional digestive symptoms		Yes	Yes	Yes
Non-streptococcal bacterial infections		Yes	Yes	Yes
Viral infections		Yes	Yes	Yes
Rheumatic fever/chorea/endocarditis history		Yes	Yes	Yes
Autoimmune pathology		Yes	Yes	Yes
ASI/CSI symptom domains		Yes	Exploratory only	No
EEG descriptors		Yes	Exploratory only	No
IQ category		Yes	Exploratory only	No
Diagnosis codes, psychiatric record, trauma, addictions		Yes	Exploratory only	No
Treatment response		Yes	Exploratory only	No
Panel 1b. Full-feature and restricted-feature analytical settings.				
Feature set	No. of predictors	Predictors included	Predictors excluded	Analytical purpose
Full-feature set	27	Demographic/contextual variables, infection-history variables, continuous ASO value, infection recency, abrupt onset, selected PANS-31 item-level indicators, developmental/systemic variables	PANS-31 total score, PANS-31 > 36, ASO binary/category	Complete-information internal reconstruction of the operational profile
Restricted-feature set	24	Demographic/contextual variables, infection-history variables, selected PANS-31 item-level indicators, and developmental/systemic variables	Continuous ASO value, infection recency, abrupt onset, PANS-31 total score, PANS-31 > 36, ASO binary/category	Sensitivity analysis of residual symptom-domain and contextual signal

Note: ASO, antistreptolysin O; PANS-31, Pediatric Acute-onset Neuropsychiatric Syndrome 31-Item Symptom Rating Scale. The restricted-feature set was refitted from scratch and was not a masking or post hoc removal analysis.

**Table 2:** Retained hyperparameters for full-feature and restricted-feature models. Hyperparameters were selected using GridSearchCV with ROC-AUC optimization and stratified 5-fold cross-validation.

Model	Feature Setting	Retained Hyperparameters
Logistic Regression	Full-feature	C = 0.1; solver = liblinear; class_weight = balanced; random_state = 42; max_iter = 100
Logistic Regression	Restricted-feature	C = 0.1; solver = liblinear; class_weight = balanced; random_state = 42; max_iter = 100
Random Forest	Full-feature	n_estimators = 200; max_depth = 3; min_samples_leaf = 7; max_features = log2; bootstrap = True; class_weight = balanced; random_state = 42
Random Forest	Restricted-feature	n_estimators = 200; max_depth = 3; min_samples_leaf = 7; max_features = sqrt; bootstrap = True; class_weight = balanced; random_state = 42
XGBoost	Full-feature	n_estimators = 10; max_depth = 2; learning_rate = 0.001; colsample_bytree = 0.6; colsample_bylevel = 0.6; scale_pos_weight = 2.94; random_state = 42
XGBoost	Restricted-feature	n_estimators = 40; max_depth = 2; learning_rate = 0.001; colsample_bytree = 0.6; colsample_bylevel = 0.8; scale_pos_weight = 2.94; random_state = 42

Note: scale_pos_weight = 2.94 reflected the negative-to-positive class ratio in the training set. Full-feature models used all 27 retained predictors. Restricted-feature models were refitted after excluding continuous ASO value, infection recency, and abrupt onset.

**Table 3:** Cohort structure, operational endpoint distribution, and internal train–test partition. The operational endpoint required PANS-31 total score > 36, ASO > 200 IU/mL, abrupt symptom onset or abrupt clinical worsening, and less than six months since the last reported streptococcal infection.

Item	Value
Screened cases	154
Analytical cohort	96
Elevated ASO stratum, ASO > 200 IU/mL	50
Non-elevated ASO stratum, ASO ≤ 200 IU/mL	46
PANS-31 total score > 36	34
Operational endpoint-positive	24
Operational endpoint-negative	72
Training set	67
Held-out test set	29
Endpoint-positive in training set	17
Endpoint-negative in training set	50
Endpoint-positive in held-out test set	7
Endpoint-negative in held-out test set	22

**Table 4:** Pre-modelling exploratory signals supporting the clinical rationale for the operational clinical–serological profile. These analyses summarize descriptive associations from the ASO/PANS statistical workflow. These analyses were used to contextualize the modelling rationale and were not used for automatic feature selection.

Exploratory Signal	Statistical Test	Statistic	*p*-Value	Relevance for Modelling
ASO value associated with PANS-31 total score	Spearman’s correlation	ρ = 0.806	*p* < 0.001	Supported clinical rationale for representing serological intensity
Time since last reported streptococcal infection associated with PANS-31 total score	Spearman’s correlation	ρ = −0.768	*p* < 0.001	Supported clinical rationale for representing temporal proximity
Time since last reported streptococcal infection associated with ASO value	Spearman’s correlation	ρ = −0.787	*p* < 0.001	Supported internal coherence between serological and temporal dimensions
Abrupt onset associated with PANS-31 total score	Mann–Whitney U test	U = 1848.0	*p* < 0.001	Supported clinical rationale for representing abrupt onset
Prematurity associated with PANS-31 total score	Mann–Whitney U test	U = 321.0	*p* < 0.001	Supported exploratory review of developmental-context variables
IVF history associated with PANS-31 total score	Mann–Whitney U test	U = 264.0	*p* = 0.008	Supported review of developmental/reproductive-history variables
Functional digestive symptoms associated with PANS-31 total score	Kruskal–Wallis test	H = 24.72	4.30 × 10^−6^	Supported exploratory review of systemic-context variables
PANS-31 convergence with ASI/CSI domains	Pearson’s correlations with FDR correction	43/121 significant domain-level correlations; strongest r = 0.899	FDR-adjusted *p* < 0.001 for strongest theoretical correspondences	Supported use of PANS-31 as a dimensional symptom-burden measure

Note: ASO, antistreptolysin O; PANS-31, Pediatric Acute-onset Neuropsychiatric Syndrome 31-Item Symptom Rating Scale; ASI/CSI, Adolescent/Child Symptom Inventory; IVF, in vitro fertilization. *p*-values reported as 0.000 in the statistical output are presented as *p* < 0.001.

**Table 5:** Internal performance of full-feature and restricted-feature models for reconstructing the operational streptococcal-exposure-associated clinical–serological profile. Full-feature models used all 27 retained predictors. Restricted-feature models were refitted after excluding continuous ASO value, time since the last reported streptococcal infection, and abrupt onset. Values represent internal estimates.

Model	Feature Setting	No. Predictors	CV ROC-AUC	Held-Out ROC-AUC	Sensitivity	Specificity
Logistic Regression	Full-feature	27	0.917	0.909	0.857	0.864
Logistic Regression	Restricted-feature	24	0.868	0.792	0.429	0.864
Random Forest	Full-feature	27	0.960	0.916	0.714	0.818
Random Forest	Restricted-feature	24	0.930	0.630	0.429	0.909
XGBoost	Full-feature	27	0.958	0.961	1.000	0.773
XGBoost	Restricted-feature	24	0.940	0.714	0.429	0.818

**Table 6:** Condensed summary of highest-ranked SHAP predictors. Predictors are summarized from mean absolute SHAP rankings within each model family. Full numerical SHAP rankings are provided in the Supplementary Materials.

Feature Setting	Model	Highest-Ranked SHAP Predictors
Full-feature	Logistic Regression	abrupt onset; food refusal; infection recency; age; motor tics
Full-feature	Random Forest	infection recency; abrupt onset; ASO value; age; food refusal
Full-feature	XGBoost	abrupt onset; infection recency; age; ASO value; motor tics
Restricted-feature	Logistic Regression	food refusal; binge eating; age; motor tics; penicillin treatment
Restricted-feature	Random Forest	age; food refusal; functional digestive symptoms; sex; motor tics
Restricted-feature	XGBoost	age; food refusal; binge eating; motor tics; compulsions

## Data Availability

The study protocol and analytical framework for the source observational cohort and linked AI/xAI modelling component were retrospectively registered on the Open Science Framework (OSF) on 26 April 2026. A view-only access link is available at: https://osf.io/vdezu/overview?view_only=6b2267a51fd7466abae3e58f80148ce8. The present manuscript reports a secondary model-focused analysis of the registered cohort. The operational streptococcal-exposure-associated clinical–serological profile analysed here was treated as an exploratory composite modelling endpoint within the registered AI/xAI framework, not as a physician-confirmed PANDAS diagnosis or validated diagnostic category. The analytical code, variable definitions, preprocessing procedures, model specifications, and aggregated results are provided in the Supplementary Materials. The participant-level clinical dataset forms part of an ongoing doctoral research project involving pediatric psychiatric patients and is not publicly available at the present stage, in accordance with applicable institutional, ethical, and data protection requirements.

## Supplementary Materials

The following supporting information can be downloaded at: https://www.mdpi.com/article/10.3390/children13101443/s1, Supplementary File S1 (children-13-01443_Analytical_Code_CLEAN.html): Annotated HTML version of the original Python analytical workflow, including exploratory analyses, preprocessing, supervised modelling, internal evaluation, and SHAP-based interpretation. Supplementary File S2 (Supplementary_Materials_AI_Model.docx): including source-cohort descriptive context, endpoint construction, variable handling, preprocessing, hyperparameter tuning, complete internal performance output, masking/no-retraining sensitivity checks, SHAP-based attribution, interpretive safeguards, and TRIPOD+AI-oriented reporting crosswalk. Supplementary File S2—Tables: Table S1. Source-cohort construction, retention, and analytical subgroup structure. Table S2. Relationship between the descriptive observational phase and the present supervised modelling phase. Table S3. Key descriptive distributions from the source-cohort observational phase. Table S3A. Stratified distribution of recorded non-streptococcal bacterial and viral infection histories in the source cohort. Table S4. Key exploratory statistical signals supporting the operational clinical–serological endpoint rationale. Table S5. PANS-31 psychometric and PANS–ASI/CSI convergence summary from the source-cohort descriptive workflow. Table S6. Correlations between individual ASI/CSI domains and PANS-31 total score. Table S7. Ancillary EEG source-cohort signals not carried into the final modelling matrices. Table S8. Extended variable dictionary and analytical handling across the observational and modelling workflow. Table S9, Panel A. Operational endpoint, cohort structure, and internal partitioning. Table S9, Panel B. Descriptive clinical characterization according to operational endpoint status. Table S10. Data harmonization and preprocessing safeguards. Table S11. Hyperparameter search spaces and retained settings. Table S12. Complete internal performance output for full-feature and restricted-feature refit models. Table S13. Sensitivity/specificity masking check using trained full-feature models: all predictors versus selected predictors. Table S14. Complete full-feature SHAP rankings by model family. Table S15. Complete restricted-feature SHAP rankings by model family. Table S16. Interpretive safeguards and reporting limitations. Table S17. TRIPOD+AI-oriented supplementary reporting crosswalk. Supplementary File S1—Figures: Figure S1. Participant flow, source-cohort structure, and internal modelling partition. Figure S2. Analytical workflow linking descriptive source-cohort context to internal supervised modelling. Figure S3. Feature-set and sensitivity-analysis logic. Supplementary File S3—(TRIPODAI_checklist_AI_Model.docx): completed TRIPOD+AI checklist.

## Abbreviations

The following abbreviations are used in this manuscript:

ANOVA	Analysis of variance
anti-DNase B	Anti-deoxyribonuclease B antibody
ASI	Adolescent Symptom Inventory
ASLO	Original source-dataset label for antistreptolysin O; the standard abbreviation used in this manuscript is ASO
ASO	Antistreptolysin O
CSI	Child Symptom Inventory
CV	Cross-validation
EEG	Electroencephalography
FDR	False discovery rate
GAS	Group A Streptococcus
IQ	Intelligence quotient
IQR	Interquartile range
IU/mL	International units per millilitre
IVF	In vitro fertilization
OSF	Open Science Framework
PANDAS	Pediatric Autoimmune Neuropsychiatric Disorders Associated with Streptococcal Infections
PANS	Pediatric Acute-onset Neuropsychiatric Syndrome
PANS-31	Pediatric Acute-onset Neuropsychiatric Syndrome 31-Item Symptom Rating Scale
ROC	Receiver operating characteristic
ROC-AUC	Area under the receiver operating characteristic curve
SHAP	SHapley Additive exPlanations
SMOTE	Synthetic Minority Oversampling Technique
TRIPOD+AI	Transparent Reporting of a multivariable prediction model for Individual Prognosis Or Diagnosis plus Artificial Intelligence
xAI	Explainable artificial intelligence
XGBoost	Extreme Gradient Boosting
