# Supplementary material for: Interpretable Modelling of a Streptococcal Exposure–Associated Neuropsychiatric Profile in a Pediatric Cohort
Source: Children (Basel). 2026 Oct 21;13(10):1443. doi: 10.3390/children13101443 (PMC13703277; doi:10.3390/children13101443)
Supplement: Supplementary file 1 [file children-13-01443-s001.zip › S1. children-4539230_Analytical_Code__clean_ANNOTATED.html]

analiza\_aslo\_stats\_clean


## Terminology and scope clarification (editorial annotation)

This supplementary HTML preserves the original analytical Python code, including exploratory procedures. Code-cell outputs have been omitted from this publication copy; the participant-level clinical dataset is not included.

In the original script, the variable `pandas` is the **binary, four-component operational clinical–serological endpoint** (PANS-31 total >36, ASO >200 IU/mL, documented abrupt symptom onset or worsening, and a reported interval of <6 months since the last streptococcal episode). It does **not** indicate a clinician-confirmed diagnosis of PANS or PANDAS. Legacy titles or printed messages referring to “PANS classification” should therefore be understood as modelling this operational endpoint.

The historical internal `source` label `Emergency` denotes **hospital-based psychiatric assessment** (`0` in the source dataset), contrasted with `Ambulatory` (`1` in the source dataset). These internal labels are retained as written to preserve the original code.

In [ ]:

```
import pandas as pd
```

In [ ]:

```
aslo_poz = pd.read_excel('STUDIU_COPII.xlsx', sheet_name='ASLO+', nrows=50)
aslo_neg = pd.read_excel('STUDIU_COPII.xlsx', sheet_name='ASLO-', nrows=46)
```

In [ ]:

```
# Rename columns to ensure consistency between ASLO+ and ASLO- datasets
aslo_neg.rename(columns={
    'DIAGNOSTIC INTERNARE': 'DIAGNOSTIC PRINCIPAL',
    'TULB. ALIMENTARE': 'REFUZ  ALIMENTAR',
    'DEF. PROSEXIC': 'DEFICIT PROSEXIC',
    'SCADERE PERFORMANTE SCOALRE': 'SCADERE PERFORMANTE SCOLARE',
    'DEF.COGNITIV/ MNEZIC': 'DEFICIT COGNITIV/ MNEZIC',
    'INFECTII_BACTERIENE_NOSTREP': 'INFECTII_BACTERIENE_NONSTREP',
}, inplace=True)

aslo_poz.rename(columns={
    'SENSIBILITATE SENZORIALA': 'HIPERSENSIBILITATE SENZORIALA',
    'TICURI  MOTORII': 'TICURI MOTORII',
    'TEREN ATOPIC': 'ALERGII',
}, inplace=True)
```

In [ ]:

```
df = pd.concat([aslo_poz, aslo_neg], ignore_index=True).fillna(0)
```

In [ ]:

```
# Create a comprehensive column renaming dictionary (Romanian to English snake_case)
column_mapping = {
    'VARSTA': 'age',
    'SEX': 'sex',
    'MEDIU': 'environment',
    'FAMILIE': 'family',
    'SURSA': 'source',
    'ultimul episod infectie Strep. (luni)': 'last_strep_infection_episode_months',
    'INFECTII REPETATE': 'recurrent_infections',
    'ASLO_VAL': 'aslo_value',
    'ASLO_CAT': 'aslo_category',
    'TEST ANTERIOR ASLO MODIFICAT': 'previous_aslo_test_modified',
    'TRATAMENT PEN_G': 'penicillin_treatment',
    'ALTE MODIFICARI HEMOLEUCOGRAMA': 'other_blood_count_changes',
    'TRAUMA PSIHICA': 'psychological_trauma',
    'DEBUT BRUSC': 'sudden_onset',
    'ADICTII': 'addictions',
    'EVIDENTA PSIHIATRICA': 'psychiatric_record',
    'DIAGNOSTIC PRINCIPAL': 'primary_diagnosis',
    'ASI/CSI_ADICTII': 'asi_csi_addictions',
    'ASI/CSI_ENUREZIS': 'asi_csi_enuresis',
    'ASI/CSI_VOCAL': 'asi_csi_vocal',
    'ASI/CSI_MOTOR': 'asi_csi_motor',
    'CSI_PERVAZIV_MAX': 'csi_pervasive_max',
    'ASI/CSI_PSIHOTIC_MAX': 'asi_csi_psychotic_max',
    'ASI_EATING_MAX': 'asi_eating_max',
    'ASI_CSI_TOC_MAX': 'asi_csi_ocd_max',
    'ASI/CSI_ANXIETATE_MAX': 'asi_csi_anxiety_max',
    'ASI/CSI_AFECTIV_MAX': 'asi_csi_affective_max',
    'ASI/CSI_CONDUITA_MAX': 'asi_csi_conduct_max',
    'PANS-31 TOTAL': 'pans_31_total',
    'OBSESII': 'obsessions',
    'COMPULSII': 'compulsions',
    'HOARDING': 'hoarding',
    'REFUZ  ALIMENTAR': 'food_refusal',
    'BINGE EATING': 'binge_eating',
    'REFUZ FLUIDE': 'fluid_refusal',
    'ANXIETATE DE SEPARARE': 'separation_anxiety',
    'ANXIETATE/FOBII': 'anxiety_phobias',
    'OSCILATII DISPOZITIE': 'mood_swings',
    'LABILITATE P-E': 'emotional_lability',
    'IDEATIE SUICIDARA/SELF-HARM': 'suicidal_ideation_self_harm',
    'DEPRESIE': 'depression',
    'IRITABILITATE': 'irritability',
    'OPOZITIONISM': 'oppositionism',
    'AGRESIVITATE': 'aggression',
    'IMPULSIVITATE/HIPERACTIVITATE': 'impulsivity_hyperactivity',
    'DEFICIT PROSEXIC': 'attention_deficit',
    'BABY-TALK': 'baby_talk',
    'REGRESIE COMPORTAMENT': 'behavioral_regression',
    'SCADERE PERFORMANTE SCOLARE': 'academic_performance_decline',
    'DISGRAFIE': 'dysgraphia',
    'DEFICIT COGNITIV/ MNEZIC': 'cognitive_memory_deficit',
    'CEFALEE': 'headache',
    'TULB SOMN': 'sleep_disturbance',
    'ENUREZIS': 'enuresis',
    'POLAKIURIE': 'urinary_frequency',
    'HIPERSENSIBILITATE SENZORIALA': 'sensory_hypersensitivity',
    'HALUCINATII': 'hallucinations',
    'DELIR': 'delirium',
    'TICURI MOTORII': 'motor_tics',
    'TICURI SONORE': 'vocal_tics',
    'QI': 'iq',
    'EEG': 'eeg',
    'EEG_ALFA_HIPOVOLTAT_TPO': 'eeg_alpha_low_voltage_tpo',
    'EEG_ALFA_DOMINANT_TPO': 'eeg_alpha_dominant_tpo',
    'EEG_TETA_POLIMORF': 'eeg_theta_polymorphic',
    'EEG_TETA_PAROXISTIC': 'eeg_theta_paroxystic',
    'EEG_COMPLEXE_VARF_UNDA': 'eeg_spike_wave_complexes',
    'EEG_MODIFICARI_NESPECIFICE': 'eeg_nonspecific_changes',
    'ALERGII': 'allergies',
    'PREMATURITATE': 'prematurity',
    'FIV': 'ivf',
    'SIMPTOME DIGESTIVE FUNCTIONALE': 'functional_digestive_symptoms',
    'INFECTII_BACTERIENE_NONSTREP': 'bacterial_infections_non_strep',
    'INFECTII_VIRALE': 'viral_infections',
    'RRA/COREE/ENDOCARDITA': 'rheumatic_fever_chorea_endocarditis',
    'PATOLOGIE AUTOIMUNA': 'autoimmune_pathology',
    'RASPUNS PSIHOTROPE/PSIHOTERAPIE': 'psychotropic_psychotherapy_response'
}

# Apply the column renaming
df.rename(columns=column_mapping, inplace=True)
```

In [ ]:

```
df.loc[df['last_strep_infection_episode_months']=='>12', 'last_strep_infection_episode_months'] = 12
df['last_strep_infection_episode_months'] = df['last_strep_infection_episode_months'].astype(int)

df['aslo_binary'] = df['aslo_value']>200
```

In [ ]:

```
# Display a sample of the dataframe to verify renamed columns
df.sample(5).T.head(20)
```

In [ ]:

```
# Convert allergies and autoimmune_pathology to binary (0/1)
# Original data has coded values: 0 = absent, non-zero codes = present
for col in ['allergies', 'autoimmune_pathology']:
    if col in df.columns:
        df[col] = (df[col] != 0).astype(int)

# Map numerical values to meaningful categories for 'sex' and 'environment' columns
df['sex'] = df['sex'].map({0: 'M', 1: 'F'})
df['environment'] = df['environment'].map({1: 'Rural', 0: 'Urban'})
df['family'] = df['family'].map({0: 'Family', 1: 'Foster Care'})
df['source'] = df['source'].map({0: 'Emergency', 1: 'Ambulatory'})
df['iq'] = df['iq'].map({1: 'Limit', 0: 'Normal'})
```

In [ ]:

```
# Import libraries for statistical analysis and plotting
import os
import matplotlib.pyplot as plt
import seaborn as sns
import numpy as np
from scipy import stats
from scipy.stats import chi2_contingency, pearsonr, spearmanr
import warnings
warnings.filterwarnings('ignore')

# Set plotting style
plt.style.use('default')
sns.set_palette("pastel")
plt.rcParams['figure.figsize'] = (12, 8)

# Centralized PNG export settings
PLOTS_DIR = 'plots'
PLOT_DPI = 300
os.makedirs(PLOTS_DIR, exist_ok=True)

def save_plot_png(filename, fig=None):
    """Save the active matplotlib figure or an explicit figure as PNG with white background."""
    target = os.path.join(PLOTS_DIR, filename)
    figure = fig if fig is not None else plt.gcf()
    figure.set_facecolor('white')
    figure.savefig(target, dpi=PLOT_DPI, bbox_inches='tight', facecolor='white')
    print(f"Saved plot: {target}")
```

In [ ]:

```
# ASLO vs PANS correlation analysis
aslo_pans_corr = df[['aslo_value', 'pans_31_total']].corr(method='spearman')
print("Correlation between ASLO value and PANS-31 total score (Spearman):")
print(aslo_pans_corr)

# scatter plot to visualize the relationship between ASLO value and PANS-31 total score with a regression line
plt.figure(figsize=(12, 8))
sns.scatterplot(x='aslo_value', y='pans_31_total', data=df)
plt.title('Scatter Plot of ASLO Value vs PANS-31 Total Score', fontsize=16)
plt.xlabel('ASLO Value', fontsize=14)
plt.ylabel('PANS-31 Total Score', fontsize=14)

# add the rho and p-value to the plot
rho, p_value = spearmanr(df['aslo_value'], df['pans_31_total'])
plt.text(0.05, 0.95, f'ρ = {rho:.2f}\np_value = {p_value:.3f}*', transform=plt.gca().transAxes, fontsize=14, verticalalignment='top')

x = df['aslo_value']
y = df['pans_31_total']

# Add regression line
z = np.polyfit(x, y, 1)
p = np.poly1d(z)
plt.plot(x, p(x), "r-", alpha=0.7)

save_plot_png('01_aslo_vs_pans_scatter.png')
plt.show()
```

# Statistical Analysis and Visualization¶

## PANS values correlations - Overview¶

This analysis explores the relationships between numerical and categorical features with PANS score using statistical tests and visualizations.

In [ ]:

```
# Table 1: Cohort Counts and Endpoint Prevalence

# Calculate overall counts
total_n = len(df)
df['pans_binary'] = df['pans_31_total'] > 36
df['aslo_binary'] = df['aslo_value'] > 200

# ASLO binary counts and percentages
aslo_neg_count = (~df['aslo_binary']).sum()
aslo_pos_count = df['aslo_binary'].sum()
aslo_neg_pct = (aslo_neg_count / total_n) * 100
aslo_pos_pct = (aslo_pos_count / total_n) * 100

# PANS binary counts and percentages
pans_below_count = (~df['pans_binary']).sum()
pans_above_count = df['pans_binary'].sum()
pans_below_pct = (pans_below_count / total_n) * 100
pans_above_pct = (pans_above_count / total_n) * 100

# 2x2 Crosstabulation of ASLO/PANS phenotypes
crosstab = pd.crosstab(df['aslo_binary'], df['pans_binary'], margins=True)
crosstab.index = ['ASLO Negative', 'ASLO Positive', 'Total']
crosstab.columns = ['PANS ≤36', 'PANS >36', 'Total']

print("=" * 80)
print("TABLE 1: COHORT COUNTS AND ENDPOINT PREVALENCE")
print("=" * 80)

# Display main statistics
print(f"\nTotal N: {total_n}\n")

print("ASLO Binary Classification:")
print(f"  ASLO Negative (≤200):  {aslo_neg_count:3d} ({aslo_neg_pct:5.1f}%)")
print(f"  ASLO Positive (>200):  {aslo_pos_count:3d} ({aslo_pos_pct:5.1f}%)")

print("\nPANS Binary Classification:")
print(f"  PANS ≤36:              {pans_below_count:3d} ({pans_below_pct:5.1f}%)")
print(f"  PANS >36:              {pans_above_count:3d} ({pans_above_pct:5.1f}%)")

print("\n2×2 Crosstabulation: ASLO/PANS Phenotypes")
print("-" * 80)
print(crosstab)
print("-" * 80)

# Create a nicely formatted pandas DataFrame for display
summary_data = {
    'Category': ['Total N', 'ASLO Negative (≤200)', 'ASLO Positive (>200)', 
                 'PANS ≤36', 'PANS >36'],
    'Count': [total_n, aslo_neg_count, aslo_pos_count, pans_below_count, pans_above_count],
    'Percentage': [100.0, aslo_neg_pct, aslo_pos_pct, pans_below_pct, pans_above_pct]
}
summary_df = pd.DataFrame(summary_data)
print("\nSummary Table:")
print(summary_df.to_string(index=False))

# ============================================================================
# TABLE 2: FEATURES DISTRIBUTION STRATIFIED BY ASLO STATUS
# ============================================================================

print("\n\n" + "=" * 80)
print("TABLE 2A: FEATURES DISTRIBUTION BY ASLO STATUS")
print("=" * 80)

# Define key features
key_features = ['age', 'sex', 'environment', 'family', 'source', 
                'last_strep_infection_episode_months', 'recurrent_infections',
                'aslo_value', 'penicillin_treatment', 'other_blood_count_changes',
                'psychological_trauma', 'sudden_onset', 'addictions', 'psychiatric_record',
                'prematurity', 'ivf', 'functional_digestive_symptoms',
                'bacterial_infections_non_strep', 'viral_infections', 'autoimmune_pathology']

# Numerical features
numerical_features = ['age', 'last_strep_infection_episode_months', 'aslo_value', 'pans_31_total']

# Categorical features
categorical_features = [f for f in key_features if f not in numerical_features]

print("\n--- A1) NUMERICAL FEATURES BY ASLO STATUS ---")
print("(Mean ± SD / Median [IQR])\n")

numerical_stats_aslo = []
for feature in numerical_features:
    row_data = {'Feature': feature}
    
    # ASLO Negative
    data_neg = df[~df['aslo_binary']][feature].dropna()
    if len(data_neg) > 0:
        mean_val = data_neg.mean()
        std_val = data_neg.std()
        median_val = data_neg.median()
        q1 = data_neg.quantile(0.25)
        q3 = data_neg.quantile(0.75)
        row_data['ASLO Negative (N=' + str(len(data_neg)) + ')'] = f"{mean_val:.1f}±{std_val:.1f} / {median_val:.0f}[{q1:.0f}-{q3:.0f}]"
    else:
        row_data['ASLO Negative'] = "N/A"
    
    # ASLO Positive
    data_pos = df[df['aslo_binary']][feature].dropna()
    if len(data_pos) > 0:
        mean_val = data_pos.mean()
        std_val = data_pos.std()
        median_val = data_pos.median()
        q1 = data_pos.quantile(0.25)
        q3 = data_pos.quantile(0.75)
        row_data['ASLO Positive (N=' + str(len(data_pos)) + ')'] = f"{mean_val:.1f}±{std_val:.1f} / {median_val:.0f}[{q1:.0f}-{q3:.0f}]"
    else:
        row_data['ASLO Positive'] = "N/A"
    
    numerical_stats_aslo.append(row_data)

numerical_stats_aslo_df = pd.DataFrame(numerical_stats_aslo)
print(numerical_stats_aslo_df.to_string(index=False))

print("\n\n--- A2) CATEGORICAL FEATURES BY ASLO STATUS ---")
print("(Count / Percentage within group)\n")

# Build a consolidated table for all categorical features by ASLO
table_data_aslo = []

for feature in categorical_features:
    if feature in df.columns:
        # ASLO Negative
        subset_neg = df[~df['aslo_binary']][feature]
        total_neg = len(subset_neg)
        value_counts_neg = subset_neg.value_counts()
        
        for val, count in value_counts_neg.items():
            pct = (count / total_neg) * 100
            row = {
                'Feature': feature,
                'ASLO Negative': f"{count} ({pct:.1f}%)",
            }
            table_data_aslo.append(row)
        
        # ASLO Positive
        subset_pos = df[df['aslo_binary']][feature]
        total_pos = len(subset_pos)
        value_counts_pos = subset_pos.value_counts()
        
        # Match with negative values to create proper rows
        all_values = set(value_counts_neg.index) | set(value_counts_pos.index)
        
        # Rebuild table with matching
        for val in sorted(all_values, key=str):
            count_neg = value_counts_neg.get(val, 0)
            count_pos = value_counts_pos.get(val, 0)
            pct_neg = (count_neg / total_neg * 100) if total_neg > 0 else 0
            pct_pos = (count_pos / total_pos * 100) if total_pos > 0 else 0
            
            row = {
                'Feature': feature,
                'Category': val,
                'ASLO Negative': f"{int(count_neg)} ({pct_neg:.1f}%)" if count_neg > 0 else "0 (0.0%)",
                'ASLO Positive': f"{int(count_pos)} ({pct_pos:.1f}%)" if count_pos > 0 else "0 (0.0%)"
            }
            table_data_aslo.append(row)

# Remove duplicate feature rows and keep only those with values
table_data_aslo_clean = []
current_feature = None
for row in table_data_aslo:
    if 'Category' in row:
        table_data_aslo_clean.append(row)

categorical_aslo_df = pd.DataFrame(table_data_aslo_clean)
print(categorical_aslo_df.to_string(index=False))

# ============================================================================
# TABLE 2B: FEATURES DISTRIBUTION STRATIFIED BY PANS STATUS
# ============================================================================

print("\n\n" + "=" * 80)
print("TABLE 2B: FEATURES DISTRIBUTION BY PANS STATUS")
print("=" * 80)

print("\n--- B1) NUMERICAL FEATURES BY PANS STATUS ---")
print("(Mean ± SD / Median [IQR])\n")

numerical_stats_pans = []
for feature in numerical_features:
    row_data = {'Feature': feature}
    
    # PANS Low (≤36)
    data_low = df[~df['pans_binary']][feature].dropna()
    if len(data_low) > 0:
        mean_val = data_low.mean()
        std_val = data_low.std()
        median_val = data_low.median()
        q1 = data_low.quantile(0.25)
        q3 = data_low.quantile(0.75)
        row_data['PANS ≤36 (N=' + str(len(data_low)) + ')'] = f"{mean_val:.1f}±{std_val:.1f} / {median_val:.0f}[{q1:.0f}-{q3:.0f}]"
    else:
        row_data['PANS ≤36'] = "N/A"
    
    # PANS High (>36)
    data_high = df[df['pans_binary']][feature].dropna()
    if len(data_high) > 0:
        mean_val = data_high.mean()
        std_val = data_high.std()
        median_val = data_high.median()
        q1 = data_high.quantile(0.25)
        q3 = data_high.quantile(0.75)
        row_data['PANS >36 (N=' + str(len(data_high)) + ')'] = f"{mean_val:.1f}±{std_val:.1f} / {median_val:.0f}[{q1:.0f}-{q3:.0f}]"
    else:
        row_data['PANS >36'] = "N/A"
    
    numerical_stats_pans.append(row_data)

numerical_stats_pans_df = pd.DataFrame(numerical_stats_pans)
print(numerical_stats_pans_df.to_string(index=False))

print("\n\n--- B2) CATEGORICAL FEATURES BY PANS STATUS ---")
print("(Count / Percentage within group)\n")

# Build a consolidated table for all categorical features by PANS
table_data_pans = []

for feature in categorical_features:
    if feature in df.columns:
        # PANS Low (≤36)
        subset_low = df[~df['pans_binary']][feature]
        total_low = len(subset_low)
        value_counts_low = subset_low.value_counts()
        
        # PANS High (>36)
        subset_high = df[df['pans_binary']][feature]
        total_high = len(subset_high)
        value_counts_high = subset_high.value_counts()
        
        # Match with both values
        all_values = set(value_counts_low.index) | set(value_counts_high.index)
        
        # Create rows for each value
        for val in sorted(all_values, key=str):
            count_low = value_counts_low.get(val, 0)
            count_high = value_counts_high.get(val, 0)
            pct_low = (count_low / total_low * 100) if total_low > 0 else 0
            pct_high = (count_high / total_high * 100) if total_high > 0 else 0
            
            row = {
                'Feature': feature,
                'Category': val,
                'PANS ≤36': f"{int(count_low)} ({pct_low:.1f}%)" if count_low > 0 else "0 (0.0%)",
                'PANS >36': f"{int(count_high)} ({pct_high:.1f}%)" if count_high > 0 else "0 (0.0%)"
            }
            table_data_pans.append(row)

categorical_pans_df = pd.DataFrame(table_data_pans)
print(categorical_pans_df.to_string(index=False))
```

In [ ]:

```
numerical_columns = ['age', 'last_strep_infection_episode_months',
       'asi_csi_addictions', 'asi_csi_enuresis', 'asi_csi_vocal', 'asi_csi_motor',
       'csi_pervasive_max', 'asi_csi_psychotic_max', 'asi_eating_max',
       'asi_csi_ocd_max', 'asi_csi_anxiety_max', 'asi_csi_affective_max',
       'asi_csi_conduct_max', 
       # 'pans_31_total', 
       # 'obsessions', 'compulsions',
       # 'hoarding', 'food_refusal', 'binge_eating', 'fluid_refusal',
       # 'separation_anxiety', 'anxiety_phobias', 'mood_swings',
       # 'emotional_lability', 'suicidal_ideation_self_harm', 'depression',
       # 'irritability', 'oppositionism', 'aggression',
       # 'impulsivity_hyperactivity', 'attention_deficit', 'baby_talk',
       # 'behavioral_regression', 'academic_performance_decline', 'dysgraphia',
       # 'cognitive_memory_deficit', 'headache', 'sleep_disturbance', 'enuresis',
       # 'urinary_frequency', 'sensory_hypersensitivity', 'hallucinations',
       # 'delirium', 'motor_tics', 'vocal_tics'
       ]
categorical_columns = ['sex', 'environment', 'family', 'source', 'penicillin_treatment', 
       'other_blood_count_changes', 'psychological_trauma', 'sudden_onset', 'addictions',
       'psychiatric_record', 'primary_diagnosis', 'iq', 'eeg_alpha_low_voltage_tpo', 
       'eeg_alpha_dominant_tpo', 'eeg_theta_polymorphic', 'eeg_theta_paroxystic',
       'eeg_spike_wave_complexes', 'eeg_nonspecific_changes', 'allergies',
       'prematurity', 'ivf', 'functional_digestive_symptoms',
       'bacterial_infections_non_strep', 'viral_infections',
       # 'rheumatic_fever_chorea_endocarditis', only 0
       'autoimmune_pathology',
       'psychotropic_psychotherapy_response']
target_column = 'pans_31_total'
```

In [ ]:

```
# Statistical Comparison Plots: Numerical and Categorical Columns vs Target Column
from scipy.stats import f_oneway, pearsonr, spearmanr, mannwhitneyu
from sklearn.preprocessing import LabelEncoder
import matplotlib.pyplot as plt
import seaborn as sns
import numpy as np
import pandas as pd

# Function to create plots for numerical columns
def plot_numerical_vs_target(df, numerical_cols, target_col):
    print(f"Numerical vs target statistics:")
    n_cols = len(numerical_cols)
    n_rows = (n_cols + 2) // 3  # 3 plots per row
    
    fig, axes = plt.subplots(n_rows, 3, figsize=(18, 7*n_rows))
    axes = axes.flatten() if n_rows > 1 else [axes] if n_cols == 1 else axes
    
    for i, col in enumerate(numerical_cols):
        ax = axes[i]
        
        # Remove NaN values for this comparison
        valid_data = df[[col, target_col]].dropna()
        
        if len(valid_data) < 3:
            ax.text(0.5, 0.5, f'Insufficient data\nfor {col}', 
                   ha='center', va='center', transform=ax.transAxes)
            ax.set_title(col)
            continue
        
        x = valid_data[col]
        y = valid_data[target_col]
        
        # Calculate correlation
        spearman_corr, spearman_p = spearmanr(x, y)
        
        # Create scatter plot
        ax.scatter(x, y, alpha=0.6, color='steelblue')
        
        # Add regression line
        z = np.polyfit(x, y, 1)
        p = np.poly1d(z)
        ax.plot(x, p(x), "r--", alpha=0.8)
        
        # Add statistics to plot        
        if spearman_p < 0.05 and (spearman_corr > 0.3 or spearman_corr < -0.3):
            stats_text = f'Spearman ρ: {spearman_corr:.3f} (p={spearman_p:.3f}) *'
            print(f"{col}: Spearman ρ = {spearman_corr:.3f}, p-value = {spearman_p:.3f} *")
        else:
            stats_text = f'Spearman ρ: {spearman_corr:.3f} (p={spearman_p:.3f})'

        ax.text(0.05, 0.95, stats_text, transform=ax.transAxes, 
               bbox=dict(boxstyle="round,pad=0.3", facecolor="white", alpha=0.8),
               verticalalignment='top', fontsize=13)
        
        ax.set_xlabel(col, fontsize=12)
        ax.set_ylabel(target_col, fontsize=12)
        ax.set_title(f'{col} vs {target_col}', fontsize=13)
        ax.grid(True, alpha=0.3)
    
    # Hide empty subplots
    for j in range(i+1, len(axes)):
        axes[j].axis('off')
    
    plt.tight_layout()
    return fig

# Function to create plots for categorical columns
def plot_categorical_vs_target(df, categorical_cols, target_col):
    print(f"Categorical vs target statistics:")
    n_cols = len(categorical_cols)
    n_rows = (n_cols + 2) // 3  # 3 plots per row
    
    fig, axes = plt.subplots(n_rows, 3, figsize=(18, 7*n_rows))
    axes = axes.flatten() if n_rows > 1 else [axes] if n_cols == 1 else axes
    
    for i, col in enumerate(categorical_cols):
        ax = axes[i]
        
        # Remove NaN values for this comparison
        valid_data = df[[col, target_col]].dropna()
        
        if len(valid_data) < 3:
            ax.text(0.5, 0.5, f'Insufficient data\nfor {col}', 
                   ha='center', va='center', transform=ax.transAxes)
            ax.set_title(col)
            continue
        
        # Get unique categories
        categories = valid_data[col].unique()
        
        if len(categories) < 2:
            ax.text(0.5, 0.5, f'Only one category\nfor {col}', 
                   ha='center', va='center', transform=ax.transAxes)
            ax.set_title(col)
            continue
        
        # Prepare data for statistical test
        groups = [valid_data[valid_data[col] == cat][target_col].values for cat in categories]
        groups = [group for group in groups if len(group) > 0]  # Remove empty groups
        
        if len(groups) < 2:
            ax.text(0.5, 0.5, f'Insufficient groups\nfor {col}', 
                   ha='center', va='center', transform=ax.transAxes)
            ax.set_title(col)
            continue
        
        # Choose appropriate statistical test based on number of groups
        if len(groups) == 2:
            # Use Mann-Whitney U Test for 2 groups (non-parametric alternative to t-test)
            try:
                statistic, p_value = mannwhitneyu(groups[0], groups[1], alternative='two-sided')
                test_name = "Mann-Whitney U"
            except:
                statistic, p_value = np.nan, np.nan
                test_name = "Mann-Whitney U (failed)"
        else:
            # Use ANOVA for 3+ groups
            try:
                statistic, p_value = f_oneway(*groups)
                test_name = "ANOVA"
            except:
                statistic, p_value = np.nan, np.nan
                test_name = "ANOVA (failed)"
        
        # Create box plot
        sns.boxplot(data=valid_data, x=col, y=target_col, ax=ax, palette='pastel')
        
        # Add individual data points as dots
        sns.stripplot(data=valid_data, x=col, y=target_col, ax=ax, 
                     color='black', alpha=0.6, size=3, jitter=True)
        
        # Rotate x-axis labels if needed
        if len(str(categories[0])) > 8 or len(categories) > 4:
            ax.tick_params(axis='x', rotation=45)
        
        # Add statistics to plot
        if p_value < 0.05:
            stats_text = f'{test_name} p-value: {p_value:.3f} *'
            print(f"{col}: {test_name} statistic = {statistic:.3f}, p-value = {p_value:.3f} *")
        else:
            stats_text = f'{test_name} p-value: {p_value:.3f}'
        
        ax.text(0.05, 0.95, stats_text, transform=ax.transAxes, 
               bbox=dict(boxstyle="round,pad=0.3", facecolor="white", alpha=0.8),
               verticalalignment='top', fontsize=13)
        
        ax.set_xlabel(col, fontsize=12)
        ax.set_ylabel(target_col, fontsize=12)
        ax.set_title(f'{col} vs {target_col}', fontsize=13)
        ax.grid(True, alpha=0.3)
    
    # Hide empty subplots
    for j in range(i+1, len(axes)):
        axes[j].axis('off')
    
    plt.tight_layout()
    return fig

# Create plots for numerical columns
print("=== NUMERICAL COLUMNS vs TARGET COLUMN ===")
print(f"Analyzing relationships between numerical variables and '{target_column}'")
print("Statistics shown: Spearman correlation with p-values")
print("-" * 70)
fig_num = plot_numerical_vs_target(df, numerical_columns, target_column)
save_plot_png('02_numerical_vs_pans_total.png', fig_num)
plt.show()

print("\n" + "="*70)
print("=== CATEGORICAL COLUMNS vs TARGET COLUMN ===")
print(f"Analyzing relationships between categorical variables and '{target_column}'")
print("Statistics shown: Mann-Whitney U Test (2 groups) or ANOVA F-statistic (3+ groups) with p-values")
print("-" * 70)
fig_cat = plot_categorical_vs_target(df, categorical_columns, target_column)
save_plot_png('03_categorical_vs_pans_total.png', fig_cat)
plt.show()
```

## ASLO values correlations - Overview¶

This analysis explores the relationships between numerical and categorical features with ASLO values using statistical tests and visualizations.

In [ ]:

```
numerical_columns = ['age', 'last_strep_infection_episode_months',
       'asi_csi_addictions', 'asi_csi_enuresis', 'asi_csi_vocal', 'asi_csi_motor',
       'csi_pervasive_max', 'asi_csi_psychotic_max', 'asi_eating_max',
       'asi_csi_ocd_max', 'asi_csi_anxiety_max', 'asi_csi_affective_max',
       'asi_csi_conduct_max', 'pans_31_total', 
    #    'obsessions', 'compulsions',
    #    'hoarding', 'food_refusal', 'binge_eating', 'fluid_refusal',
    #    'separation_anxiety', 'anxiety_phobias', 'mood_swings',
    #    'emotional_lability', 'suicidal_ideation_self_harm', 'depression',
    #    'irritability', 'oppositionism', 'aggression',
    #    'impulsivity_hyperactivity', 'attention_deficit', 'baby_talk',
    #    'behavioral_regression', 'academic_performance_decline', 'dysgraphia',
    #    'cognitive_memory_deficit', 'headache', 'sleep_disturbance', 'enuresis',
    #    'urinary_frequency', 'sensory_hypersensitivity', 'hallucinations',
    #    'delirium', 'motor_tics', 'vocal_tics'
       ]
categorical_columns = ['sex', 'environment', 'family', 'source', 'penicillin_treatment', 
       'other_blood_count_changes', 'psychological_trauma', 'sudden_onset', 'addictions',
       'psychiatric_record', 'primary_diagnosis', 'iq', 'eeg_alpha_low_voltage_tpo', 
       'eeg_alpha_dominant_tpo', 'eeg_theta_polymorphic', 'eeg_theta_paroxystic',
       'eeg_spike_wave_complexes', 'eeg_nonspecific_changes', 'allergies',
       'prematurity', 'ivf', 'functional_digestive_symptoms',
       'bacterial_infections_non_strep', 'viral_infections',
       # 'rheumatic_fever_chorea_endocarditis', only 0
       'autoimmune_pathology',
       'psychotropic_psychotherapy_response']
target_column = 'aslo_value'
```

In [ ]:

```
# Create plots for numerical columns
print("=== NUMERICAL COLUMNS vs TARGET COLUMN ===")
print(f"Analyzing relationships between numerical variables and '{target_column}'")
print("Statistics shown: Spearman correlation with p-values")
print("-" * 70)
fig_num_aslo = plot_numerical_vs_target(df, numerical_columns, target_column)
save_plot_png('02b_numerical_vs_aslo_value.png', fig_num_aslo)
plt.show()

print("\n" + "="*70)
print("=== CATEGORICAL COLUMNS vs TARGET COLUMN ===")
print(f"Analyzing relationships between categorical variables and '{target_column}'")
print("Statistics shown: Mann-Whitney U Test (2 groups) or ANOVA F-statistic (3+ groups) with p-values")
print("-" * 70)
fig_cat_aslo = plot_categorical_vs_target(df, categorical_columns, target_column)
save_plot_png('03b_categorical_vs_aslo_value.png', fig_cat_aslo)
plt.show()
```

In [ ]:

```
# Correlation heatmap for top numerical features
print("\n=== CORRELATION HEATMAP ===")

plt.figure(figsize=(16, 13))
correlation_matrix = df[[target_column] + numerical_columns].corr(method='spearman')
sns.heatmap(correlation_matrix, annot=True, cmap='RdBu_r', center=0,
            square=True, linewidths=0.5, fmt='.2f', annot_kws={'fontsize': 11})
plt.title('Spearman Correlation Matrix: Top 15 Numerical Features + ASLO Value', fontsize=16, pad=20)
plt.tight_layout()
save_plot_png('04_spearman_correlation_heatmap.png')
plt.show()
```

https://www.researchgate.net/figure/Interpretation-Table-of-Spearman-Rank-Order-Correlation-Coefficients\_tbl1\_267339455

# Advanced Analysis: Machine Learning & Pattern Recognition¶

## Predictive Modeling for PANS Classification¶

Building machine learning models to predict PANS > 36 status and identify most important clinical features.

In [ ]:

```
cols_to_binary = ['obsessions', 'compulsions', 'hoarding', 'food_refusal', 'binge_eating', 'fluid_refusal', 'motor_tics', 'vocal_tics', 'enuresis', 'urinary_frequency', 'hallucinations', 'delirium']

# transform to binary the columns with 0/1 values when their values are > 2 and create a new column with the binary_ prefix
for col in cols_to_binary:
    if col in df.columns:
        df['binary_' + col] = (df[col] > 2).astype(int)

binary_columns = ['binary_' + col for col in cols_to_binary if 'binary_' + col in df.columns]
```

In [ ]:

```
df['pandas'] = (df['pans_31_total'] > 36) & (df['aslo_value'] > 200) & (df['sudden_onset'] == 1) & (df['last_strep_infection_episode_months'] < 6)
selected_train_columns = [
       'age', 
       'sex', 
       'environment', 
       'family', 
       'source',
                 
       # 'last_strep_infection_episode_months', 
       'recurrent_infections',
       # 'aslo_value', 

       'binary_obsessions',
       'binary_compulsions',
       'binary_hoarding',
       'binary_food_refusal',
       'binary_binge_eating',
       'binary_fluid_refusal',
       'binary_motor_tics',
       'binary_vocal_tics',
       'binary_enuresis',

       'penicillin_treatment', 
       # 'sudden_onset',

       'allergies', 
       'prematurity', 
       'ivf', 
       'functional_digestive_symptoms',
       'bacterial_infections_non_strep', 
       'viral_infections',
       'rheumatic_fever_chorea_endocarditis', 
       'autoimmune_pathology',
       ]
train_columns = selected_train_columns
```

In [ ]:

```
# Import additional libraries for advanced analysis
from sklearn.model_selection import train_test_split, cross_val_score, StratifiedKFold
from sklearn.ensemble import RandomForestClassifier, GradientBoostingClassifier
from sklearn.linear_model import LogisticRegression
from sklearn.metrics import classification_report, confusion_matrix, roc_auc_score, roc_curve
from sklearn.preprocessing import StandardScaler, LabelEncoder
from sklearn.decomposition import PCA
from sklearn.cluster import KMeans
from scipy.cluster.hierarchy import dendrogram, linkage, fcluster
from scipy.spatial.distance import squareform
import plotly.express as px
import plotly.graph_objects as go
from plotly.subplots import make_subplots

print("Advanced analysis libraries imported successfully!")

# Prepare data for machine learning
def prepare_ml_data(df, target='pandas'):
    """Prepare data for machine learning by handling categorical variables and missing values"""
    
    # Create a copy
    ml_df = df.copy()
    
    # Remove non-predictive columns
    non_predictive_cols = ['pans_31_total']  # Don't use PANS value to predict PANS binary
    ml_df = ml_df.drop(columns=[col for col in non_predictive_cols if col in ml_df.columns])
    
    # Encode categorical variables
    categorical_features = ml_df.select_dtypes(include=['object']).columns
    le_dict = {}
    
    for col in categorical_features:
        if col != target:
            le = LabelEncoder()
            ml_df[col] = le.fit_transform(ml_df[col].astype(str))
            le_dict[col] = le
    
    # Separate features and target
    X = ml_df.drop(columns=[target])
    # X = X[X.columns[:-1]]
    y = ml_df[target].astype(int)
    
    # Fill any remaining missing values
    X = X.fillna(X.median())
    
    return X, y, le_dict

# Prepare the data
X, y, label_encoders = prepare_ml_data(df[train_columns + ['pandas']])
print(f"Prepared ML dataset: {X.shape[0]} samples, {X.shape[1]} features")
print(f"Target distribution: {y.value_counts().to_dict()}")
```

In [ ]:

```
from xgboost import XGBClassifier
from sklearn.model_selection import GridSearchCV, StratifiedKFold, train_test_split
from sklearn.metrics import roc_auc_score
import numpy as np
import pandas as pd

# Machine Learning Models for PANS Classification (with Grid Search)
print("=== MACHINE LEARNING CLASSIFICATION ANALYSIS ===")
print("Training and tuning models to predict PANS status from clinical features\n")

# NOTE (kept minimal for reproducibility + SHAP below):
# - This notebook uses label-encoded categoricals from `prepare_ml_data`.
# - A more 'correct' approach is ColumnTransformer + OneHotEncoder, but that changes feature space and would require updating the SHAP section.

X_train, X_test, y_train, y_test = train_test_split(
    X, y, test_size=0.3, random_state=42, stratify=y
 )
print(f"Training set: {X_train.shape[0]} samples")
print(f"Test set: {X_test.shape[0]} samples")

# Scale features for logistic regression (trees do not need scaling)
scaler = StandardScaler()
X_train_scaled = scaler.fit_transform(X_train)
X_test_scaled = scaler.transform(X_test)

# Class imbalance helper for XGBoost (neg/pos)
n_pos = int((y_train == 1).sum())
n_neg = int((y_train == 0).sum())
spw = (n_neg / n_pos) if n_pos else 1.0
print(f"Train class counts: neg={n_neg}, pos={n_pos} => scale_pos_weight≈{spw:.2f}")

cv = StratifiedKFold(n_splits=5, shuffle=True, random_state=42)

def tune_and_eval(model_name, estimator, param_grid, Xtr, Xte):
    grid = GridSearchCV(
        estimator=estimator,
        param_grid=param_grid,
        scoring='roc_auc',
        cv=cv,
        n_jobs=-1,
        refit=True,
    )
    grid.fit(Xtr, y_train)
    best_model = grid.best_estimator_
    y_pred = best_model.predict(Xte)
    y_proba = best_model.predict_proba(Xte)[:, 1]
    test_auc = roc_auc_score(y_test, y_proba)

    # Fold scores for a rough interval (small N -> treat as descriptive)
    fold_scores = [grid.cv_results_[f'split{i}_test_score'][grid.best_index_] for i in range(cv.get_n_splits())]
    cv_mean = float(np.mean(fold_scores))
    cv_std = float(np.std(fold_scores))
    t_crit = 2.776  # ~95% for df=4; descriptive only
    cv_ci_lower = cv_mean - t_crit * (cv_std / np.sqrt(len(fold_scores)))
    cv_ci_upper = min(cv_mean + t_crit * (cv_std / np.sqrt(len(fold_scores))), 1.0)

    print(f"\nTuning {model_name} ...")
    print(f"Best CV ROC-AUC: {cv_mean:.3f} (approx 95%: {cv_ci_lower:.3f}-{cv_ci_upper:.3f})")
    print(f"Test ROC-AUC: {test_auc:.3f}")
    print(f"Best params: {grid.best_params_}")

    return {
        'model': best_model,
        'cv_score': grid.best_score_,
        'cv_score_ci': (cv_ci_lower, cv_ci_upper),
        'test_auc': test_auc,
        'y_pred': y_pred,
        'y_pred_proba': y_proba,
        'best_params': grid.best_params_,
    }

model_results = {}
cv_scores = {}

model_results['Logistic Regression'] = tune_and_eval(
    'Logistic Regression',
    LogisticRegression(
        random_state=42, 
        class_weight='balanced', 
        max_iter=100, 
        solver='liblinear'),
    {'C': [0.1, 1, 10]},
    X_train_scaled,
    X_test_scaled,
 )
cv_scores['Logistic Regression'] = model_results['Logistic Regression']['cv_score']

model_results['Random Forest'] = tune_and_eval(
    'Random Forest',
    RandomForestClassifier(
        random_state=42, 
        bootstrap=True, 
        class_weight='balanced'
    ),
    {
        'n_estimators': [100,200,300],
        'max_depth': [3, 4, 5],
        'min_samples_leaf': [5, 6, 7],
        'max_features': ['sqrt', 'log2'],
    },
    X_train,
    X_test,
 )
cv_scores['Random Forest'] = model_results['Random Forest']['cv_score']

model_results['XGBoost'] = tune_and_eval(
    'XGBoost',
    XGBClassifier(
        random_state=42,
        n_jobs=-1,
    ),
    {
        'scale_pos_weight': [round(spw, 2), 2.0, 1.0],
        'n_estimators': [10, 20, 40],
        'max_depth': [2, 3],
        'learning_rate': [0.001, 0.005, 0.01, 0.03],
        'colsample_bytree': [0.6, 0.8, 1],
        'colsample_bylevel': [0.6, 0.8, 1],
    },
    X_train,
    X_test,
 )
cv_scores['XGBoost'] = model_results['XGBoost']['cv_score']

print("\n=== MODEL COMPARISON ===")
comparison_df = pd.DataFrame({
    'Model': list(cv_scores.keys()),
    'CV_ROC_AUC': [cv_scores[k] for k in cv_scores.keys()],
    'Test_ROC_AUC': [model_results[k]['test_auc'] for k in model_results.keys()],
    'Best_Params': [str(model_results[k]['best_params']) for k in model_results.keys()],
}).sort_values('CV_ROC_AUC', ascending=False)

print(comparison_df.round(3).to_string(index=False))
```

In [ ]:

```
# Model quality visualization dashboard
from sklearn.metrics import precision_recall_curve, average_precision_score
import matplotlib.pyplot as plt
import seaborn as sns
import numpy as np

# 1) ROC curves for all models
plt.figure(figsize=(11, 9))
for name, results in model_results.items():
    fpr, tpr, _ = roc_curve(y_test, results['y_pred_proba'])
    auc_value = results['test_auc']
    plt.plot(fpr, tpr, linewidth=2, label=f"{name} (AUC={auc_value:.3f})")

plt.plot([0, 1], [0, 1], 'k--', alpha=0.6, label='Random classifier')
plt.xlabel('False Positive Rate', fontsize=13)
plt.ylabel('True Positive Rate', fontsize=13)
plt.title('ROC Curves - Model Discrimination', fontsize=15)
plt.legend(loc='lower right', fontsize=12)
plt.grid(alpha=0.3)
plt.tight_layout()
save_plot_png('05_roc_curves.png')
plt.show()

# # 2) Precision-Recall curves (useful for class imbalance)
# plt.figure(figsize=(9, 7))
# baseline = y_test.mean()
# for name, results in model_results.items():
#     precision, recall, _ = precision_recall_curve(y_test, results['y_pred_proba'])
#     ap = average_precision_score(y_test, results['y_pred_proba'])
#     plt.plot(recall, precision, linewidth=2, label=f"{name} (AP={ap:.3f})")

# plt.axhline(y=baseline, color='k', linestyle='--', alpha=0.6, label=f'Baseline={baseline:.3f}')
# plt.xlabel('Recall')
# plt.ylabel('Precision')
# plt.title('Precision-Recall Curves')
# plt.legend(loc='upper right')
# plt.grid(alpha=0.3)
# plt.tight_layout()
# plt.show()

# 3) Confusion matrices for each model
n_models = len(model_results)
fig, axes = plt.subplots(1, n_models, figsize=(6 * n_models, 6))
if n_models == 1:
    axes = [axes]

for ax, (name, results) in zip(axes, model_results.items()):
    cm = confusion_matrix(y_test, results['y_pred'])
    sns.heatmap(
        cm,
        annot=True,
        fmt='d',
        cmap='Blues',
        cbar=False,
        ax=ax,
        xticklabels=['Pred 0', 'Pred 1'],
        yticklabels=['True 0', 'True 1'],
        annot_kws={'fontsize': 12}
    )
    ax.set_title(name, fontsize=13)
    ax.set_xlabel('Predicted label', fontsize=12)
    ax.set_ylabel('True label', fontsize=12)

plt.suptitle('Confusion Matrices by Model', y=1.02, fontsize=15)
plt.tight_layout()
save_plot_png('06_confusion_matrices.png')
plt.show()

# 4) CV vs Test ROC-AUC comparison
plot_df = comparison_df.copy()
plot_df = plot_df.sort_values('CV_ROC_AUC', ascending=False)

x = np.arange(len(plot_df))
width = 0.35

plt.figure(figsize=(11, 8))
plt.bar(x - width/2, plot_df['CV_ROC_AUC'], width=width, label='CV ROC-AUC')
plt.bar(x + width/2, plot_df['Test_ROC_AUC'], width=width, label='Test ROC-AUC')

plt.xticks(x, plot_df['Model'], rotation=20, fontsize=11)
plt.yticks(fontsize=11)
plt.ylim(0, 1)
plt.ylabel('Score', fontsize=13)
plt.title('Cross-Validation vs Test Performance', fontsize=15)
plt.legend(fontsize=12)
plt.grid(axis='y', alpha=0.3)
plt.tight_layout()
save_plot_png('07_cv_vs_test_performance.png')
plt.show()
```

In [ ]:

```
# SHAP comparative analysis for the 3 trained PANS models
import shap
import numpy as np
import pandas as pd
import matplotlib.pyplot as plt
import seaborn as sns

print("=== SHAP COMPARATIVE ANALYSIS (PANS MODELS) ===")

# Keep feature names for all plots/tables
feature_names = list(X.columns)

# IMPORTANT: SHAP must receive inputs in the SAME feature space the model was trained on.
# Logistic Regression was trained on standardized features; tree models were trained on raw features.
X_test_by_model = {
    'Logistic Regression': pd.DataFrame(X_test_scaled, columns=feature_names, index=X_test.index),
    'Random Forest': X_test.copy(),
    'XGBoost': X_test.copy(),
}
X_train_by_model = {
    'Logistic Regression': pd.DataFrame(X_train_scaled, columns=feature_names, index=X_train.index),
    'Random Forest': X_train.copy(),
    'XGBoost': X_train.copy(),
}

# Helper: always return SHAP values for positive class (class 1) with shape (n_samples, n_features)
def get_positive_class_shap(explainer, X_input):
    shap_raw = explainer.shap_values(X_input)

    if isinstance(shap_raw, list):
        # Binary classification may return [class0, class1]
        if len(shap_raw) == 2:
            return np.array(shap_raw[1])
        return np.array(shap_raw[0])

    shap_arr = np.array(shap_raw)
    # Some explainers return (n_samples, n_features, n_classes)
    if shap_arr.ndim == 3:
        return shap_arr[:, :, 1]

    return shap_arr

shap_payload = {}
for model_name, payload in model_results.items():
    model = payload['model']
    X_eval = X_test_by_model[model_name]
    X_bg = X_train_by_model[model_name]

    if model_name == 'Logistic Regression':
        # LinearExplainer: use TRAIN background for stable attributions
        explainer = shap.LinearExplainer(model, X_bg)
    else:
        # TreeExplainer: pass TRAIN background so SHAP uses realistic feature distributions
        explainer = shap.TreeExplainer(model, X_bg)

    shap_values_pos = get_positive_class_shap(explainer, X_eval)
    mean_abs_shap = np.abs(shap_values_pos).mean(axis=0)

    shap_payload[model_name] = {
        'X_eval': X_eval,
        'explainer': explainer,
        'shap_values': shap_values_pos,
        'mean_abs_shap': mean_abs_shap,
    }

# 1) Comparative bar plot: normalize SHAP magnitudes per model before comparing
# (Raw SHAP scales differ across model families; normalization makes the comparison meaningful.)
importance_df = pd.DataFrame({'feature': feature_names})
for model_name in shap_payload:
    importance_df[model_name] = shap_payload[model_name]['mean_abs_shap']

importance_df_norm = importance_df.copy()
for model_name in shap_payload:
    denom = importance_df_norm[model_name].sum()
    if denom > 0:
        importance_df_norm[model_name] = importance_df_norm[model_name] / denom

importance_df_norm['avg_importance'] = importance_df_norm[[
    'Logistic Regression', 'Random Forest', 'XGBoost',
]].mean(axis=1)
importance_df_norm = importance_df_norm.sort_values('avg_importance', ascending=False)

top_n = min(27, len(importance_df_norm))
top_features = importance_df_norm.head(top_n)['feature'].tolist()

plot_df = importance_df_norm.set_index('feature').loc[top_features, [
    'Logistic Regression', 'Random Forest', 'XGBoost',
]]

fig, ax = plt.subplots(figsize=(14, 9))
plot_df.plot(kind='bar', ax=ax, width=0.85)
plt.title('SHAP Feature Importance Comparison Across Models (Top Features; Normalized)', fontsize=15)
plt.ylabel('Normalized mean |SHAP| (per model; sum=1)', fontsize=13)
plt.xlabel('Feature', fontsize=13)
plt.xticks(fontsize=11, rotation=45, ha='right')
plt.yticks(fontsize=11)
plt.grid(axis='y', alpha=0.3)
plt.legend(title='Model', fontsize=11, title_fontsize=12)
plt.tight_layout()
save_plot_png('08_shap_feature_importance_comparison.png')
plt.show()

# 2) Per-model SHAP summary plot (beeswarm) for direct interpretability
for model_idx, (model_name, payload) in enumerate(shap_payload.items()):
    print(f"\n{model_name} - top 27 features by mean |SHAP|:")

    model_importance = pd.DataFrame({
        'feature': feature_names,
        'mean_abs_shap': payload['mean_abs_shap'],
    }).sort_values('mean_abs_shap', ascending=False)
    print(model_importance.head(27).to_string(index=False))

    # Bigger plot size to keep labels readable
    shap.summary_plot(
        payload['shap_values'],
        payload['X_eval'],
        max_display=27,
        plot_size=(12, 8),
        show=False,
    )
    safe_model_name = model_name.replace(' ', '_').lower()
    save_plot_png(f'09_shap_summary_{safe_model_name}.png')
    plt.show()
```

## Model complete¶

In [ ]:

```
df['pandas'] = (df['pans_31_total'] > 36) & (df['aslo_value'] > 200) & (df['sudden_onset'] == 1) & (df['last_strep_infection_episode_months'] < 6)
all_train_columns = [
       'age', 
       'sex', 
       'environment', 
       'family', 
       'source',
                 
       'last_strep_infection_episode_months', 
       'recurrent_infections',
       'aslo_value', 

       'binary_obsessions',
       'binary_compulsions',
       'binary_hoarding',
       'binary_food_refusal',
       'binary_binge_eating',
       'binary_fluid_refusal',
       'binary_motor_tics',
       'binary_vocal_tics',
       'binary_enuresis',

       'penicillin_treatment', 
       'sudden_onset',

       'allergies', 
       'prematurity', 
       'ivf', 
       'functional_digestive_symptoms',
       'bacterial_infections_non_strep', 
       'viral_infections',
       'rheumatic_fever_chorea_endocarditis', 
       'autoimmune_pathology',
       ]
train_columns = all_train_columns
```

In [ ]:

```
# Import additional libraries for advanced analysis
from sklearn.model_selection import train_test_split, cross_val_score, StratifiedKFold
from sklearn.ensemble import RandomForestClassifier, GradientBoostingClassifier
from sklearn.linear_model import LogisticRegression
from sklearn.metrics import classification_report, confusion_matrix, roc_auc_score, roc_curve
from sklearn.preprocessing import StandardScaler, LabelEncoder
from sklearn.decomposition import PCA
from sklearn.cluster import KMeans
from scipy.cluster.hierarchy import dendrogram, linkage, fcluster
from scipy.spatial.distance import squareform
import plotly.express as px
import plotly.graph_objects as go
from plotly.subplots import make_subplots

print("Advanced analysis libraries imported successfully!")

# Prepare data for machine learning
def prepare_ml_data(df, target='pandas'):
    """Prepare data for machine learning by handling categorical variables and missing values"""
    
    # Create a copy
    ml_df = df.copy()
    
    # Remove non-predictive columns
    non_predictive_cols = ['pans_31_total']  # Don't use PANS value to predict PANS binary
    ml_df = ml_df.drop(columns=[col for col in non_predictive_cols if col in ml_df.columns])
    
    # Encode categorical variables
    categorical_features = ml_df.select_dtypes(include=['object']).columns
    le_dict = {}
    
    for col in categorical_features:
        if col != target:
            le = LabelEncoder()
            ml_df[col] = le.fit_transform(ml_df[col].astype(str))
            le_dict[col] = le
    
    # Separate features and target
    X = ml_df.drop(columns=[target])
    # X = X[X.columns[:-1]]
    y = ml_df[target].astype(int)
    
    # Fill any remaining missing values
    X = X.fillna(X.median())
    
    return X, y, le_dict

# Prepare the data
X, y, label_encoders = prepare_ml_data(df[train_columns + ['pandas']])
print(f"Prepared ML dataset: {X.shape[0]} samples, {X.shape[1]} features")
print(f"Target distribution: {y.value_counts().to_dict()}")
```

In [ ]:

```
from xgboost import XGBClassifier
from sklearn.model_selection import GridSearchCV, StratifiedKFold, train_test_split
from sklearn.metrics import roc_auc_score
from sklearn.preprocessing import StandardScaler
from sklearn.linear_model import LogisticRegression
from sklearn.ensemble import RandomForestClassifier
from pathlib import Path
import joblib
import numpy as np
import pandas as pd

# Machine Learning Models for PANS Classification (with Grid Search or cached artifacts)
print("=== MACHINE LEARNING CLASSIFICATION ANALYSIS ===")
print("Training and tuning models to predict PANS status from clinical features\n")

# NOTE (kept minimal for reproducibility + SHAP below):
# - This notebook uses label-encoded categoricals from `prepare_ml_data`.
# - A more 'correct' approach is ColumnTransformer + OneHotEncoder, but that changes feature space and would require updating the SHAP section.

# -----------------------------------------------------------------------------
# Save/Load trained models so you can train once and reuse thereafter
# -----------------------------------------------------------------------------
# By default we load cached fitted models if present; set FORCE_RETRAIN=True to rerun GridSearch.
ARTIFACT_DIR = Path("bazadedatestatistica") / "artifacts"
ARTIFACT_PATH_ALL = ARTIFACT_DIR / "pans_models_all_features.joblib"
ARTIFACT_PATH_SELECTED = ARTIFACT_DIR / "pans_models_selected_features.joblib"

# Backwards-compat alias (some later cells/prints may reference ARTIFACT_PATH)
ARTIFACT_PATH = ARTIFACT_PATH_ALL

LOAD_IF_AVAILABLE = True
FORCE_RETRAIN = False

# Ensure we have a selected-feature list.
# If `selected_train_columns` exists later in the notebook, we reuse it; otherwise derive it.
if 'selected_train_columns' not in globals() or not isinstance(globals().get('selected_train_columns', None), (list, tuple, pd.Index)):
    # Derived to match the earlier "masked sensitivity" definition: remove ASLO/strep recency/sudden onset.
    _drop_for_selected = {'aslo_value', 'last_strep_infection_episode_months', 'sudden_onset'}
    if 'all_train_columns' in globals() and isinstance(all_train_columns, (list, tuple, pd.Index)):
        selected_train_columns = [c for c in list(all_train_columns) if c not in _drop_for_selected]
    else:
        selected_train_columns = [c for c in list(X.columns) if c not in _drop_for_selected]

# Make sure selected columns exist in X (after prepare_ml_data + non-predictive drop)
selected_train_columns = [c for c in list(selected_train_columns) if c in list(X.columns)]


def _get_scaler_feature_names(scaler_obj):
    names = getattr(scaler_obj, 'feature_names_in_', None)
    return list(names) if names is not None else None


def _try_load_model_artifacts(path):
    if not path.exists():
        return None
    try:
        bundle = joblib.load(path)
    except Exception as e:
        print(f"WARNING: Could not load artifacts from {path}: {e}")
        return None

    required_keys = {'feature_names', 'train_index', 'test_index', 'scaler', 'model_results'}
    if not required_keys.issubset(set(bundle.keys())):
        print(f"WARNING: Artifacts missing keys {sorted(required_keys - set(bundle.keys()))}; will retrain.")
        return None

    return bundle


def _validate_bundle(bundle, X_df, y_series):
    saved_features = list(bundle['feature_names'])
    saved_scaler = bundle['scaler']
    saved_scaler_features = bundle.get('scaler_feature_names', None)

    # Feature set must match (ignoring order), but we enforce exact order by reindexing.
    if set(saved_features) != set(X_df.columns):
        print("WARNING: Saved feature set does not match current X columns; will retrain.")
        return False

    # Scaler must have been fit on the saved feature order (when names are available)
    scaler_fit_features = _get_scaler_feature_names(saved_scaler)
    if (saved_scaler_features is not None) and (list(saved_scaler_features) != saved_features):
        print("WARNING: Artifact scaler_feature_names do not match artifact feature_names; cache is inconsistent; will retrain.")
        return False
    if (scaler_fit_features is not None) and (list(scaler_fit_features) != saved_features):
        print("WARNING: Saved scaler was fit on a different feature order/set than artifact feature_names; will retrain.")
        return False

    train_idx = list(bundle['train_index'])
    test_idx = list(bundle['test_index'])
    if (not set(train_idx).issubset(set(X_df.index))) or (not set(test_idx).issubset(set(X_df.index))):
        print("WARNING: Saved train/test indices not found in current dataset index; will retrain.")
        return False
    if (not set(train_idx).issubset(set(y_series.index))) or (not set(test_idx).issubset(set(y_series.index))):
        print("WARNING: Saved train/test indices not found in current target index; will retrain.")
        return False

    return True


def _save_model_artifacts(path, feature_names, scaler_obj, model_results_obj, X_train_df, X_test_df):
    """Persist a self-consistent bundle (models + scaler + exact split indices + feature order)."""
    ARTIFACT_DIR.mkdir(parents=True, exist_ok=True)

    scaler_features = _get_scaler_feature_names(scaler_obj)
    if (scaler_features is not None) and (scaler_features != list(feature_names)):
        raise ValueError(
            "Refusing to save inconsistent artifacts: scaler was fit on a different feature order/set. "
            f"scaler_features={scaler_features}, feature_names={list(feature_names)}"
        )

    bundle = {
        'artifact_version': 3,
        'feature_names': list(feature_names),
        'scaler_feature_names': scaler_features,
        'train_index': list(X_train_df.index),
        'test_index': list(X_test_df.index),
        'scaler': scaler_obj,
        'model_results': model_results_obj,
        'split': {'test_size': 0.3, 'random_state': 42, 'stratify': True},
    }
    joblib.dump(bundle, path)
    print(f"Saved trained model artifacts to: {path}")


def _fit_or_load_suite(feature_set_name, X_df, y_series, artifact_path, prefer_train_idx=None, prefer_test_idx=None):
    """Train or load a model suite for a specific feature space."""
    bundle = None
    if LOAD_IF_AVAILABLE and (not FORCE_RETRAIN):
        bundle = _try_load_model_artifacts(artifact_path)
        if bundle is not None and (not _validate_bundle(bundle, X_df, y_series)):
            bundle = None

    if bundle is not None:
        saved_features = list(bundle['feature_names'])
        X_df = X_df.reindex(columns=saved_features)
        train_idx = list(bundle['train_index'])
        test_idx = list(bundle['test_index'])

        X_train_df = X_df.loc[train_idx].copy()
        X_test_df = X_df.loc[test_idx].copy()
        y_train_s = y_series.loc[train_idx].copy()
        y_test_s = y_series.loc[test_idx].copy()

        scaler_obj = bundle['scaler']
        model_results_obj = bundle['model_results']

        X_train_scaled_arr = scaler_obj.transform(X_train_df[saved_features])
        X_test_scaled_arr = scaler_obj.transform(X_test_df[saved_features])

        print(f"Loaded trained models from cache ({feature_set_name}): {artifact_path}")
        print(f"Training set: {X_train_df.shape[0]} samples")
        print(f"Test set: {X_test_df.shape[0]} samples")

        cv_scores_obj = {k: model_results_obj[k].get('cv_score', np.nan) for k in model_results_obj.keys()}

        return {
            'feature_set_name': feature_set_name,
            'artifact_path': artifact_path,
            'feature_names': saved_features,
            'train_idx': train_idx,
            'test_idx': test_idx,
            'X_train': X_train_df,
            'X_test': X_test_df,
            'y_train': y_train_s,
            'y_test': y_test_s,
            'scaler': scaler_obj,
            'model_results': model_results_obj,
            'cv_scores': cv_scores_obj,
            'X_train_scaled': X_train_scaled_arr,
            'X_test_scaled': X_test_scaled_arr,
        }

    # Train from scratch
    if (prefer_train_idx is not None) and (prefer_test_idx is not None):
        train_idx = list(prefer_train_idx)
        test_idx = list(prefer_test_idx)
    else:
        train_idx, test_idx = train_test_split(
            list(X_df.index),
            test_size=0.3,
            random_state=42,
            stratify=y_series,
        )

    X_train_df = X_df.loc[train_idx].copy()
    X_test_df = X_df.loc[test_idx].copy()
    y_train_s = y_series.loc[train_idx].copy()
    y_test_s = y_series.loc[test_idx].copy()

    print(f"\n--- Training suite: {feature_set_name} ---")
    print(f"Training set: {X_train_df.shape[0]} samples")
    print(f"Test set: {X_test_df.shape[0]} samples")

    # Scale features for logistic regression (trees do not need scaling)
    scaler_obj = StandardScaler()
    X_train_scaled_arr = scaler_obj.fit_transform(X_train_df)
    X_test_scaled_arr = scaler_obj.transform(X_test_df)

    cv = StratifiedKFold(n_splits=5, shuffle=True, random_state=42)

    def tune_and_eval(model_name, estimator, param_grid, Xtr, Xte, ytr, yte):
        grid = GridSearchCV(
            estimator=estimator,
            param_grid=param_grid,
            scoring='roc_auc',
            cv=cv,
            n_jobs=-1,
            refit=True,
        )
        grid.fit(Xtr, ytr)
        best_model = grid.best_estimator_
        y_pred = best_model.predict(Xte)
        y_proba = best_model.predict_proba(Xte)[:, 1]
        test_auc = roc_auc_score(yte, y_proba)

        # Fold scores for a rough interval (small N -> treat as descriptive)
        fold_scores = [grid.cv_results_[f'split{i}_test_score'][grid.best_index_] for i in range(cv.get_n_splits())]
        cv_mean = float(np.mean(fold_scores))
        cv_std = float(np.std(fold_scores))
        t_crit = 2.776  # ~95% for df=4; descriptive only
        cv_ci_lower = cv_mean - t_crit * (cv_std / np.sqrt(len(fold_scores)))
        cv_ci_upper = min(cv_mean + t_crit * (cv_std / np.sqrt(len(fold_scores))), 1.0)

        print(f"\nTuning {model_name} ({feature_set_name}) ...")
        print(f"Best CV ROC-AUC: {cv_mean:.3f} (approx 95%: {cv_ci_lower:.3f}-{cv_ci_upper:.3f})")
        print(f"Test ROC-AUC: {test_auc:.3f}")
        print(f"Best params: {grid.best_params_}")

        return {
            'model': best_model,
            'cv_score': grid.best_score_,
            'cv_score_ci': (cv_ci_lower, cv_ci_upper),
            'test_auc': test_auc,
            'y_pred': y_pred,
            'y_pred_proba': y_proba,
            'best_params': grid.best_params_,
        }

    # Class imbalance helper for XGBoost (neg/pos)
    n_pos = int((y_train_s == 1).sum())
    n_neg = int((y_train_s == 0).sum())
    spw = (n_neg / n_pos) if n_pos else 1.0
    print(f"Train class counts: neg={n_neg}, pos={n_pos} => scale_pos_weight≈{spw:.2f}")

    model_results_obj = {}
    cv_scores_obj = {}

    model_results_obj['Logistic Regression'] = tune_and_eval(
        'Logistic Regression',
        LogisticRegression(
            random_state=42,
            class_weight='balanced',
            max_iter=100,
            solver='liblinear'
        ),
        {'C': [0.1, 1, 10]},
        X_train_scaled_arr,
        X_test_scaled_arr,
        y_train_s,
        y_test_s,
    )
    cv_scores_obj['Logistic Regression'] = model_results_obj['Logistic Regression']['cv_score']

    model_results_obj['Random Forest'] = tune_and_eval(
        'Random Forest',
        RandomForestClassifier(
            random_state=42,
            bootstrap=True,
            class_weight='balanced'
        ),
        {
            'n_estimators': [100, 200, 300],
            'max_depth': [3, 4, 5],
            'min_samples_leaf': [5, 6, 7],
            'max_features': ['sqrt', 'log2'],
        },
        X_train_df,
        X_test_df,
        y_train_s,
        y_test_s,
    )
    cv_scores_obj['Random Forest'] = model_results_obj['Random Forest']['cv_score']

    model_results_obj['XGBoost'] = tune_and_eval(
        'XGBoost',
        XGBClassifier(
            random_state=42,
            n_jobs=-1,
        ),
        {
            'scale_pos_weight': [round(spw, 2), 2.0, 1.0],
            'n_estimators': [10, 20, 40],
            'max_depth': [2, 3],
            'learning_rate': [0.001, 0.005, 0.01, 0.03],
            'colsample_bytree': [0.6, 0.8, 1],
            'colsample_bylevel': [0.6, 0.8, 1],
        },
        X_train_df,
        X_test_df,
        y_train_s,
        y_test_s,
    )
    cv_scores_obj['XGBoost'] = model_results_obj['XGBoost']['cv_score']

    _save_model_artifacts(
        artifact_path,
        feature_names=list(X_train_df.columns),
        scaler_obj=scaler_obj,
        model_results_obj=model_results_obj,
        X_train_df=X_train_df,
        X_test_df=X_test_df,
    )

    return {
        'feature_set_name': feature_set_name,
        'artifact_path': artifact_path,
        'feature_names': list(X_train_df.columns),
        'train_idx': list(X_train_df.index),
        'test_idx': list(X_test_df.index),
        'X_train': X_train_df,
        'X_test': X_test_df,
        'y_train': y_train_s,
        'y_test': y_test_s,
        'scaler': scaler_obj,
        'model_results': model_results_obj,
        'cv_scores': cv_scores_obj,
        'X_train_scaled': X_train_scaled_arr,
        'X_test_scaled': X_test_scaled_arr,
    }


# -----------------------------
# Train / Load BOTH model suites
# -----------------------------
print(f"Feature spaces:\n- all_train_columns (N={X.shape[1]})\n- selected_train_columns (N={len(selected_train_columns)}): {selected_train_columns}")

suite_all = _fit_or_load_suite(
    feature_set_name='all_features',
    X_df=X.copy(),
    y_series=y.copy(),
    artifact_path=ARTIFACT_PATH_ALL,
)

# Use the exact same split indices for the selected-features suite when training from scratch.
X_selected = X.reindex(columns=selected_train_columns).copy()

suite_selected = _fit_or_load_suite(
    feature_set_name='selected_features',
    X_df=X_selected,
    y_series=y.copy(),
    artifact_path=ARTIFACT_PATH_SELECTED,
    prefer_train_idx=suite_all['train_idx'],
    prefer_test_idx=suite_all['test_idx'],
)

# Expose the ALL-FEATURES suite as the notebook's default variables (backwards compatible)
X_train = suite_all['X_train']
X_test = suite_all['X_test']
y_train = suite_all['y_train']
y_test = suite_all['y_test']
scaler = suite_all['scaler']
model_results = suite_all['model_results']
cv_scores = suite_all['cv_scores']
X_train_scaled = suite_all['X_train_scaled']
X_test_scaled = suite_all['X_test_scaled']

# Also expose the SELECTED-FEATURES suite (new variables)
X_train_selected = suite_selected['X_train']
X_test_selected = suite_selected['X_test']
y_train_selected = suite_selected['y_train']
y_test_selected = suite_selected['y_test']
scaler_selected = suite_selected['scaler']
model_results_selected = suite_selected['model_results']
cv_scores_selected = suite_selected['cv_scores']
X_train_scaled_selected = suite_selected['X_train_scaled']
X_test_scaled_selected = suite_selected['X_test_scaled']

print("\n=== MODEL COMPARISON (all_features) ===")
comparison_df = pd.DataFrame({
    'Model': list(cv_scores.keys()),
    'CV_ROC_AUC': [cv_scores[k] for k in cv_scores.keys()],
    'Test_ROC_AUC': [model_results[k].get('test_auc', np.nan) for k in model_results.keys()],
    'Best_Params': [str(model_results[k].get('best_params', None)) for k in model_results.keys()],
}).sort_values('CV_ROC_AUC', ascending=False)

print(comparison_df.round(3).to_string(index=False))

print("\nArtifacts written:")
print(f"- all_features:     {ARTIFACT_PATH_ALL}")
print(f"- selected_features:{ARTIFACT_PATH_SELECTED}")
```

In [ ]:

```
# Model quality visualization dashboard
from sklearn.metrics import precision_recall_curve, average_precision_score
import matplotlib.pyplot as plt
import seaborn as sns
import numpy as np

# 1) ROC curves for all models
plt.figure(figsize=(11, 9))
for name, results in model_results.items():
    fpr, tpr, _ = roc_curve(y_test, results['y_pred_proba'])
    auc_value = results['test_auc']
    plt.plot(fpr, tpr, linewidth=2, label=f"{name} (AUC={auc_value:.3f})")

plt.plot([0, 1], [0, 1], 'k--', alpha=0.6, label='Random classifier')
plt.xlabel('False Positive Rate', fontsize=13)
plt.ylabel('True Positive Rate', fontsize=13)
plt.title('ROC Curves - Model Discrimination', fontsize=15)
plt.legend(loc='lower right', fontsize=12)
plt.grid(alpha=0.3)
plt.tight_layout()
save_plot_png('05_roc_curves.png')
plt.show()

# # 2) Precision-Recall curves (useful for class imbalance)
# plt.figure(figsize=(9, 7))
# baseline = y_test.mean()
# for name, results in model_results.items():
#     precision, recall, _ = precision_recall_curve(y_test, results['y_pred_proba'])
#     ap = average_precision_score(y_test, results['y_pred_proba'])
#     plt.plot(recall, precision, linewidth=2, label=f"{name} (AP={ap:.3f})")

# plt.axhline(y=baseline, color='k', linestyle='--', alpha=0.6, label=f'Baseline={baseline:.3f}')
# plt.xlabel('Recall')
# plt.ylabel('Precision')
# plt.title('Precision-Recall Curves')
# plt.legend(loc='upper right')
# plt.grid(alpha=0.3)
# plt.tight_layout()
# plt.show()

# 3) Confusion matrices for each model
n_models = len(model_results)
fig, axes = plt.subplots(1, n_models, figsize=(6 * n_models, 6))
if n_models == 1:
    axes = [axes]

for ax, (name, results) in zip(axes, model_results.items()):
    cm = confusion_matrix(y_test, results['y_pred'])
    sns.heatmap(
        cm,
        annot=True,
        fmt='d',
        cmap='Blues',
        cbar=False,
        ax=ax,
        xticklabels=['Pred 0', 'Pred 1'],
        yticklabels=['True 0', 'True 1'],
        annot_kws={'fontsize': 12}
    )
    ax.set_title(name, fontsize=13)
    ax.set_xlabel('Predicted label', fontsize=12)
    ax.set_ylabel('True label', fontsize=12)

plt.suptitle('Confusion Matrices by Model', y=1.02, fontsize=15)
plt.tight_layout()
save_plot_png('06_confusion_matrices.png')
plt.show()

# 4) CV vs Test ROC-AUC comparison
plot_df = comparison_df.copy()
plot_df = plot_df.sort_values('CV_ROC_AUC', ascending=False)

x = np.arange(len(plot_df))
width = 0.35

plt.figure(figsize=(11, 8))
plt.bar(x - width/2, plot_df['CV_ROC_AUC'], width=width, label='CV ROC-AUC')
plt.bar(x + width/2, plot_df['Test_ROC_AUC'], width=width, label='Test ROC-AUC')

plt.xticks(x, plot_df['Model'], rotation=20, fontsize=11)
plt.yticks(fontsize=11)
plt.ylim(0, 1)
plt.ylabel('Score', fontsize=13)
plt.title('Cross-Validation vs Test Performance', fontsize=15)
plt.legend(fontsize=12)
plt.grid(axis='y', alpha=0.3)
plt.tight_layout()
save_plot_png('07_cv_vs_test_performance.png')
plt.show()
```

In [ ]:

```
# SHAP comparative analysis for the 3 trained PANS models
import shap
import numpy as np
import pandas as pd
import matplotlib.pyplot as plt
import seaborn as sns

print("=== SHAP COMPARATIVE ANALYSIS (PANS MODELS) ===")

# Keep feature names for all plots/tables
feature_names = list(X.columns)

# IMPORTANT: SHAP must receive inputs in the SAME feature space the model was trained on.
# Logistic Regression was trained on standardized features; tree models were trained on raw features.
X_test_by_model = {
    'Logistic Regression': pd.DataFrame(X_test_scaled, columns=feature_names, index=X_test.index),
    'Random Forest': X_test.copy(),
    'XGBoost': X_test.copy(),
}
X_train_by_model = {
    'Logistic Regression': pd.DataFrame(X_train_scaled, columns=feature_names, index=X_train.index),
    'Random Forest': X_train.copy(),
    'XGBoost': X_train.copy(),
}

# Helper: always return SHAP values for positive class (class 1) with shape (n_samples, n_features)
def get_positive_class_shap(explainer, X_input):
    shap_raw = explainer.shap_values(X_input)

    if isinstance(shap_raw, list):
        # Binary classification may return [class0, class1]
        if len(shap_raw) == 2:
            return np.array(shap_raw[1])
        return np.array(shap_raw[0])

    shap_arr = np.array(shap_raw)
    # Some explainers return (n_samples, n_features, n_classes)
    if shap_arr.ndim == 3:
        return shap_arr[:, :, 1]

    return shap_arr

shap_payload = {}
for model_name, payload in model_results.items():
    model = payload['model']
    X_eval = X_test_by_model[model_name]
    X_bg = X_train_by_model[model_name]

    if model_name == 'Logistic Regression':
        # LinearExplainer: use TRAIN background for stable attributions
        explainer = shap.LinearExplainer(model, X_bg)
    else:
        # TreeExplainer: pass TRAIN background so SHAP uses realistic feature distributions
        explainer = shap.TreeExplainer(model, X_bg)

    shap_values_pos = get_positive_class_shap(explainer, X_eval)
    mean_abs_shap = np.abs(shap_values_pos).mean(axis=0)

    shap_payload[model_name] = {
        'X_eval': X_eval,
        'explainer': explainer,
        'shap_values': shap_values_pos,
        'mean_abs_shap': mean_abs_shap,
    }

# 1) Comparative bar plot: normalize SHAP magnitudes per model before comparing
# (Raw SHAP scales differ across model families; normalization makes the comparison meaningful.)
importance_df = pd.DataFrame({'feature': feature_names})
for model_name in shap_payload:
    importance_df[model_name] = shap_payload[model_name]['mean_abs_shap']

importance_df_norm = importance_df.copy()
for model_name in shap_payload:
    denom = importance_df_norm[model_name].sum()
    if denom > 0:
        importance_df_norm[model_name] = importance_df_norm[model_name] / denom

importance_df_norm['avg_importance'] = importance_df_norm[[
    'Logistic Regression', 'Random Forest', 'XGBoost',
]].mean(axis=1)
importance_df_norm = importance_df_norm.sort_values('avg_importance', ascending=False)

top_n = min(27, len(importance_df_norm))
top_features = importance_df_norm.head(top_n)['feature'].tolist()

plot_df = importance_df_norm.set_index('feature').loc[top_features, [
    'Logistic Regression', 'Random Forest', 'XGBoost',
]]

fig, ax = plt.subplots(figsize=(14, 9))
plot_df.plot(kind='bar', ax=ax, width=0.85)
plt.title('SHAP Feature Importance Comparison Across Models (Top Features; Normalized)', fontsize=15)
plt.ylabel('Normalized mean |SHAP| (per model; sum=1)', fontsize=13)
plt.xlabel('Feature', fontsize=13)
plt.xticks(fontsize=11, rotation=45, ha='right')
plt.yticks(fontsize=11)
plt.grid(axis='y', alpha=0.3)
plt.legend(title='Model', fontsize=11, title_fontsize=12)
plt.tight_layout()
save_plot_png('08_shap_feature_importance_comparison.png')
plt.show()

# 2) Per-model SHAP summary plot (beeswarm) for direct interpretability
for model_idx, (model_name, payload) in enumerate(shap_payload.items()):
    print(f"\n{model_name} - top 27 features by mean |SHAP|:")

    model_importance = pd.DataFrame({
        'feature': feature_names,
        'mean_abs_shap': payload['mean_abs_shap'],
    }).sort_values('mean_abs_shap', ascending=False)
    print(model_importance.head(27).to_string(index=False))

    # Bigger plot size to keep labels readable
    shap.summary_plot(
        payload['shap_values'],
        payload['X_eval'],
        max_display=27,
        plot_size=(12, 8),
        show=False,
    )
    safe_model_name = model_name.replace(' ', '_').lower()
    save_plot_png(f'09_shap_summary_{safe_model_name}.png')
    plt.show()
```

## Sensitivity / specificity test (trained models; all vs selected predictors)¶

This section reports diagnostic **sensitivity** (TPR) and **specificity** (TNR) on the held-out test set for the **already trained** models: **Logistic Regression** (linear), **Random Forest**, and **XGBoost**.

We compare two input scenarios using the same fitted models (no retraining):

- **all\_train\_columns**: predictions using all predictors as trained.
- **selected\_train\_columns**: predictions where non-selected predictors are replaced by their **training-set medians** (so the model is effectively driven only by the selected predictors).

In [ ]:

```
# Sensitivity/Specificity comparison using *existing trained models* (no retraining)
from sklearn.metrics import confusion_matrix, roc_auc_score
import numpy as np
import pandas as pd

def _binary_metrics(y_true, y_pred, y_proba):
    """Return common diagnostic metrics for binary classification (positive class=1)."""
    cm = confusion_matrix(y_true, y_pred, labels=[0, 1])
    if cm.shape != (2, 2):
        return {
            'TN': np.nan, 'FP': np.nan, 'FN': np.nan, 'TP': np.nan,
            'Sensitivity': np.nan,
            'Specificity': np.nan,
            'PPV': np.nan,
            'NPV': np.nan,
            'ROC_AUC': np.nan,
        }
    tn, fp, fn, tp = cm.ravel()
    sensitivity = tp / (tp + fn) if (tp + fn) else np.nan
    specificity = tn / (tn + fp) if (tn + fp) else np.nan
    ppv = tp / (tp + fp) if (tp + fp) else np.nan
    npv = tn / (tn + fn) if (tn + fn) else np.nan
    try:
        auc = roc_auc_score(y_true, y_proba)
    except Exception:
        auc = np.nan
    return {
        'TN': int(tn), 'FP': int(fp), 'FN': int(fn), 'TP': int(tp),
        'Sensitivity': float(sensitivity),
        'Specificity': float(specificity),
        'PPV': float(ppv),
        'NPV': float(npv),
        'ROC_AUC': float(auc),
    }

def _mask_to_selected(X_df, selected_cols, baseline_values):
    """Return X in the same full feature space, but with non-selected cols set to baseline values."""
    selected_cols = set(selected_cols)
    X_masked = X_df.copy()
    cols_to_mask = [c for c in X_masked.columns if c not in selected_cols]
    if cols_to_mask:
        X_masked.loc[:, cols_to_mask] = baseline_values[cols_to_mask].values
    return X_masked

def _get_base_preds_from_payload(payload, y_true_len):
    """Fetch already-computed test predictions from training output (if present)."""
    y_pred = payload.get('y_pred', None)
    y_proba = payload.get('y_pred_proba', None)
    if y_pred is None or y_proba is None:
        return None, None
    y_pred = np.asarray(y_pred)
    y_proba = np.asarray(y_proba)
    if len(y_pred) != y_true_len or len(y_proba) != y_true_len:
        return None, None
    return y_pred, y_proba

# ---- prerequisites from earlier training cells ----
required = ['model_results', 'X_train', 'X_test', 'y_test', 'all_train_columns', 'selected_train_columns']
missing = [k for k in required if k not in globals()]
if missing:
    raise RuntimeError(f"Missing variables from earlier cells: {missing}. Run the model-training cells first.")

print(f"all_train_columns (N={len(all_train_columns)}): {all_train_columns}")
print(f"selected_train_columns (N={len(selected_train_columns)}): {selected_train_columns}")

# Baseline values for masking: medians from training set (keeps realistic 'default' values)
baseline_values = X_train.reindex(columns=all_train_columns).median()

X_test_full = X_test.reindex(columns=all_train_columns).copy()
y_true = y_test.copy()

# Models trained earlier (names must match the training section keys)
model_names = ['Logistic Regression', 'Random Forest', 'XGBoost']
available_models = [m for m in model_names if m in model_results]
if len(available_models) == 0:
    raise RuntimeError(f"None of {model_names} found in model_results.")

rows = []
for model_name in available_models:
    payload = model_results[model_name]
    model = payload['model']
    best_params = payload.get('best_params', None)
    base_pred, base_proba = _get_base_preds_from_payload(payload, y_true_len=len(y_true))

    for feature_set_name, cols_used in [
        ('all_train_columns', all_train_columns),
        ('selected_train_columns', selected_train_columns),
    ]:
        if feature_set_name == 'all_train_columns' and base_pred is not None and base_proba is not None:
            # Use stored predictions from the training/evaluation step (no extra inference).
            y_pred = base_pred
            y_proba = base_proba
        else:
            # Reuse the *same fitted model*; only input X changes (no retraining).
            if feature_set_name == 'all_train_columns':
                X_eval = X_test_full
            else:
                X_eval = _mask_to_selected(X_test_full, cols_used, baseline_values)

            # Apply the original scaler only for Logistic Regression (trained on standardized features).
            if model_name == 'Logistic Regression':
                if 'scaler' not in globals():
                    raise RuntimeError("Missing `scaler` used for Logistic Regression. Re-run the training cell.")
                X_eval_in = scaler.transform(X_eval)
            else:
                X_eval_in = X_eval

            y_pred = model.predict(X_eval_in)
            y_proba = model.predict_proba(X_eval_in)[:, 1]

        m = _binary_metrics(y_true, y_pred, y_proba)
        rows.append({
            'Model': model_name,
            'Feature_Set': feature_set_name,
            'N_features_used': int(len(cols_used)),
            **m,
            'Best_Params': str(best_params),
        })

sensibility_df = pd.DataFrame(rows)

# Add deltas vs all_train_columns per model
sensibility_df['ROC_AUC_delta_vs_all'] = np.nan
sensibility_df['Sensitivity_delta_vs_all'] = np.nan
sensibility_df['Specificity_delta_vs_all'] = np.nan
for mname in sensibility_df['Model'].unique():
    base = sensibility_df[(sensibility_df['Model'] == mname) & (sensibility_df['Feature_Set'] == 'all_train_columns')]
    if len(base) != 1:
        continue
    base = base.iloc[0]
    idx = sensibility_df['Model'].eq(mname)
    sensibility_df.loc[idx, 'ROC_AUC_delta_vs_all'] = sensibility_df.loc[idx, 'ROC_AUC'] - base['ROC_AUC']
    sensibility_df.loc[idx, 'Sensitivity_delta_vs_all'] = sensibility_df.loc[idx, 'Sensitivity'] - base['Sensitivity']
    sensibility_df.loc[idx, 'Specificity_delta_vs_all'] = sensibility_df.loc[idx, 'Specificity'] - base['Specificity']

sensibility_df = sensibility_df.sort_values(['Model', 'Feature_Set']).reset_index(drop=True)

print("\nSensitivity/Specificity results (test set; no retraining):")
display(sensibility_df.round(3))
```

## Interpretation (sensitivity analysis; replacing the columns in the complete model)¶

This table compares diagnostic performance on the same held-out test set for the same **already-fitted** models under two input scenarios:

- **all\_train\_columns**: the model receives the full feature set it was trained on.
- **selected\_train\_columns**: non-selected predictors are *masked* by replacing them with their **training-set medians**. This isolates how much the model’s predictions rely on the selected predictors *while keeping the fitted model fixed* (i.e., this is **not** a refit/retrained reduced model).

**How to read the metrics**

- **Sensitivity (TPR)**: among true positives, how many the model detects (lower FN).
- **Specificity (TNR)**: among true negatives, how many the model correctly rules out (lower FP).
- **PPV / NPV**: predictive values, which depend on the class prevalence in this dataset.
- **ROC\_AUC**: threshold-independent discrimination (higher is better).
- **Δ vs all** columns: change in each metric for the masked-input scenario relative to the full-input scenario *for the same model*. Negative deltas indicate performance loss when non-selected predictors are “neutralized”.

**Practical interpretation**

- If **ROC\_AUC and Sensitivity/Specificity remain similar** after masking, the selected predictors carry most of the predictive signal.
- If **performance drops materially** (especially ROC\_AUC), the model is leveraging information from the masked predictors and the selected set may be incomplete for discrimination.
- Note: predictions use each model’s default classification threshold (typically 0.5). If clinical use requires prioritizing sensitivity (screening) or specificity (rule-in), the operating threshold should be tuned accordingly.

**Caution**

Because this cohort is relatively small, these values should be treated as **descriptive** and may vary with different train/test splits. The masking approach also preserves the model’s learned interactions from training, so it answers “how dependent is this fitted model on these predictors?” rather than “how would a reduced model trained only on these predictors perform?”

## Parsimonious model performance¶

This section answers: **“If we commit to a reduced predictor set and refit, how well can we do?”**

- **All-features model suite**: trained on all predictors in `X` (the full feature space used above).
- **Parsimonious (selected-features) model suite**: trained *from scratch* using only `selected_train_columns` (a true reduced model, not masking).

Both suites use the **same train/test split indices** to make the comparison fair.

In [ ]:

```
import numpy as np
import pandas as pd
from sklearn.metrics import roc_auc_score, confusion_matrix

# Guardrails
required = ['model_results', 'model_results_selected', 'selected_train_columns', 'X_train', 'X_train_selected', 'y_test', 'y_test_selected']
missing = [k for k in required if k not in globals()]
if missing:
    raise RuntimeError(f"Missing required variables (run the training cell first): {missing}")

# Ensure split comparability (we force the same indices during training; this checks it stayed true)
if not (set(y_test.index) == set(y_test_selected.index)):
    print("WARNING: y_test indices differ between suites; comparison may not be apples-to-apples.")


def _binary_metrics(y_true, y_pred, y_proba):
    tn, fp, fn, tp = confusion_matrix(y_true, y_pred, labels=[0, 1]).ravel()
    sensitivity = tp / (tp + fn) if (tp + fn) else np.nan
    specificity = tn / (tn + fp) if (tn + fp) else np.nan
    ppv = tp / (tp + fp) if (tp + fp) else np.nan
    npv = tn / (tn + fn) if (tn + fn) else np.nan
    auc = roc_auc_score(y_true, y_proba) if (y_proba is not None and len(np.unique(y_true)) == 2) else np.nan
    return {
        'TN': int(tn), 'FP': int(fp), 'FN': int(fn), 'TP': int(tp),
        'Sensitivity': sensitivity,
        'Specificity': specificity,
        'PPV': ppv,
        'NPV': npv,
        'ROC_AUC': auc,
    }


def _collect_suite_rows(feature_set, model_results_dict, y_true, n_features):
    rows = []
    for model_name, payload in model_results_dict.items():
        y_pred = payload.get('y_pred', None)
        y_proba = payload.get('y_pred_proba', None)
        if y_pred is None or y_proba is None:
            continue

        m = _binary_metrics(y_true=y_true, y_pred=y_pred, y_proba=y_proba)
        rows.append({
            'Model': model_name,
            'Feature_Set': feature_set,
            'N_Features': int(n_features),
            'CV_ROC_AUC': float(payload.get('cv_score', np.nan)),
            'Test_ROC_AUC': float(payload.get('test_auc', m['ROC_AUC'])),
            'Sensitivity': float(m['Sensitivity']),
            'Specificity': float(m['Specificity']),
            'PPV': float(m['PPV']),
            'NPV': float(m['NPV']),
            'Best_Params': str(payload.get('best_params', None)),
        })

    return rows


all_rows = []
all_rows += _collect_suite_rows(
    feature_set='all_features',
    model_results_dict=model_results,
    y_true=y_test,
    n_features=X_train.shape[1],
)
all_rows += _collect_suite_rows(
    feature_set='selected_features_refit',
    model_results_dict=model_results_selected,
    y_true=y_test_selected,
    n_features=X_train_selected.shape[1],
)

parsimonious_df = pd.DataFrame(all_rows)

# Align and compute deltas (selected - all) per model
wide = parsimonious_df.pivot(index='Model', columns='Feature_Set', values=['CV_ROC_AUC', 'Test_ROC_AUC', 'Sensitivity', 'Specificity', 'PPV', 'NPV', 'N_Features'])
wide.columns = [f"{metric}__{fs}" for metric, fs in wide.columns]
wide = wide.reset_index()

for metric in ['CV_ROC_AUC', 'Test_ROC_AUC', 'Sensitivity', 'Specificity', 'PPV', 'NPV']:
    a = f"{metric}__all_features"
    b = f"{metric}__selected_features_refit"
    if a in wide.columns and b in wide.columns:
        wide[f"Δ_{metric} (selected - all)"] = wide[b] - wide[a]

# Rank by all-features test AUC for readability
sort_col = 'Test_ROC_AUC__all_features' if 'Test_ROC_AUC__all_features' in wide.columns else None
if sort_col:
    wide = wide.sort_values(sort_col, ascending=False)

print("Parsimonious (refit) vs all-features performance on the held-out test set")
display(wide.round(3))

# Quick single-line summary of best models
try:
    best_all = parsimonious_df[parsimonious_df['Feature_Set'] == 'all_features'].sort_values('Test_ROC_AUC', ascending=False).head(1)
    best_sel = parsimonious_df[parsimonious_df['Feature_Set'] == 'selected_features_refit'].sort_values('Test_ROC_AUC', ascending=False).head(1)
    if len(best_all) and len(best_sel):
        print(
            f"Best all-features by Test ROC-AUC: {best_all.iloc[0]['Model']} ({best_all.iloc[0]['Test_ROC_AUC']:.3f}) | "
            f"Best selected-features refit: {best_sel.iloc[0]['Model']} ({best_sel.iloc[0]['Test_ROC_AUC']:.3f})"
        )
except Exception:
    pass
```
