# Supplementary material for: Interpretable Modelling of a Streptococcal Exposure–Associated Neuropsychiatric Profile in a Pediatric Cohort
Source: Children (Basel). 2026 Oct 21;13(10):1443. doi: 10.3390/children13101443 (PMC13703277; doi:10.3390/children13101443)
Supplement: Supplementary file 1 [file children-13-01443-s001.zip › S2. Supplementary_Materials_AI_Model.pdf]

Supplementary Materials

Interpretable Modelling of a Streptococcal-Exposure–Associated Neuropsychiatric Profile in a Pediatric Cohort

The present modelling study uses data from a broader baseline observational cohort. The source-cohort phase provided the descriptive, serological, clinical, and psychometric context, whereas the present analysis addresses a separate secondary question using interpretable supervised modelling. Key cohort characteristics and descriptive findings relevant to endpoint construction and the rationale for the modelling feature sets are summarized below.

Table S1. Source-cohort construction, retention, and analytical subgroup structure.

Item	Value	Interpretive note
Initial screened cases	154	Children/adolescents screened through the source clinical workflow.
Final analytical cohort	96	Participants retained with analyzable baseline data.
Total not retained	58	Not entered into the modelling or descriptive analytical cohort.
Confounding neurological/developmental/organic/medical conditions	24	Excluded because phenotype interpretation was substantially confounded.
Incomplete core data	18	Not retained because core variables required for analysis were unavailable.
Withdrew or did not complete assessment	16	Assessment process was not completed.
Elevated ASO stratum	50	ASO >200 IU/mL.
Non-elevated ASO stratum	46	ASO ≤200 IU/mL.
PANS-31 >36	34	Pragmatic higher symptom-burden subgroup used descriptively and for endpoint construction.
PANS-31 ≤36	62	Lower symptom-burden subgroup.
Operational modelling endpoint-positive	24	PANS-31 >36 AND ASO >200 IU/mL AND abrupt onset/worsening AND infection recency <6 months.
Operational modelling endpoint-negative	72	All remaining participants.
Training set	67	Stratified 70/30 split; 17 endpoint-positive and 50 endpoint-negative.
Held-out test set	29	Stratified 70/30 split; 7 endpoint-positive and 22 endpoint-negative.

Note: The operational modelling endpoint was constructed after source-cohort harmonization and was not treated as physician-confirmed PANDAS.

Table S2. Relationship between the descriptive observational phase and the present supervised modelling phase.

Element	Descriptive observational phase	Present modelling phase
Source pool	154 screened children/adolescents	Same source pool; modelling used retained analytical cohort only
Analytical cohort	96 retained participants	Same 96 participants
Purpose	Describe ASO, PANS-31, infection recency, EEG, developmental/systemic variables, and ASI/CSI convergence	Internally reconstruct an operational streptococcal-exposure–associated clinical–serological profile
Main endpoint/outcome	ASO value, PANS-31 total score, descriptive strata, psychometric convergence	Binary operational endpoint: PANS-31 >36 + ASO >200 + abrupt onset/worsening + infection recency <6 months
Modelling role	Contextualized clinical and psychometric structure of the source cohort	Supervised internal modelling with full-feature and restricted-feature settings
Interpretive boundary	Descriptive and hypothesis-generating; no causal inference or biomarker validation	Internal profile reconstruction; no independent diagnostic prediction or clinical deployment

Note: The present manuscript is self-contained. The descriptive observational phase is summarized only to document the source-cohort structure and modelling rationale.

Table S3. Key descriptive distributions from the source-cohort observational phase.

Comparison	Variable	Higher / exposed group	Lower / reference group	Interpretive role
ASO >200 vs ASO ≤200	Age, years	12 [11–13]	14 [10–15]	Source-cohort context
ASO >200 vs ASO ≤200	ASO value, IU/mL	648 [527–764]	116 [74–160]	Serological separation
ASO >200 vs ASO ≤200	Time since last streptococcal infection, months	3 [2–5]	12 [12–12]	Temporal-exposure separation
ASO >200 vs ASO ≤200	PANS-31 total score	40 [34–50]	22 [18–25]	Symptom-burden separation
ASO >200 vs ASO ≤200	Abrupt onset/worsening	43/50 (86.0%)	5/46 (10.9%)	Onset-pattern alignment
ASO >200 vs ASO ≤200	Prematurity	19/50 (38.0%)	2/46 (4.3%)	Developmental/contextual signal
ASO >200 vs ASO ≤200	Recurrent infections	15/50 (30.0%)	7/46 (15.2%)	Broader infection-history context
ASO >200 vs ASO ≤200	Functional digestive symptoms, any	37/50 (74.0%)	6/46 (13.0%)	Systemic/somatic context
PANS-31 >36 vs ≤36	Age, years	12 [11–14]	14 [10–15]	Source-cohort context
PANS-31 >36 vs ≤36	ASO value, IU/mL	674 [549–788]	150 [90–283]	Serological alignment with higher burden
PANS-31 >36 vs ≤36	Time since last streptococcal infection, months	3 [2–4]	12 [6–12]	Temporal alignment with higher burden
PANS-31 >36 vs ≤36	PANS-31 total score	45 [40–54]	24 [20–30]	Symptom-burden separation

Comparison	Variable	Higher / exposed group	Lower / reference group	Interpretive role
PANS-31 >36 vs ≤36	Abrupt onset/worsening	27/34 (79.4%)	21/62 (33.9%)	Endpoint component support
PANS-31 >36 vs ≤36	Prematurity	14/34 (41.2%)	7/62 (11.3%)	Developmental/contextual signal
PANS-31 >36 vs ≤36	IVF history	8/34 (23.5%)	4/62 (6.5%)	Developmental/contextual signal
PANS-31 >36 vs ≤36	Recurrent infections	12/34 (35.3%)	10/62 (16.1%)	Broader infection-history context
PANS-31 >36 vs ≤36	Functional digestive symptoms, any	25/34 (73.5%)	18/62 (29.0%)	Systemic/somatic context

Note: Values are medians [IQR] or counts/denominators (%). Values coded as 12 months include intervals of 12 months or longer.

Table S3A. Stratified distribution of recorded non-streptococcal bacterial and viral infection histories in the source cohort.

Infection-history category	Non-elevated ASO, n = 46	Elevated ASO, n = 50	Overall, N = 96	Representation
Non-streptococcal bacterial infections	4/46 (8.7%); *Clostridium* spp., n = 2; *Borrelia* spp., n = 1; MRSE, n = 1	3/50 (6.0%): *Clostridium* spp., n = 3	7/96 (7.3%)	Binary presence/absence
Viral infections	5/46 (10.9%); CMV, n = 3; EBV, n = 2	2/50 (4.0%): CMV, n = 2	7/96 (7.3%)	Binary presence/absence

Note: Etiological labels reflect the information recorded in the source clinical database. MRSE, methicillin-resistant *Staphylococcus epidermidis*; CMV, cytomegalovirus; EBV, Epstein–Barr virus. Because individual etiological categories were sparsely represented, non-streptococcal bacterial and viral infection histories were encoded as binary presence/absence variables for supervised modelling.

Table S4. Key exploratory statistical signals supporting the operational clinical–serological endpoint rationale.

Signal	Test	Statistic / effect	p-value	Interpretive role
ASO value vs PANS-31 total score	Spearman correlation	$\varrho = 0.806$; n = 96;	3.68×10^{-23}	Strong serological–symptom-burden alignment
Time since last streptococcal infection vs ASO value	Spearman correlation	$\varrho = -0.787$; n = 96	1.89×10^{-21}	Shorter reported interval associated with higher ASO
Time since last streptococcal infection vs PANS-31 total score	Spearman correlation	$\varrho = -0.768$; n = 96	7.34×10^{-20}	Shorter reported interval associated with higher symptom burden
Age vs ASO value	Spearman correlation	$\varrho = -0.239$	0.019	Modest age-related context
Age vs PANS-31 total score	Spearman correlation	$\varrho = -0.251$	0.014	Modest age-related context
Sex vs ASO value	Mann–Whitney U	Median ASO: boys 526 IU/mL vs girls 174 IU/mL	0.00807	Descriptive demographic context

Signal	Test	Statistic / effect	p-value	Interpretive role
Sex vs PANS-31 total score	Mann–Whitney U	Median PANS-31: boys 33 vs girls 23	0.00177	Descriptive demographic context
Abrupt onset/worsening vs ASO value	Mann–Whitney U	Median ASO: 622.5 vs 133.5 IU/mL	1.39×10^{-9}	Supports onset-related component
Abrupt onset/worsening vs PANS-31 total score	Mann–Whitney U	Median PANS-31: 38 vs 23	3.40×10^{-7}	Supports onset-related component
Functional digestive symptoms vs ASO value	Kruskal–Wallis test	H = 28.38	6.89×10^{-7}	Systemic/somatic contextual signal
Functional digestive symptoms vs PANS-31 total score	Kruskal–Wallis test	H = 24.72	4.30×10^{-6}	Systemic/somatic contextual signal
Prematurity vs ASO value	Mann–Whitney U	Median ASO: 652 vs 178 IU/mL	5.12×10^{-4}	Developmental contextual signal
Prematurity vs PANS-31 total score	Mann–Whitney U	Median PANS-31: 40 vs 26	3.58×10^{-5}	Developmental contextual signal
IVF history vs ASO value	Mann–Whitney U	Tendency toward higher ASO	0.106	Not retained as a central serological signal
IVF history vs PANS-31 total score	Mann–Whitney U	Median PANS-31: 42 vs 29.5	0.00793	Developmental/contextual signal
Recurrent infections vs ASO value	Mann–Whitney U	Median ASO: 657 vs 193.5 IU/mL	0.00467	Broader infection-history context
Recurrent infections vs PANS-31 total score	Mann–Whitney U	Median PANS-31: 40.5 vs 29.5	0.0267	Broader infection-history context

Note: These statistics summarize the source-cohort descriptive workflow. They were not used as automatic variable-selection procedures and were not interpreted as causal or diagnostic-validation tests.

Table S5. PANS-31 psychometric and PANS–ASI/CSI convergence summary from the source-cohort descriptive workflow.

Analysis component	Result	Interpretive note
PANS-31 total internal consistency	Cronbach's $\alpha = 0.835$	Good total-scale reliability in the source cohort.
Domain-level factor structure	4 components; cumulative variance = 72.7%; KMO = 0.766	Supports structured dimensional organization at domain level.
Full PANS × ASI/CSI matrix	43/121 correlations significant after FDR correction; 9/121 correlations with $\lvert r \rvert > 0.5$; range −0.407 to 0.899	Supports domain-level convergence without diagnostic equivalence.
Theoretical domain correspondences	Anxiety r = 0.899; eating r = 0.801; conduct/behavioral r = 0.765; OCD r = 0.719; motor r = 0.693; enuresis/urinary r = 0.684; psychotic r = 0.626; vocal r = 0.550; affective/mood r = 0.464	Strongest matched domain pairs supported PANS-31 as a dimensional symptom-burden measure.
Domain correspondence pattern	Nine ASI/CSI domains had prespecified theoretical counterparts within PANS-31; eight of these nine pairings showed correlations of $\lvert r \rvert \geq 0.50$, while the affective/mood pairing showed a moderate correlation (r = 0.464). Two	Supports structured domain-level convergence across mapped constructs, with one comparatively weaker but directionally consistent correspondence; two ASI/CSI domains were intentionally unmapped because no direct PANS-31 equivalent

Analysis component	Result	Interpretive note
	additional ASI/CSI domains—addictions and pervasive developmental symptoms—had no direct PANS-31 counterpart and were therefore not included in the primary theoretical mapping	was available
MTMM summary	Mean convergent |r| = 0.689; ASI/CSI within-scale discriminant mean |r| = 0.126; PANS within-scale discriminant mean |r| = 0.301; heterotrait–heteromethod mean |r| = 0.186	Convergent associations exceeded discriminant associations.
Binary classification agreement	Accuracy = 53.1%; Cohen's κ = 0.062; 95% CI: −0.20 to 0.32	PANS-31 and pooled ASI/CSI totals are not interchangeable binary classifiers.

Note: PANS–ASI/CSI analyses supported use of PANS-31 as a dimensional symptom-burden measure in this cohort, but did not validate PANS-31 >36 as an external diagnostic cutoff.

Table S6. Correlations between individual ASI/CSI domains and PANS-31 total score.

ASI/CSI domain	Correlation with PANS-31 total	p-value	Interpretive note
Enuresis	r = 0.581	<0.001	Strongest positive association with total burden.
Anxiety	r = 0.566	<0.001	Strong positive association with total burden.
Eating-related symptoms	r = 0.379	0.001	Moderate positive association.
Motor symptoms	r = 0.328	0.003	Moderate positive association.
Conduct symptoms	r = 0.287	0.008	Weak-to-moderate positive association.
CSI pervasive domain	r = −0.310	0.005	Moderate inverse association in the source cohort.
OCD domain	r = 0.215	0.055	Borderline association.
Addictions	r = −0.057	0.583	Not significant.
Vocal symptoms	r = −0.127	0.241	Not significant.
Psychotic symptoms	r = −0.188	0.083	Not significant.
Affective symptoms	r = −0.187	0.083	Not significant.

Note: Correlations are shown to document dimensional convergence and divergence. ASI/CSI domains were not used as final modelling predictors.

Table S7. Ancillary EEG source-cohort signals not carried into the final modelling matrices.

EEG descriptor	Association with ASO value	Association with PANS-31 total score	Interpretive note

EEG descriptor	Association with ASO value	Association with PANS-31 total score	Interpretive note
Low-voltage alpha activity in temporo-parieto-occipital regions	$p < 0.001$	$p < 0.001$	Ancillary neurophysiological context; not retained in final modelling matrices.
Dominant alpha activity in temporo-parieto-occipital regions	$p < 0.001$	$p < 0.001$	Ancillary neurophysiological context; not retained in final modelling matrices.
Paroxysmal theta activity	$p < 0.001$	$p < 0.001$	Ancillary neurophysiological context; not retained in final modelling matrices.
Spike–wave complexes	$p < 0.001$	$p < 0.001$	Ancillary neurophysiological context; not retained in final modelling matrices.
Polymorphic theta activity	$p = 0.088$	$p = 0.026$	Tracked symptom burden more than serological status.
Nonspecific EEG changes	$p = 0.425$	$p = 0.048$	Weak/nonspecific source-cohort descriptor.

Note: EEG variables contributed to broader cohort characterization but were not retained in the final supervised modelling matrices to reduce dimensionality and preserve interpretability.

Table S8. Extended variable dictionary and analytical handling across the observational and modelling workflow.

Variable or group	Clinical meaning / role	Used before modelling	Candidate predictor	Full-feature	Restricted-feature	Rationale
age						
	Age in completed years at the baseline psychiatric assessment; continuous numeric variable.	Yes	Yes	Yes	Yes	Continuous demographic predictor.
sex	Sex recorded in the source dataset (0 = male; 1 = female); categorical demographic variable.	Yes	Yes	Yes	Yes	Demographic heterogeneity variable; encoded for modelling.
environment	Usual residential environment (0 = urban; 1 = rural); contextual category, not an index of socioeconomic status.	Yes	Yes	Yes	Yes	Contextual predictor retained after encoding.
family	Living/care arrangement (0 = living with family; 1 = foster care); recorded family-setting category, not a family-functioning score.	Yes	Yes	Yes	Yes	Clinical-context variable.
source	Psychiatric care setting (0 = hospital-based assessment; 1 = ambulatory/outpatient assessment).	Yes	Yes	Yes	Yes	Care-pathway context.

Variable or group	Clinical meaning / role	Used before modelling	Candidate predictor	Full-feature	Restricted-feature	Rationale
last_strep_infection_episode_months	Reported interval, in months, since the last clinically plausible streptococcal episode; values recorded as >12 months were capped at 12 for analysis. The timing was not systematically microbiologically confirmed.	Yes	Yes	Yes	No	Core temporal-exposure variable; excluded from restricted-feature analysis as endpoint-proximal.
recurrent_infections	Caregiver- or record-supported history of recurrent infections (0 = absent; 1 = present); distinct from time since the last reported streptococcal episode.	Yes	Yes	Yes	Yes	Broader infection-history predictor, distinct from recent streptococcal infection interval.
aslo_value	Measured antistreptolysin O (ASO) titer in IU/mL, analysed as a continuous numeric laboratory variable.	Yes	Yes	Yes	No	Principal serological predictor; excluded from restricted-feature analysis as endpoint-proximal.
aslo_binary / ASO category	Source ASO category and derived elevated-ASO indicator (0 = ASO ≤200 IU/mL; 1 = ASO >200 IU/mL for the binary indicator). These were used for cohort stratification and endpoint definition, not as ordinary model predictors.	Yes	Reviewed	No	No	Used for cohort structure and endpoint construction; excluded as ordinary predictor.
previous_aslo_test_modified	History of a previously recorded abnormal/modified ASO test (source field coded 0 = no; 1 = yes); a historical clinical descriptor, not the baseline continuous ASO titer.	Reviewed	No	No	No	Not retained in final analytical matrices.
penicillin_treatment	Recorded history of penicillin treatment (0 = absent; 1 = present); treatment-exposure information, not a measure of treatment efficacy or causal response.	Yes	Yes	Yes	Yes	Treatment-history variable available at baseline.
sudden_onset	Sudden emergence of new neuropsychiatric symptoms or clear worsening of pre-existing manifestations occurring approximately within the week preceding baseline psychiatric assessment, based on	Yes	Yes	Yes	No	Original clinical-history variable included in the operational composite endpoint; retained in the full-feature model and excluded from the restricted-feature model

Variable or group	Clinical meaning / role	Used before modelling	Candidate predictor	Full-feature	Restricted-feature	Rationale
	caregiver-reported history and available clinical documentation (0 = criterion not documented; 1 = criterion documented)					because of its direct relationship with endpoint construction.
pans_31_total	Sum of 31 PANS-31 items, each scored 0–4 (theoretical total 0–124); continuous dimensional neuropsychiatric symptom-burden measure.	Yes	No	No	No	Used descriptively and for endpoint construction; excluded because endpoint includes PANS-31 >36.
pans_binary	Derived higher-burden indicator (0 = PANS-31 total ≤36; 1 = PANS-31 total >36); an internal operational threshold, not a validated diagnostic cutoff.	Yes	No	No	No	Endpoint component; not used as an ordinary predictor.
operational_endpoint	Binary composite target (1 only if PANS-31 >36, ASO >200 IU/mL, abrupt onset/worsening, and <6 months since the last reported streptococcal episode all coexist; otherwise 0). Not a PANS/PANDAS diagnosis.	Target only	Target only	Target	Target	Defined as simultaneous PANS-31 >36, ASO >200, sudden_onset = 1, and infection interval <6 months.
binary_obsessions	PANS-31 obsessions item recoded as 0 for item score ≤2 and 1 for high-intensity score >2 (original item scale 0–4).	Yes	Yes	Yes	Yes	Selected PANS-31 item; score >2.
binary_compulsions	PANS-31 compulsions item recoded as 0 for item score ≤2 and 1 for high-intensity score >2 (original item scale 0–4).	Yes	Yes	Yes	Yes	Selected PANS-31 item; score >2.
binary_hoarding	PANS-31 hoarding item recoded as 0 for item score ≤2 and 1 for high-intensity score >2 (original item scale 0–4).	Yes	Yes	Yes	Yes	Selected PANS-31 item; score >2.
binary_food_refusal	PANS-31 food refusal/restricted intake item recoded as 0 for item score ≤2 and 1 for high-intensity score >2 (original item scale 0–4).	Yes	Yes	Yes	Yes	Restrictive intake/eating-disturbance feature; score >2.
binary_binge_eating	PANS-31 binge-eating item recoded as 0 for item score ≤2 and 1 for high-intensity score >2 (original item scale 0–4).	Yes	Yes	Yes	Yes	Eating-dysregulation indicator; score >2.

Variable or group	Clinical meaning / role	Used before modelling	Candidate predictor	Full-feature	Restricted-feature	Rationale
binary_fluid_refusal	PANS-31 fluid refusal item recoded as 0 for item score ≤2 and 1 for high-intensity score >2 (original item scale 0–4).	Yes	Yes	Yes	Yes	Restrictive fluid-intake feature; score >2.
binary_motor_tics	PANS-31 motor tics item recoded as 0 for item score ≤2 and 1 for high-intensity score >2 (original item scale 0–4).	Yes	Yes	Yes	Yes	Tic-domain feature; score >2.
binary_vocal_tics	PANS-31 vocal tics item recoded as 0 for item score ≤2 and 1 for high-intensity score >2 (original item scale 0–4).	Yes	Yes	Yes	Yes	Tic-domain feature; score >2.
binary_enuresis	PANS-31 enuresis item recoded as 0 for item score ≤2 and 1 for high-intensity score >2 (original item scale 0–4).	Yes	Yes	Yes	Yes	Urinary-domain variable; score >2.
binary_urinary_frequency	PANS-31 increased urinary frequency item considered as a potential high-intensity indicator (>2 on the 0–4 item scale); not selected for either final model.	Reviewed	No	No	No	Reviewed but not retained in final matrices.
binary_hallucinations / binary_delirium	PANS-31 hallucination and delirium-like symptom items considered as potential high-intensity indicators (>2 on the 0–4 item scale); neither retained in final models.	Reviewed	No	No	No	Reviewed but not retained in final matrices to reduce dimensionality.
Other PANS-31 items	Remaining PANS-31 symptom items, each recorded on the original 0–4 scale; available for dimensional clinical characterization but not included as individual predictors.	Yes	Reviewed	No	No	Not retained individually to reduce dimensionality and excessive circularity with PANS-31 burden.
ASI/CSI domain scores	Age-appropriate Child Symptom Inventory/Adolescent Symptom Inventory domain measures covering psychiatric symptom clusters; used for descriptive and psychometric convergence analyses only.	Yes	Exploratory only	No	No	Used for broader observational characterization and convergence with PANS; not retained in model.
EEG descriptors	EEG findings derived from routine clinical reports (global	Yes	Exploratory only	No	No	Used for source-cohort characterization only.

Variable or group	Clinical meaning / role	Used before modelling	Candidate predictor	Full-feature	Restricted-feature	Rationale
	EEG assessment and specified rhythm, theta, spike–wave, and nonspecific descriptors); used for observational characterization only.					
iq	IQ-related categorical information recorded at baseline (source-derived category rather than a continuous model predictor); not used in the supervised feature matrices.	Yes	Exploratory only	No	No	Not retained in final matrices.
allergies	History of allergic/atopic conditions, originally recorded using numerical diagnostic identifiers and recoded for supervised modelling as a binary presence/absence indicator (0 = absent; 1 = present). Original diagnostic identifiers were not interpreted as ordinal measurements or severity scores.	Yes	Yes	Yes	Yes	Developmental/systemic background variable.
prematurity	Recorded history of premature birth (0 = absent; 1 = present); developmental/perinatal antecedent.	Yes	Yes	Yes	Yes	Developmental vulnerability marker.
ivf	History of conception through in vitro fertilization (0 = absent; 1 = present); perinatal background characteristic.	Yes	Yes	Yes	Yes	Perinatal/developmental context.
functional_digestive_symptoms	Count category for recorded functional digestive symptoms (0 = none; 1 = one symptom; 2 = two or more symptoms); three-level ordinal source variable, not a validated gastrointestinal severity score.	Yes	Yes	Yes	Yes	Systemic/somatic contextual variable.
bacterial_infections_non_strep	Recorded history of non-streptococcal bacterial infections, aggregated as 0 = absent and 1 = present; individual etiological categories were too sparse for separate modelling.	Yes	Yes	Yes	Yes	Non-streptococcal infectious context.

Variable or group	Clinical meaning / role	Used before modelling	Candidate predictor	Full-feature	Restricted-feature	Rationale
viral_infections	Recorded history of viral infections, aggregated as 0 = absent and 1 = present; individual etiological categories were too sparse for separate modelling.	Yes	Yes	Yes	Yes	Viral infectious context.
rheumatic_fever_chorea_endocarditis	History compatible with rheumatic fever, chorea, or endocarditis (0 = absent; 1 = present). All 96 analysed participants had value 0; consequently, this retained source feature had zero variance and no discriminative contribution.	Yes	Yes	Yes	Yes	Clinical-history variable related to the post-streptococcal disease spectrum. No positive observations occurred in the analytical cohort (zero variance); the variable was retained in the original feature matrix for reproducibility but provided no discriminative information.
autoimmune_pathology	History of autoimmune conditions, originally recorded using numerical diagnostic identifiers and recoded for supervised modelling as a binary presence/absence indicator (0 = absent; 1 = present). Original diagnostic identifiers were not interpreted as ordinal measurements or severity scores.	Yes	Yes	Yes	Yes	Broad immune-background variable retained in current 27-feature matrix; interpreted cautiously.
psychiatric_record / primary_diagnosis	Previously recorded psychiatric diagnosis/history and principal diagnosis at assessment; diagnostic codes identify recorded clinical categories rather than severity and were used for cohort description, not supervised prediction.	Yes	Exploratory only	No	No	Used for source-cohort characterization only.
psychological_trauma / addictions	Recorded history of psychological trauma and addiction/substance-use-related problems (separate source variables, each coded 0 = absent; 1 = present); collected for broader clinical characterization only.	Yes	Exploratory only	No	No	Not retained in final matrices.

Note: Clinical meanings describe the original source-data definitions and their subsequent analytical representation. Text-labelled categories were numerically encoded for modelling, while diagnostic identifiers for allergic/atopic and autoimmune conditions were converted into binary presence/absence indicators. These diagnostic identifiers did not represent ordinal measurements or disease-severity scores. PANS-31 item-level high-intensity indicators used a threshold >2 on the original 0–4 scale. Variables excluded from the final modelling matrices were redundant with endpoint construction, insufficiently stable, too sparse, or retained for broader descriptive characterization.

Table S9. Panel A. Operational endpoint, cohort structure, and internal partitioning.

Item	Value / definition	Interpretive note
Analytical cohort	N = 96	Harmonized baseline cohort used for observational and modelling analyses.
Source serological strata	ASO >200 IU/mL: n = 50; ASO ≤200 IU/mL: n = 46	Source sheets were ASO-stratified before harmonization.
Target variable	operational_endpoint	operational streptococcal-exposure–associated clinical–serological profile, not physician-confirmed PANDAS.
Composite endpoint definition	PANS-31 total score >36 AND ASO >200 IU/mL AND sudden_onset = 1 AND last_strep_infection_episode_months <6	Rule-based operational profile used only for internal classification.
Target distribution	Endpoint-negative: 72; endpoint-positive: 24	Endpoint-positive class represented 25.0% of the analytical cohort.
Train–test split	70/30 stratified split; random_state = 42	Same split used for full-feature and restricted-feature analyses.
Training set	n = 67; endpoint-negative = 50; endpoint-positive = 17	Used for model fitting, tuning, and scaling-parameter estimation for Logistic Regression.
Held-out test set	n = 29; endpoint-negative = 22; endpoint-positive = 7	Used for internal held-out ROC-AUC and confusion matrices.
Cross-validation	Stratified 5-fold cross-validation within GridSearchCV	Used for internal model selection; nested CV was not performed.
Optimization metric	ROC-AUC	Performance estimates are internal and not external diagnostic metrics.
Calibration / decision curve	Not formally assessed	Reported as a limitation; models are not deployable clinical tools.

Note: The supervised target was an operational streptococcal-exposure–associated clinical–serological profile and was not interpreted as physician-confirmed PANDAS.

Table S9. Panel B. Descriptive clinical characterization according to operational endpoint status.

Characteristic	Endpoint-positive (n=24)	Endpoint-negative (n=72)

Characteristic	Endpoint-positive (n=24)	Endpoint-negative (n=72)
Age, years, median [IQR]	13 [12–14]	13 [10–14]
Male sex	17 (70.8%)	44 (61.1%)
Rural residence	12 (50.0%)	41 (56.9%)
Foster-care family setting	8 (33.3%)	10 (13.9%)
ASO, IU/mL, median [IQR]	674 [586–784]	169 [103–515]
Time since last reported streptococcal infection, months, median [IQR]	3 [2–3]	11 [5–12]
PANS-31 total score, median [IQR]	44 [40–50]	26 [20–32]
Abrupt onset/worsening	24 (100.0%)	24 (33.3%)
Recurrent infections	8 (33.3%)	14 (19.4%)
Previous penicillin exposure	3 (12.5%)	18 (25.0%)
Allergic/atopic background	4 (16.7%)	12 (16.7%)
Prematurity	8 (33.3%)	13 (18.1%)
IVF history	6 (25.0%)	6 (8.3%)
Any functional digestive symptoms	17 (70.8%)	26 (36.1%)
Autoimmune pathology	4 (16.7%)	6 (8.3%)
High-intensity obsessions (>2)	7 (29.2%)	4 (5.6%)
High-intensity compulsions (>2)	7 (29.2%)	2 (2.8%)

Characteristic	Endpoint-positive (n=24)	Endpoint-negative (n=72)
High-intensity hoarding (>2)	0 (0.0%)	2 (2.8%)
High-intensity food refusal (>2)	11 (45.8%)	4 (5.6%)
High-intensity binge eating (>2)	7 (29.2%)	6 (8.3%)
High-intensity fluid refusal (>2)	1 (4.2%)	2 (2.8%)
High-intensity motor tics (>2)	14 (58.3%)	2 (2.8%)
High-intensity vocal tics (>2)	5 (20.8%)	0 (0.0%)
High-intensity enuresis (>2)	10 (41.7%)	5 (6.9%)

***Note**: Values are median [IQR] or n (%). High-intensity PANS-31 item indicators use the same prespecified item-score threshold (>2 on the original 0–4 scale) described for the modelling predictors. The endpoint-negative group comprises all participants who did not simultaneously meet all four operational endpoint conditions. Comparisons are descriptive only; no inferential hypothesis tests were performed for this subgroup characterization.*

Table S10. Data harmonization and preprocessing safeguards.

Step	Implementation	Why it matters
Column harmonization	Equivalent clinical fields from the two source sheets were aligned before endpoint construction and feature-set preparation.	Prevents duplicated or inconsistent variable representations.
Time-since-infection coding	Entries coded as >12 months were recoded as 12 and represented as integer months.	Preserves temporal ordering while avoiding a non-numeric category in a key exposure variable.
Categorical encoding	Categorical text variables were numerically encoded before model fitting. Diagnostic identifiers for allergic/atopic and autoimmune conditions were recoded into binary presence/absence indicators.	Provides a reproducible modelling matrix; no high-dimensional feature engineering was used.
Missing-data verification	Two initially blank binary entries in one participant record were verified against the original source worksheet and confirmed as absent (coded 0). No statistical imputation was performed in the final analysis-ready dataset.	Preserves the source-record coding while avoiding treatment of verified absence as missing information.
Scaling	StandardScaler was used for Logistic Regression only; Random Forest and XGBoost used encoded, unscaled features.	Scaling is required for stable regularized linear modelling but not for tree-based learners.

Step	Implementation	Why it matters
Same internal split	Full-feature and restricted-feature refits used the same stratified 70/30 train/test partition.	Makes internal performance comparisons more direct.
Class imbalance	Stratified split/CV; class_weight = balanced for Logistic Regression and Random Forest; scale_pos_weight = 2.94 for XGBoost.	Avoids synthetic oversampling while acknowledging the 50:17 training-set class ratio.
No oversampling	No SMOTE, random oversampling, or synthetic minority-sample generation was applied.	Reduces risk of synthetic-data optimism in a small cohort.

Note: Data-dependent standardization for Logistic Regression was fitted on the training partition before application to the held-out test partition. No statistical imputation was required because no unresolved missing predictor values remained in the final analysis-ready dataset.

Table S11. Hyperparameter search spaces and retained settings.

Model	Feature setting	Search grid	Retained hyperparameters	Scaling
Logistic Regression	Full-feature	C = [0.1, 1, 10]; solver fixed to liblinear; class_weight = balanced; random_state = 42; max_iter = 100	C = 0.1; solver = liblinear; class_weight = balanced; random_state = 42; max_iter = 100	StandardScaler
Logistic Regression	Restricted-feature	C = [0.1, 1, 10]; solver fixed to liblinear; class_weight = balanced; random_state = 42; max_iter = 100	C = 0.1; solver = liblinear; class_weight = balanced; random_state = 42; max_iter = 100	StandardScaler
Random Forest	Full-feature	n_estimators = [100, 200, 300]; max_depth = [3, 4, 5]; min_samples_leaf = [5, 6, 7]; max_features = [sqrt, log2]; bootstrap = True; class_weight = balanced	n_estimators = 200; max_depth = 3; min_samples_leaf = 7; max_features = log2; bootstrap = True; class_weight = balanced; random_state = 42	No
Random Forest	Restricted-feature	n_estimators = [100, 200, 300]; max_depth = [3, 4, 5]; min_samples_leaf = [5, 6, 7]; max_features = [sqrt, log2]; bootstrap = True; class_weight = balanced	n_estimators = 200; max_depth = 3; min_samples_leaf = 7; max_features = sqrt; bootstrap = True; class_weight = balanced; random_state = 42	No
XGBoost	Full-feature	scale_pos_weight = [2.94, 2.0, 1.0]; n_estimators = [10, 20, 40]; max_depth = [2, 3]; learning_rate = [0.001, 0.005, 0.01, 0.03]; colsample_bytree = [0.6, 0.8, 1.0]; colsample_bylevel = [0.6, 0.8, 1.0]	n_estimators = 10; max_depth = 2; learning_rate = 0.001; colsample_bytree = 0.6; colsample_bylevel = 0.6; scale_pos_weight = 2.94; random_state = 42	No
XGBoost	Restricted-feature	scale_pos_weight = [2.94, 2.0, 1.0];	n_estimators = 40; max_depth = 2;	No

Model	Feature setting	Search grid	Retained hyperparameters	Scaling
		n_estimators = [10, 20, 40]; max_depth = [2, 3]; learning_rate = [0.001, 0.005, 0.01, 0.03]; colsample_bytree = [0.6, 0.8, 1.0]; colsample_bylevel = [0.6, 0.8, 1.0]	learning_rate = 0.001; colsample_bytree = 0.6; colsample_bylevel = 0.8; scale_pos_weight = 2.94; random_state = 42	

Note: Hyperparameters were selected using GridSearchCV with ROC-AUC optimization and stratified 5-fold cross-validation. scale_pos_weight = 2.94 reflected the negative-to-positive class ratio in the training set.

Table S12. Complete internal performance output for full-feature and restricted-feature refit models.

Model	Feature setting	No. predictors	CV ROC-AUC	Held-out ROC-AUC (95% CI)	Sensitivity (95% CI)	Specificity (95% CI)	PPV	NPV	TN	FP	FN	TP
Logistic Regression	Full-feature	27	0.917	0.909 (0.755-1.000)	0.857 (0.421-0.996)	0.864 (0.651-0.971)	0.667	0.950	19	3	1	6
Logistic Regression	Restricted-feature	24	0.868	0.792 (0.576-1.000)	0.429 (0.099-0.816)	0.864 (0.651-0.971)	0.500	0.826	19	3	4	3
Random Forest	Full-feature	27	0.960	0.916 (0.767-1.000)	0.714 (0.290-0.963)	0.818 (0.597-0.948)	0.556	0.900	18	4	2	5
Random Forest	Restricted-feature	24	0.930	0.630 (0.380-0.880)	0.429 (0.099-0.816)	0.909 (0.708-0.989)	0.600	0.833	20	2	4	3
XGBoost	Full-feature	27	0.958	0.961 (0.857-1.000)	1.000 (0.590-1.000)	0.773 (0.546-0.922)	0.583	1.000	17	5	0	7
XGBoost	Restricted-feature	24	0.940	0.714 (0.476-0.952)	0.429 (0.099-0.816)	0.818 (0.597-0.948)	0.429	0.818	18	4	4	3

Note: Confusion-matrix counts were computed at the default 0.50 classification threshold. Sensitivity and specificity confidence intervals were calculated as exact binomial intervals. Held-out ROC-AUC confidence intervals are approximate descriptive intervals and should be interpreted cautiously because the held-out test set was small. All metrics are internal estimates only and should not be interpreted as external diagnostic-performance measures.

Table S13. Sensitivity/specificity masking check using trained full-feature models: all predictors versus selected predictors.

Model	Input scenario	Features used	TN	FP	FN	TP	Sensitivity	Specificity	PPV	NPV	ROC-AUC	Δ ROC-AUC	Δ Sensitivity
Logistic Regression	All predictors as trained	27	19	3	1	6	0.857	0.864	0.667	0.950	0.909	0.000	0.000
Logistic Regression	Selected predictors only; non-selected masked to training	24	21	1	4	3	0.429	0.955	0.750	0.840	0.760	−0.149	−0.429

Model	Input scenario	Features used	TN	FP	FN	TP	Sensitivity	Specificity	PPV	NPV	ROC-AUC	Δ ROC-AUC	Δ Sensitivity
	medians												
Random Forest	All predictors as trained	27	18	4	2	5	0.714	0.818	0.556	0.900	0.916	0.000	0.000
Random Forest	Selected predictors only; non-selected masked to training medians	24	22	0	7	0	0.000	1.000	NA	0.759	0.688	−0.227	−0.714
XGBoost	All predictors as trained	27	17	5	0	7	1.000	0.773	0.583	1.000	0.961	0.000	0.000
XGBoost	Selected predictors only; non-selected masked to training medians	24	22	0	7	0	0.000	1.000	NA	0.759	0.513	−0.448	−1.000

Note: This supplementary check was not part of the main modelling comparison. The main manuscript reports two primary analyses: full-feature models trained with all 27 retained predictors and restricted-feature models refitted from scratch after excluding continuous ASO value, infection recency, and abrupt onset. In contrast, the present masking/no-retraining check kept the trained full-feature models fixed and replaced non-selected predictors with their training-set medians. Therefore, this analysis is distinct from the restricted-feature refit analysis reported in Table S12 and should be interpreted only as an exploratory robustness check.

Table S14. Complete full-feature SHAP rankings by model family.

Rank	Logistic Regression feature	Logistic Regression mean |SHAP|	Random Forest feature	Random Forest mean |SHAP|	XGBoost feature	XGBoost mean |SHAP|
1	sudden_onset	0.395509	last_strep_infection_episode_months	0.078679	sudden_onset	0.008038
2	binary_food_refusal	0.329993	sudden_onset	0.070214	last_strep_infection_episode_months	0.002358
3	last_strep_infection_episode_months	0.281851	aslo_value	0.064256	age	0.001846
4	age	0.230291	age	0.041369	aslo_value	0.001315
5	binary_motor_tics	0.197450	binary_food_refusal	0.013932	binary_motor_tics	0.000260

Rank	Logistic Regression feature	Logistic Regression mean |SHAP|	Random Forest feature	Random Forest mean |SHAP|	XGBoost feature	XGBoost mean |SHAP|
6	environment	0.162977	binary_motor_tics	0.011859	binary_food_refusal	0.000258
7	aslo_value	0.162161	functional_digestive_symptoms	0.011136	penicillin_treatment	0.000164
8	penicillin_treatment	0.157323	sex	0.007243	binary_vocal_tics	0.000124
9	source	0.151441	recurrent_infections	0.005793	binary_enuresis	0.000000
10	binary_binge_eating	0.151103	penicillin_treatment	0.005573	rheumatic_fever_chorea_endocarditis	0.000000
11	family	0.141667	family	0.004683	viral_infections	0.000000
12	binary_obsessions	0.109829	environment	0.004347	bacterial_infections_non_strep	0.000000
13	binary_vocal_tics	0.100472	binary_enuresis	0.003881	functional_digestive_symptoms	0.000000
14	binary_compulsions	0.095726	source	0.002717	ivf	0.000000
15	viral_infections	0.087481	binary_binge_eating	0.001395	prematurity	0.000000
16	ivf	0.084508	prematurity	0.001331	allergies	0.000000
17	bacterial_infections_non_strep	0.049724	bacterial_infections_non_strep	0.000000	binary_fluid_refusal	0.000000
18	recurrent_infections	0.049344	viral_infections	0.000000	sex	0.000000
19	functional_digestive_symptoms	0.036471	rheumatic_fever_chorea_endocarditis	0.000000	binary_binge_eating	0.000000
20	binary_fluid_refusal	0.032075	ivf	0.000000	binary_hoarding	0.000000
21	sex	0.019328	binary_fluid_refusal	0.000000	binary_compulsions	0.000000
22	binary_hoarding	0.018963	allergies	0.000000	binary_obsessions	0.000000
23	prematurity	0.017742	binary_vocal_tics	0.000000	recurrent_infections	0.000000
24	binary_enuresis	0.009010	binary_hoarding	0.000000	source	0.000000
25	allergies	0.000117	binary_compulsions	0.000000	family	0.000000
26	autoimmune_pathology	0.000018	binary_obsessions	0.000000	environment	0.000000
27	rheumatic_fever_chorea_endocarditis	0.000000	autoimmune_pathology	0.000000	autoimmune_pathology	0.000000

Note: SHAP magnitudes are model-specific and should not be compared directly across model families. Rankings describe model attribution within the internal analysis only.

Table S15. Complete restricted-feature SHAP rankings by model family.

Rank	Logistic Regression feature	Logistic Regression mean |SHAP|	Random Forest feature	Random Forest mean |SHAP|	XGBoost feature	XGBoost mean |SHAP|
1	binary_food_refusal	0.410091	age	0.066900	age	0.012105
2	binary_binge_eating	0.256740	binary_food_refusal	0.028064	binary_food_refusal	0.012086
3	age	0.229293	functional_digestive_symptoms	0.025227	binary_binge_eating	0.006362
4	binary_motor_tics	0.216296	sex	0.017419	binary_motor_tics	0.003880
5	penicillin_treatment	0.198338	binary_motor_tics	0.016518	binary_compulsions	0.001468
6	environment	0.181726	environment	0.011552	functional_digestive_symptoms	0.001348
7	family	0.153023	penicillin_treatment	0.010838	binary_obsessions	0.001114
8	binary_obsessions	0.130986	family	0.010496	binary_enuresis	0.000879
9	binary_vocal_tics	0.125229	source	0.006854	binary_vocal_tics	0.000491
10	functional_digestive_symptoms	0.119331	recurrent_infections	0.006550	allergies	0.000241
11	binary_compulsions	0.114744	binary_enuresis	0.005688	ivf	0.000228
12	bacterial_infections_non_strep	0.101528	prematurity	0.003266	recurrent_infections	0.000094
13	viral_infections	0.099195	binary_binge_eating	0.001501	rheumatic_fever_chorea_endocarditis	0.000000
14	ivf	0.095792	allergies	0.001179	viral_infections	0.000000
15	source	0.094508	binary_fluid_refusal	0.000000	bacterial_infections_non_strep	0.000000
16	sex	0.083100	binary_vocal_tics	0.000000	prematurity	0.000000
17	binary_enuresis	0.048220	binary_obsessions	0.000000	source	0.000000
18	binary_fluid_refusal	0.047523	binary_hoarding	0.000000	penicillin_treatment	0.000000
19	prematurity	0.039492	ivf	0.000000	sex	0.000000
20	recurrent_infections	0.039488	binary_compulsions	0.000000	binary_fluid_refusal	0.000000
21	binary_hoarding	0.028096	bacterial_infections_non_strep	0.000000	environment	0.000000
22	allergies	0.027260	viral_infections	0.000000	family	0.000000
23	autoimmune_pathology	0.008216	rheumatic_fever_chorea_endocarditis	0.000000	binary_hoarding	0.000000
24	rheumatic_fever_chorea_endocarditis	0.000000	autoimmune_pathology	0.000000	autoimmune_pathology	0.000000

Note: The restricted-feature SHAP outputs exclude continuous ASO value, infection recency, and abrupt onset because those variables were not included in the restricted-feature refit models.

Table S16. Interpretive safeguards and reporting limitations.

Domain	Implementation	Interpretive boundary
Endpoint interpretation	operational streptococcal-exposure–associated clinical–serological profile	Not physician-confirmed PANDAS; not a validated diagnostic category.
Clinical-information setting	Full-feature complete-information baseline modelling	Reflects information clinicians may have at assessment; not independent diagnostic prediction.
Predictor–endpoint proximity	Continuous ASO value, infection recency, abrupt onset, and PANS-31 item-level variables are conceptually related to endpoint components	Full-feature performance represents internal profile reconstruction.
Restricted-feature refit	ASO value, infection recency, and abrupt onset excluded and models refitted from scratch	Estimates residual symptom/context signal, not a new diagnostic model.
Masking sensitivity	Already trained full-feature models evaluated after non-selected predictors were replaced by training medians	Assesses dependence of fitted full-feature models on masked variables; not equivalent to refit performance.
Validation status	Internal 70/30 held-out split and stratified 5-fold CV	No external validation; performance may be optimistic/unstable.
Calibration	Not formally assessed	No calibration plot, calibration slope, or Brier score; probability estimates not deployable.
Clinical utility	Not formally assessed	No decision-curve analysis or threshold optimization; no clinical deployment.
SHAP interpretation	Global and model-specific feature attribution	Describes model behaviour; does not establish causality, biological mechanism, disease specificity, or patient-level diagnosis.
Data disclosure	Aggregated supplementary data only	No participant-level records included.

Note: These safeguards align the supplementary file with the manuscript framing: internal complete-information phenotype-structure reconstruction, not diagnostic prediction or clinical deployment.

Table S17. TRIPOD+AI-oriented supplementary reporting crosswalk.

Reporting area	Supplementary coverage
Study objective and modelling task	The relationship between the descriptive observational phase and the present supervised modelling phase is summarized in Table S2. The operational endpoint, target distribution, and feature-setting rationale are reported in Tables S9–S10 and Figures S2–S3.
Data source and participants	Participant flow, analytical retention, exclusion reasons, ASO-stratified cohort structure, and train–test partitioning are summarized in Table S1, Table S9, and Figure S1.
Outcome / endpoint	The composite operational endpoint definition, target variable, class distribution, and

	interpretive boundary are reported in Table S9. The endpoint was defined as PANS-31 total score >36, ASO >200 IU/mL, abrupt onset or abrupt worsening, and infection recency <6 months.
Predictors and feature handling	The extended variable dictionary, candidate predictor handling, retained predictors, excluded variables, and rationale for full-feature and restricted-feature matrices are reported in Tables S8–S10.
Missing data and preprocessing	Data harmonization, source-record verification of initially blank binary entries, coding of time since infection, categorical encoding, scaling, class-imbalance handling, and preprocessing safeguards are summarized in Table S10.
Model specification	Algorithms, hyperparameter search grids, retained hyperparameters, scaling strategy, and class-imbalance settings are reported in Table S11.
Performance measures	Complete internal performance output, including cross-validated ROC-AUC, held-out ROC-AUC, sensitivity, specificity, PPV, NPV, and confusion-matrix counts, is reported in Table S12.
Sensitivity analyses	The restricted-feature refit performance is reported in Table S12. The masking/no-retraining sensitivity check is reported in Table S13. The analytical distinction between complete-information modelling, restricted-feature refit, and masking sensitivity is summarized in Figure S3.
Explainability	Complete SHAP-ranked feature-attribution outputs for full-feature and restricted-feature models are provided in Tables S14–S15. The interpretive limits of SHAP-based attribution are summarized in Table S16.
Limitations and applicability	Interpretive safeguards, lack of external validation, lack of calibration assessment, lack of decision-curve analysis, limited subgroup/fairness evaluation, and non-deployability are summarized in Table S16.
Code sharing	The original Python analytical workflow is provided as an additional supplementary HTML file, including the exploratory analyses, data preparation, model development, internal evaluation, and SHAP-based interpretation. The participant-level clinical dataset is not included. No deployable model object, API, or clinical decision-support software is provided.

Note: This crosswalk helps reviewers locate methodological details that are necessarily abbreviated in the main manuscript.

Supplementary Workflow Figures

Figure S1. Participant flow, source-cohort structure, and internal modelling partition.

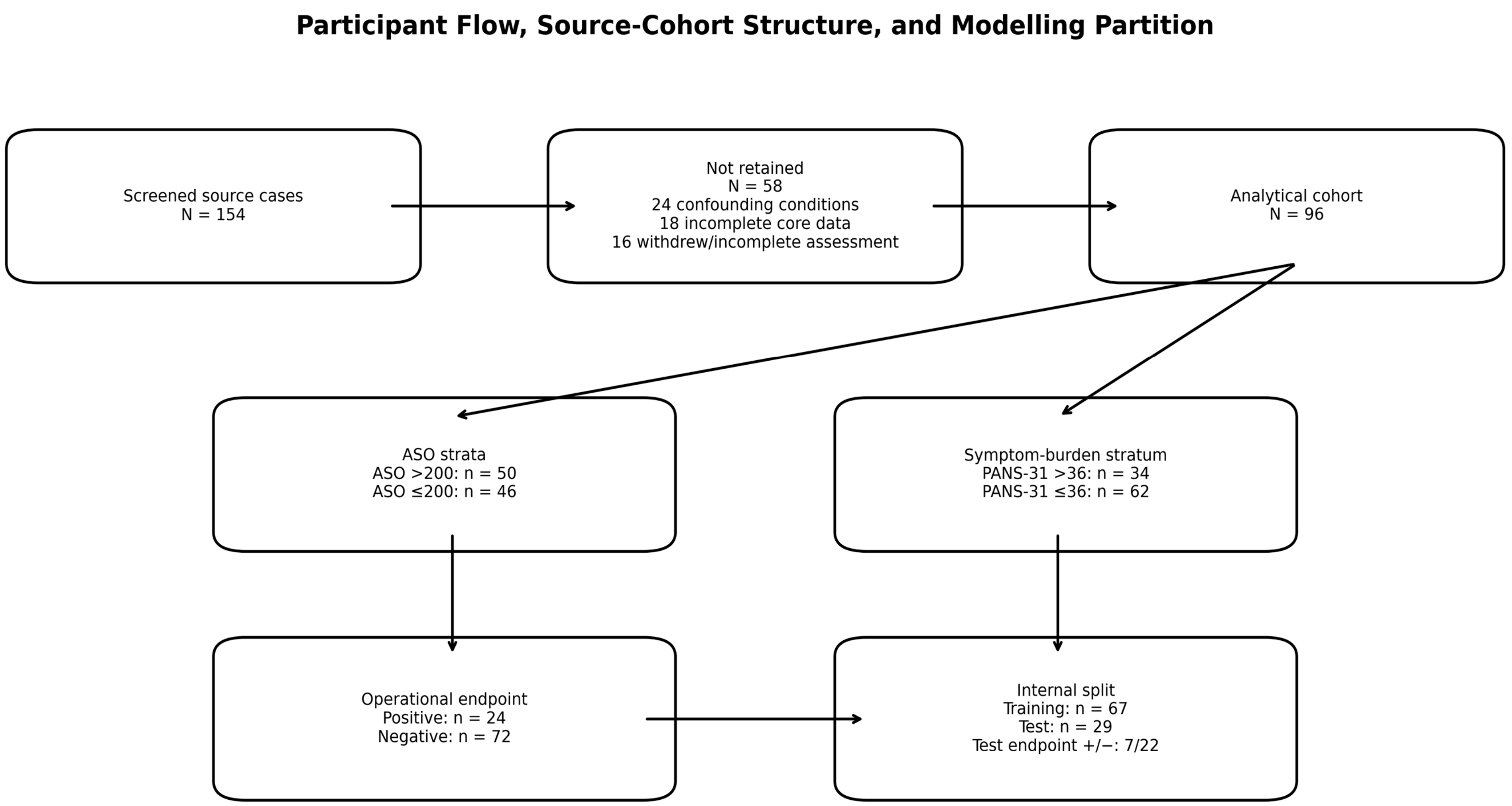

The figure summarizes screening, non-retention, analytical retention, ASO and PANS-31 descriptive strata, operational endpoint distribution, and the stratified train–test split.

Figure S2. Analytical workflow linking descriptive source-cohort context to internal supervised modelling.

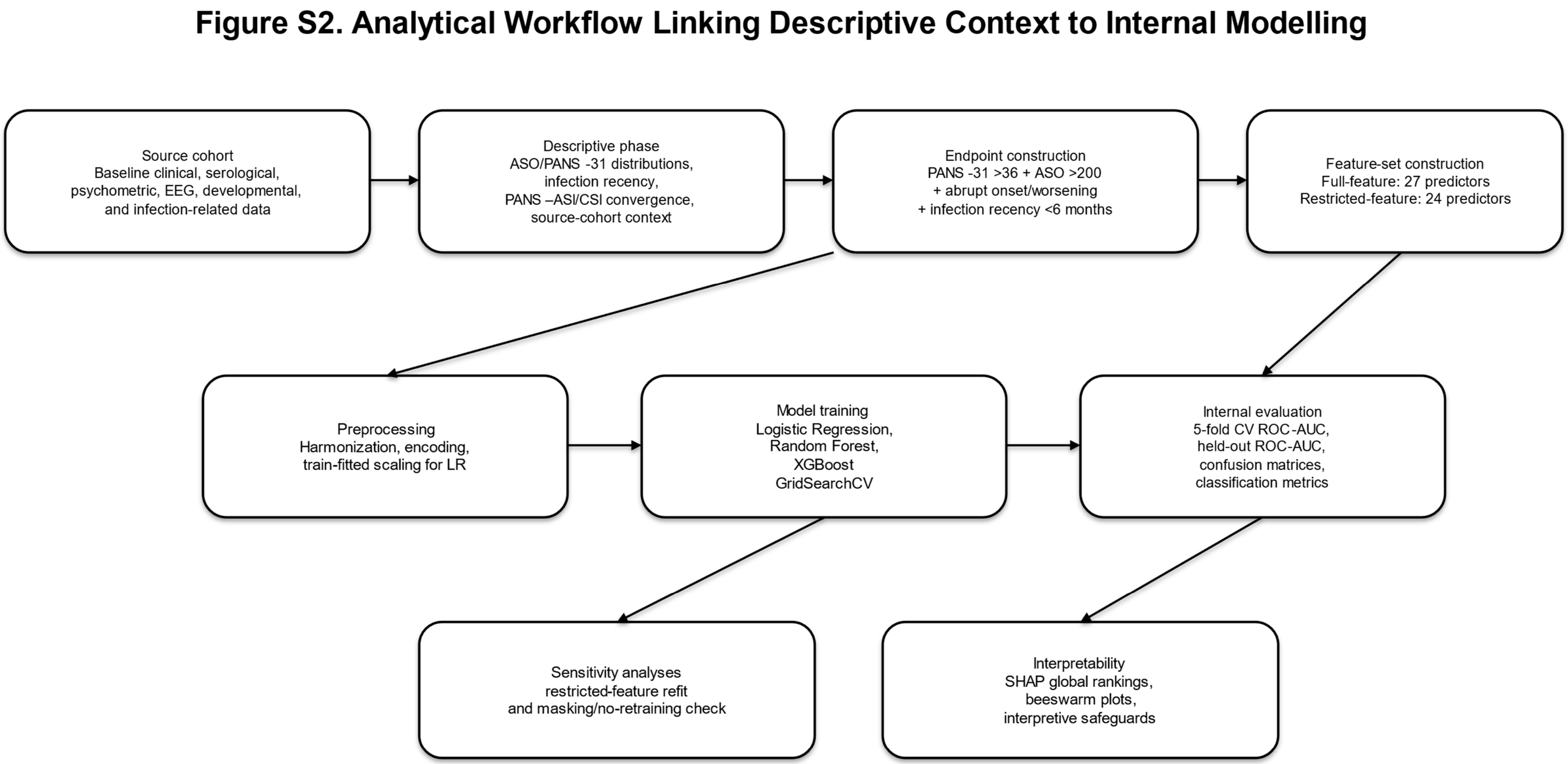

The figure shows how the source-cohort descriptive phase supports endpoint construction and feature-set preparation before model training, internal evaluation, sensitivity analysis, and SHAP interpretation.

Figure S3. Feature-set and sensitivity-analysis logic.

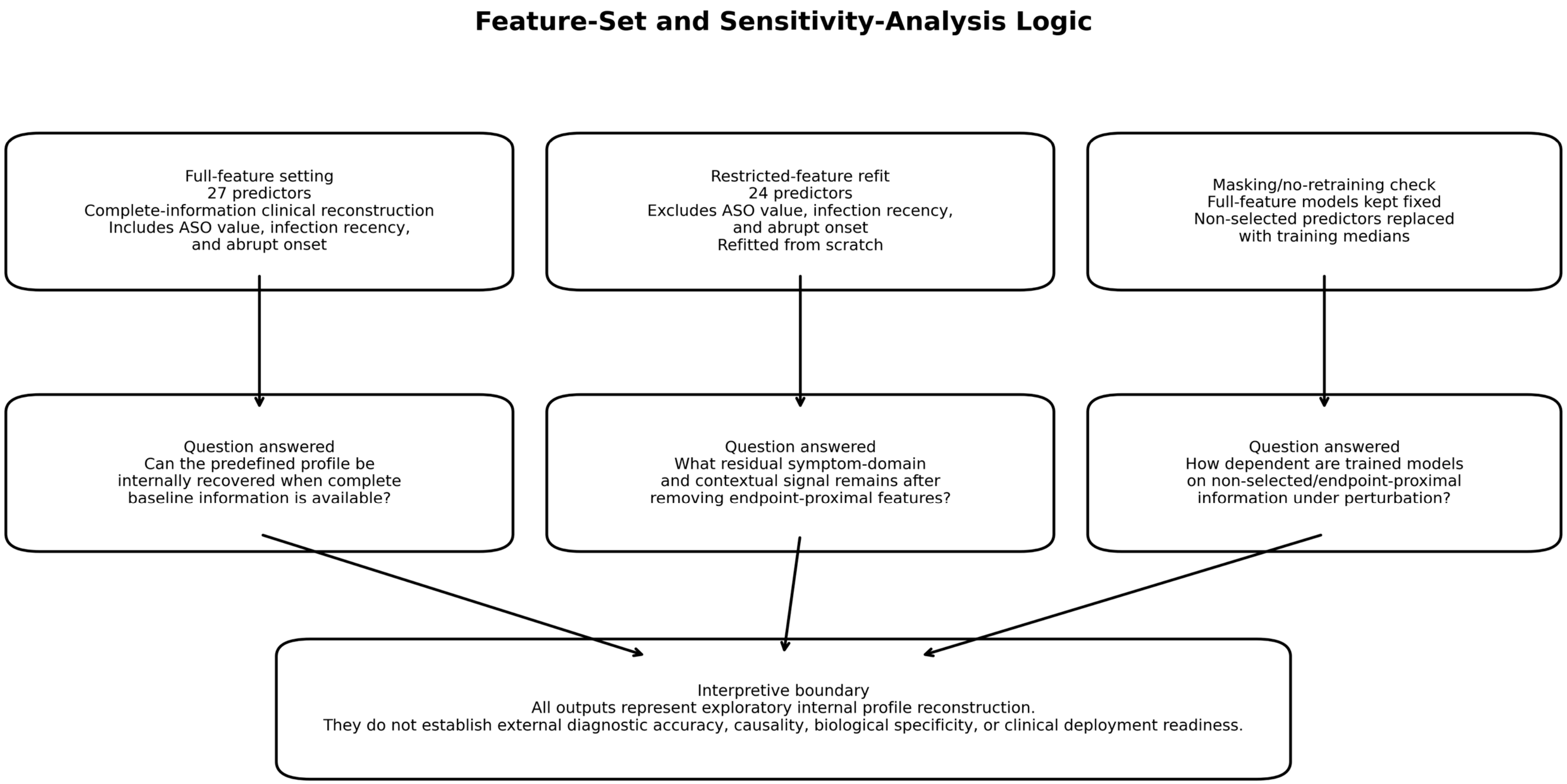

The figure distinguishes the full-feature complete-information setting, the restricted-feature refit analysis, and the masking/no-retraining sensitivity check.

Abbreviations

ASO, antistreptolysin O; ASI/CSI, Adolescent Symptom Inventory/Child Symptom Inventory; CV, cross-validation; EEG, electroencephalography; FDR, false-discovery rate; FN, false negative; FP, false positive; IVF, in vitro fertilization; LR, Logistic Regression; ML, machine learning; NPV, negative predictive value; PANS-31, Pediatric Acute-onset Neuropsychiatric Syndrome 31-Item Symptom Rating Scale; PANDAS, Pediatric Autoimmune Neuropsychiatric Disorders Associated with Streptococcal Infections; PPV, positive predictive value; RF, Random Forest; ROC-AUC, receiver operating characteristic area under the curve; SHAP, SHapley Additive exPlanations; TN, true negative; TP, true positive; XGBoost, Extreme Gradient Boosting.
