# Supplementary material for: Interpretable Modelling of a Streptococcal Exposure–Associated Neuropsychiatric Profile in a Pediatric Cohort
Source: Children (Basel). 2026 Oct 21;13(10):1443. doi: 10.3390/children13101443 (PMC13703277; doi:10.3390/children13101443)
Supplement: Supplementary file 1 [file children-13-01443-s001.zip › S3. TRIPODAI_checklist_AI_Model.pdf]

Version: 11-January-2024

Article: Interpretable Modelling of a Streptococcal-Exposure–Associated Neuropsychiatric Profile in a Pediatric Cohort

Section/Topic	Item	Development / evaluation[1]	Checklist item	Reported on page
**TITLE**				
Title	1	D;E	Identify the study as developing or evaluating the performance of a multivariable prediction model, the target population, and the outcome to be predicted	Title page.
**ABSTRACT**				
Abstract	2	D;E	See TRIPOD+AI for Abstracts checklist	Abstract
**INTRODUCTION**				
Background	3a	D;E	Explain the healthcare context (including whether diagnostic or prognostic) and rationale for developing or evaluating the prediction model, including references to existing models	Introduction; Methods 2.1.
	3b	D;E	Describe the target population and the intended purpose of the prediction model in the context of the care pathway, including its intended users (e.g., healthcare professionals, patients, public)	Introduction; Methods 2.1–2.2; Discussion 4.9–4.10. Exploratory research use only; no clinical implementation pathway or intended end-user workflow was proposed.
	3c	D;E	Describe any known health inequalities between sociodemographic groups	Methods 2.8; Discussion 4.9; Supplementary Table S16. No subgroup-specific preprocessing or formal health-inequality/fairness analysis was performed; this is acknowledged as a limitation.
Objectives	4	D;E	Specify the study objectives, including whether the study describes the development or validation of a prediction model (or both)	Introduction, final paragraphs; Methods 2.1.
**METHODS**				
Data	5a	D;E	Describe the sources of data separately for the development and evaluation datasets (e.g., randomised trial, cohort, routine care or registry data), the rationale for using these data, and representativeness of the data	Methods 2.1–2.2; Supplementary Tables S1–S2, S9.
	5b	D;E	Specify the dates of the collected participant data, including start and end of participant accrual; and, if applicable, end of follow-up	Methods 2.2.
Participants	6a	D;E	Specify key elements of the study setting (e.g., primary care, secondary care, general population)<br>including the number and location of centres	Methods 2.2.
	6b	D;E	Describe the eligibility criteria for study participants	Methods 2.2; Supplementary Table S1.
	6c	D;E	Give details of any treatments received, and how they were handled during model development or evaluation, if relevant	Methods 2.2–2.3; Statements.
Data preparation	7	D;E	Describe any data pre-processing and quality checking, including whether this was similar across<br>relevant sociodemographic groups	Methods 2.8; Supplementary Table S10. The same preprocessing workflow was applied across participants; preprocessing was not tailored by sociodemographic subgroup.
Outcome	8a	D;E	Clearly define the outcome that is being predicted and the time horizon, including how and when assessed, the rationale for choosing this outcome, and whether the method of outcome assessment is<br>consistent across sociodemographic groups	Methods 2.4; Supplementary Table S9. The endpoint was derived from baseline data; the modelling task was cross-sectional and no prospective prognostic time horizon was defined.
	8b	D;E	If outcome assessment requires subjective interpretation, describe the qualifications and demographic characteristics of the outcome assessors	Methods 2.4. Not applicable to endpoint adjudication: the endpoint was algorithmically derived from predefined baseline variables and was not independently adjudicated.
	8c	D;E	Report any actions to blind assessment of the outcome to be predicted	Methods 2.4; no blinded outcome adjudication, because the endpoint was rule-based.
Predictors	9a	D	Describe the choice of initial predictors (e.g., literature, previous models, all available predictors) and<br>any pre-selection of predictors before model building	Methods 2.5–2.7; Table 1; Supplementary Tables S8–S9.
	9b	D;E	Clearly define all predictors, including how and when they were measured (and any actions to blind assessment of predictors for the outcome and other predictors)	Methods 2.3, 2.6–2.7; Table 1; Supplementary Table S8.
	9c	D;E	If predictor measurement requires subjective interpretation, describe the qualifications and demographic characteristics of the predictor assessors	Methods 2.3–2.4; predictors derived from caregiver report, records, psychometrics and clinical documentation.
Sample size	10	D;E	Explain how the study size was arrived at (separately for development and evaluation), and justify that<br>the study size was sufficient to answer the research question. Include details of any	Methods 2.1; Results 3.1; Supplementary Tables S1 and S9<br>No a priori sample-size

			sample size calculation	calculation was performed because this was a secondary exploratory analysis of an existing cohort. The available analytical sample comprised N=96; its limited size and the corresponding implications for model stability and overfitting are explicitly discussed as limitations.
Missing data	11	D;E	Describe how missing data were handled. Provide reasons for omitting any data	Methods 2.8; Supplementary Table S10. Two initially blank binary entries in one participant record were source-verified and coded as absence (0). No unresolved missing predictor values remained in the final analysis-ready dataset; no participant-level statistical imputation was required.
Analytical methods	12a	D	Describe how the data were used (e.g., for development and evaluation of model performance) in the analysis, including whether the data were partitioned, considering any sample size requirements	Methods 2.8–2.9; Results 3.1; Supplementary Tables S9–S12.
	12b	D	Depending on the type of model, describe how predictors were handled in the analyses (functional form, rescaling, transformation, or any standardisation).	Methods 2.6–2.9; Tables 1–2; Supplementary Tables S8–S11.
	12c	D	Specify the type of model, rationale[2], all model-building steps, including any hyperparameter tuning, and method for internal validation	Methods 2.9–2.11; Table 2; Supplementary Table S11.
	12d	D;E	Describe if and how any heterogeneity in estimates of model parameter values and model performance was handled and quantified across clusters (e.g., hospitals, countries). See TRIPOD-Cluster for additional considerations[3]	Not applicable; no clustered or multicentre performance heterogeneity analysis.
	12e	D;E	Specify all measures and plots used (and their rationale) to evaluate model performance (e.g., discrimination, calibration, clinical utility) and, if relevant, to compare multiple models	Methods 2.9–2.10; Results 3.3–3.4; Table 5; Figures 1–7; Supplementary Tables S12–S15 clinical-utility analyses were not performed and are addressed as limitations.
	12f	E	Describe any model updating (e.g., recalibration) arising from the model evaluation, either overall or for particular sociodemographic groups or settings	Not applicable; no model updating or recalibration was performed.
	12g	E	For model evaluation, describe how the model predictions were calculated (e.g., formula, code, object, application programming interface)	Methods 2.9–2.11; Table 2; Supplementary Tables S11–S12; no deployable model object, API, or clinical decision-support software is provided.
Class imbalance	13	D;E	If class imbalance methods were used, state why and how this was done, and any subsequent methods to recalibrate the model or the model predictions	Methods 2.9; Table 2; Supplementary Tables S10–S11.
Fairness	14	D;E	Describe any approaches that were used to address model fairness and their rationale	Methods 2.8; Discussion 4.9; Supplementary Table S16.
Model output	15	D	Specify the output of the prediction model (e.g., probabilities, classification). Provide details and rationale for any classification and how the thresholds were identified	Methods 2.9; Results 3.3; Table 5; Figure 2; Supplementary Table S12.

[1] D=items relevant only to the development of a prediction model; E=items relating solely to the evaluation of a prediction model; D;E=items applicable to both the development and evaluation of a prediction model
[2] Separately for all model building approaches.
[3] TRIPOD-Cluster is a checklist of reporting recommendations for studies developing or validating models that explicitly account for clustering or explore heterogeneity in model performance (eg, at different hospitals or centres). Debray et al, BMJ 2023; 380: e071018 [DOI: 10.1136/bmj-2022-071018]

Training versus evaluation	16	D;E	Identify any differences between the development and evaluation data in healthcare setting, eligibility criteria, outcome, and predictors	Methods 2.8–2.9; Results 3.1; Supplementary Table S9. Same source cohort and split used.
Ethical approval	17	D;E	Name the institutional research board or ethics committee that approved the study and describe the participant-informed consent or the ethics committee waiver of informed consent	Institutional Review Board Statement and Informed Consent Statement.
**OPEN SCIENCE**				
Funding	18a	D;E	Give the source of funding and the role of the funders for the present study	Funding statement. No funding was received
Conflicts of interest	18b	D;E	Declare any conflicts of interest and financial disclosures for all authors	Conflicts of Interest statement – no conflict of interest
Protocol	18c	D;E	Indicate where the study protocol can be accessed or state that a protocol was not prepared	Data Availability / Study Registration statement.
Registration	18d	D;E	Provide registration information for the study, including register name and registration number, or state that the study was not registered	Data Availability / Study Registration statement.
Data sharing	18e	D;E	Provide details of the availability of the study data	Data Availability Statement.
Code sharing	18f	D;E	Provide details of the availability of the analytical code[4]	The original Python analytical workflow is provided as an additional supplementary HTML file. Access to participant-level clinical data is restricted as described in the Data Availability Statement. No deployable model object, API, or clinical decision-support software is provided.
**PATIENT & PUBLIC INVOLVEMENT**				
Patient & Public Involvement	19	D;E	Provide details of any patient and public involvement during the design, conduct, reporting, interpretation, or dissemination of the study or state no involvement.	Informed Consent Statement/ no formal patient or public involvement was undertaken for the present secondary modelling analysis.
**RESULTS**				
Participants	20a	D;E	Describe the flow of participants through the study, including the number of participants with and without the outcome and, if applicable, a summary of the follow-up time. A diagram may be helpful.	Results 3.1; Table 3; Supplementary Table S1; Figure S1.
	20b	D;E	Report the characteristics overall and, where applicable, for each data source or setting, including the key dates, key predictors (including demographics), treatments received, sample size, number of outcome events, follow-up time, and amount of missing data. A table may be helpful. Report any differences across key demographic groups.	Results 3.1–3.2; Tables 3–4; Supplementary Tables S3–S6, S9–S10.
	20c	E	For model evaluation, show a comparison with the development data of the distribution of important predictors (demographics, predictors, and outcome).	Results 3.1; Table 3; Supplementary Table S9. No external evaluation dataset; internal held-out split only.
Model development	21	D;E	Specify the number of participants and outcome events in each analysis (e.g., for model development, hyperparameter tuning, model evaluation)	Results 3.1 and 3.3; Tables 3 and 5; Supplementary Table S12.
Model specification	22	D	Provide details of the full prediction model (e.g., formula, code, object, application programming interface) to allow predictions in new individuals and to enable third-party evaluation and implementation, including any restrictions to access or re-use (e.g., freely available, proprietary)[5]	Methods 2.9–2.11; Table 2; Supplementary Tables S11 and S16.; no deployable prediction formula, model object, API, or clinical decision-support tool is provided.
Model performance	23a	D;E	Report model performance estimates with confidence intervals, including for any key subgroups (e.g., sociodemographic). Consider plots to aid presentation.	Results 3.3; Table 5; Figures 1–3; Supplementary Table S12. Descriptive 95% confidence intervals for held-out ROC-AUC, sensitivity, and specificity are reported in Supplementary Table S12; uncertainty and limited test-set size are discussed in Results and Limitations.
	23b	D;E	If examined, report results of any heterogeneity in model performance across clusters. See TRIPOD Cluster for additional details[3].	Not applicable; no cluster-level heterogeneity analysis was performed.
Model updating	24	E	Report the results from any model updating, including the updated model and subsequent performance	Not applicable; no model updating was performed.

DISCUSSION				
Interpretation	25	D;E	Give an overall interpretation of the main results, including issues of fairness in the context of the objectives and previous studies	Discussion 4.1–4.8; Supplementary Table S16.
Limitations	26	D;E	Discuss any limitations of the study (such as a non-representative sample, sample size, overfitting, missing data) and their effects on any biases, statistical uncertainty, and generalizability	Discussion 4.9; Supplementary Table S16.
Usability of the model in the context of current care	27a	D	Describe how poor quality or unavailable input data (e.g., predictor values) should be assessed and handled when implementing the prediction model	Discussion 4.9–4.10; Supplementary Table S16. No implementation pathway proposed.
	27b	D	Specify whether users will be required to interact in the handling of the input data or use of the model, and what level of expertise is required of users	Discussion 4.9–4.10; Supplementary Table S16. No user interaction or deployment proposed.
	27c	D;E	Discuss any next steps for future research, with a specific view to applicability and generalizability of the model	Discussion 4.10; Conclusions.

From: Collins GS, Moons KGM, Dhiman P, et al. *BMJ* 2024;385:e078378. doi:10.1136/bmj-2023-078378

[4] This relates to the analysis code, for example, any data cleaning, feature engineering, model building, evaluation.
[5] This relates to the code to implement the model to get estimates of risk for a new individual.
